# Tunable, division-independent control of gene activation timing by a polycomb switch

**DOI:** 10.1016/j.celrep.2021.108888

**Published:** 2021-03-23

**Authors:** Nicholas A. Pease, Phuc H.B. Nguyen, Marcus A. Woodworth, Kenneth K.H. Ng, Blythe Irwin, Joshua C. Vaughan, Hao Yuan Kueh

**Affiliations:** 1 Department of Bioengineering, University of Washington, Seattle, WA 98195, USA; 2 Molecular and Cellular Biology Program, University of Washington, Seattle, WA 98195, USA; 3 Molecular Engineering and Sciences Institute, University of Washington, Seattle, WA 98195, USA; 4 Biological Physics, Structure and Design Program, University of Washington, Seattle, WA 98195, USA; 5 Department of Chemistry, University of Washington, Seattle, WA 98195, USA; 6 Department of Physiology and Biophysics, University of Washington, Seattle, WA 98195, USA; 7 Institute for Stem Cell and Regenerative Medicine, University of Washington, Seattle, WA 98195, USA; 8 These authors contributed equally; 9 Lead contact

## Abstract

During development, progenitors often differentiate many cell generations after receiving signals. These delays must be robust yet tunable for precise population size control. Polycomb repressive mechanisms, involving histone H3 lysine-27 trimethylation (H3K27me3), restrain the expression of lineage-specifying genes in progenitors and may delay their activation and ensuing differentiation. Here, we elucidate an epigenetic switch controlling the T cell commitment gene *Bcl11b* that holds its locus in a heritable inactive state for multiple cell generations before activation. Integrating experiments and modeling, we identify a mechanism where H3K27me3 levels at *Bcl11b*, regulated by methyltransferase and demethylase activities, set the time delay at which the locus switches from a compacted, silent state to an extended, active state. This activation delay robustly spans many cell generations, is tunable by chromatin modifiers and transcription factors, and is independent of cell division. With their regulatory flexibility, such timed epigenetic switches may broadly control timing in development.

## In brief

H3K27me3 is found on repressed lineage-specifying gene loci in stem cells and may delay their activation in response to signals. Pease et al. identify an adjustable timer mechanism, where H3K27me3 levels at the T cell commitment gene *Bcl11b*, set by opposing writer and eraser enzymes, modulate the timing of gene locus decompaction and activation.

## Graphical abstract

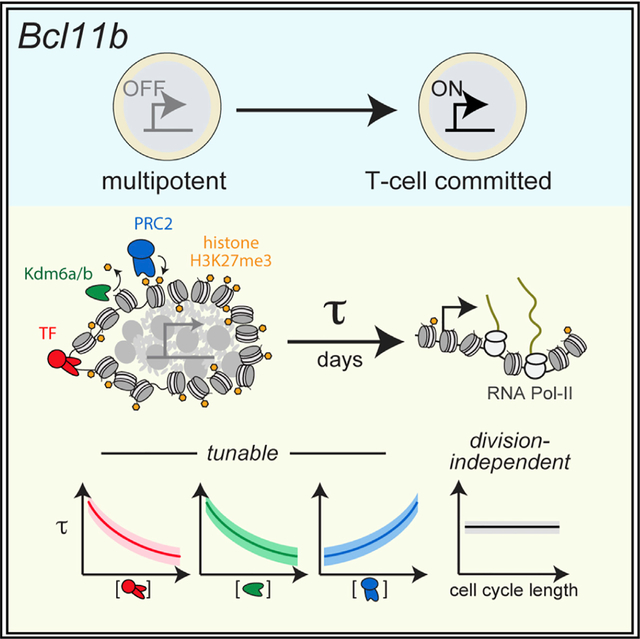

## INTRODUCTION

During multicellular development, stem and progenitor cells often differentiate many days and cell divisions after receiving instructive signals. These differentiation delays must be robust yet tunable over timescales spanning multiple cell generations for precise control over differentiated population sizes. In diverse contexts, cell differentiation delays are generated by timing mechanisms operating autonomously in single cells. In classic studies of oligodendrocyte differentiation, precursor cells exposed to signals delayed their differentiation by up to eight cell divisions, due to a cell-autonomous timing mechanism (Gao et al., 1997; Temple and Raff, 1986). Such autonomous timing control is seen in diverse systems, from brain and muscle development to adaptive immunity (Burton et al., 1999; Heinzel et al., 2017; Otani et al., 2016).

Although timing delays in some embryonic systems are generated by mechanisms that count cell divisions (Amodeo et al., 2015; Newport and Kirschner, 1982), differentiation delays during later vertebrate development or in adult stem cells are often uncoupled from cell cycle progression, such that changes to rates of cell division do not affect delay duration (Burton et al., 1999; Gao et al., 1997; Heinzel et al., 2017; Li et al., 2019; Okamoto et al., 2016; Osmond, 1991; Otani et al., 2016). A mechanism for setting the elapsed time to differentiation apart from cell division could provide functional advantages to cells, including operation in non-dividing cells and an ability to modulate cell expansion while maintaining a constant temporal schedule for differentiation. However, it is unknown how division-independent timing control is implemented on a molecular level.

Polycomb repressive mechanisms, involving histone H3 lysine-27 trimethylation (H3K27me3), are important for differentiation timing control. H3K27me3 modifications are often found at the loci of lineage-specifying genes in stem cells, where they restrain the expression of these genes and resultant differentiation (Boyer et al., 2006; Lee et al., 2006). During differentiation, instructive signals activate transcription factors that bind to lineage-specifying genes and initiate H3K27me3 removal. However, although transcription factors usually bind rapidly upon signal exposure within minutes, H3K27me3 loss and gene activation often occur much more slowly, such that gene loci can heritably maintain a silent state over multiple cell generations prior to activation (Berry et al., 2017; Kaikkonen et al., 2013; Bintu et al., 2016; Mayran et al., 2018). In a prevailing view, this epigenetic maintenance of the silent state before activation results from the passive dilution of H3K27me3 modified histones through serial cell division (Gaydos et al., 2014; Jadhav et al., 2020; Sun et al., 2014). However, although passive dilution mechanisms can delay differentiation over multiple cell divisions, these delays would depend on cell cycle duration and be inconsistent with the division-independent timers found in vertebrates. Alternatively, H3K27me3 loss may be actively controlled by the opposing polycomb repressive complex 2 (PRC2) methyltransferase and Kdm6a/b demethylase activities (Li et al., 2014; Park et al., 2014; Seenundun et al., 2010). However, it is unclear whether such active mechanisms could generate differentiation delays spanning many cell divisions and whether these delays could be both tunable and cell-division independent.

**Figure 1.**
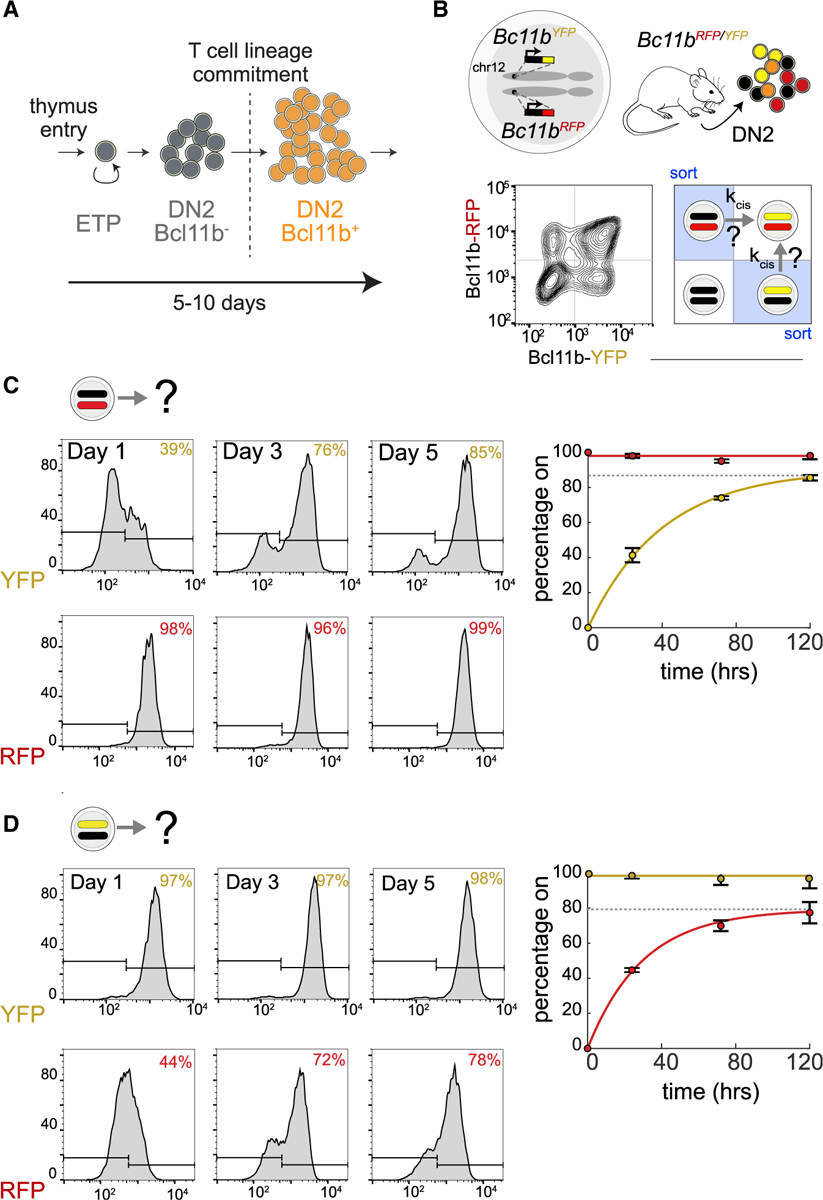
A timed epigenetic switch delays *Bcl11b* activation and T cell lineage commitment (A) *Bcl11b*, a transcription factor that drives T cell lineage commitment, turns on with a multi-day time delay. (B) Dual-allelic *Bcl11b* reporter mouse (top), along with flow cytometry plot showing levels of each *Bcl11b* allele in DN2 progenitors (bottom left) and with strategy to purify *Bcl11b* monoallelic expressing progenitors for live-cell analysis of epigenetic switch timing, k_*cis*_. (C and D) DN2 monoallelic *Bcl11b*-expressing progenitors were purified, cultured on OP9-DL1 feeders with 5 ng/mL interleukin-7 (IL-7) and Flt3L, and analyzed by flow cytometry. Data represent means and 95% confidence intervals for n = 3 independent experiments. Curves represent fits to the equation y = F(1 − e^−kt^), where F is the final percentage of cells positive for assayed allele (represented by the dotted gray lines); k = 0.025 h^−1^ ± 0.005 for YFP activation and k = 0.034 h^−1^ ± 0.009 for RFP activation.

To address these questions, we investigated the mechanism of a time-delayed epigenetic switch controlling the activation of *Bcl11b*, a transcription factor essential for T cell lineage commitment and identity (Hosokawa et al., 2018; Ikawa et al., 2010; Li et al., 2010; Figure 1A). In early T cell progenitors, Notch signals activate *Bcl11b*, both directly (Ikawa et al., 2010; Li et al., 2010) and indirectly, by activating its upstream regulators Gata3 and TCF-1 (García-Ojeda et al., 2013; Germar et al., 2011; Zhou et al., 2019). However, although these upstream regulators become active shortly after thymic entry, *Bcl11b* activation and T cell lineage commitment occur ~5–10 days later, during which progenitors proliferate 1,000-fold (Manesso et al., 2013; Porritt et al., 2003; Zhou et al., 2019). Using a dual-allele *Bcl11b* reporter strain, where each endogenous gene copy is tagged with distinguishable fluorescent protein reporters (Ng et al., 2018), we found that this long delay in *Bcl11b* activation arises partly because of an epigenetic switch acting at individual *Bcl11b* loci, in *cis*. This epigenetic switch activates probabilistically, with a multi-day time constant that is controlled by Gata3 and TCF-1 (Kueh et al., 2016), along with a distal enhancer to which these factors bind (Ng et al., 2018). However, our previous studies did not clarify the mechanism of the timed epigenetic switch itself or its basis for generating controllable timing delays in gene activation. We address these outstanding questions here.

## RESULTS

### A timed epigenetic switch delays *Bcl11b* activation and T cell lineage commitment

To study the *cis*-acting epigenetic event controlling *Bcl11b* activation timing in isolation from other events occurring in *trans*, we analyzed *Bcl11b* locus activation dynamics in progenitors that already have one *Bcl11b* allele active and must therefore contain all *trans*-factors necessary for expression (Figure 1B). Using fluorescence-activated cell sorting (FACS), we purified monoallelic *Bcl11b* expressing DN2 progenitors from dual-allelic reporter mice and analyzed activation of the silent allele by co-culture with OP9-DL1 cells, an *in vitro* system that recapitulates early transitions in T cell development (Holmes and Zuniga-Pflucker, 2009). Inactive *Bcl11b* alleles turned on after a long time delay such that the fraction of biallelically expressing cells increased progressively over the course of 5 days (Figures 1C and 1D). Activation kinetics were similar for both YFP (mCitrine yellow fluorescent protein) and RFP (mCherry red fluorescent protein) alleles and were well described by a single exponential curve, consistent with activation being controlled by a single stochastic event occurring with equal likelihood at each allele.

### H3K27me3 levels at the *Bcl11b* locus tune activation timing

**Figure 2.**
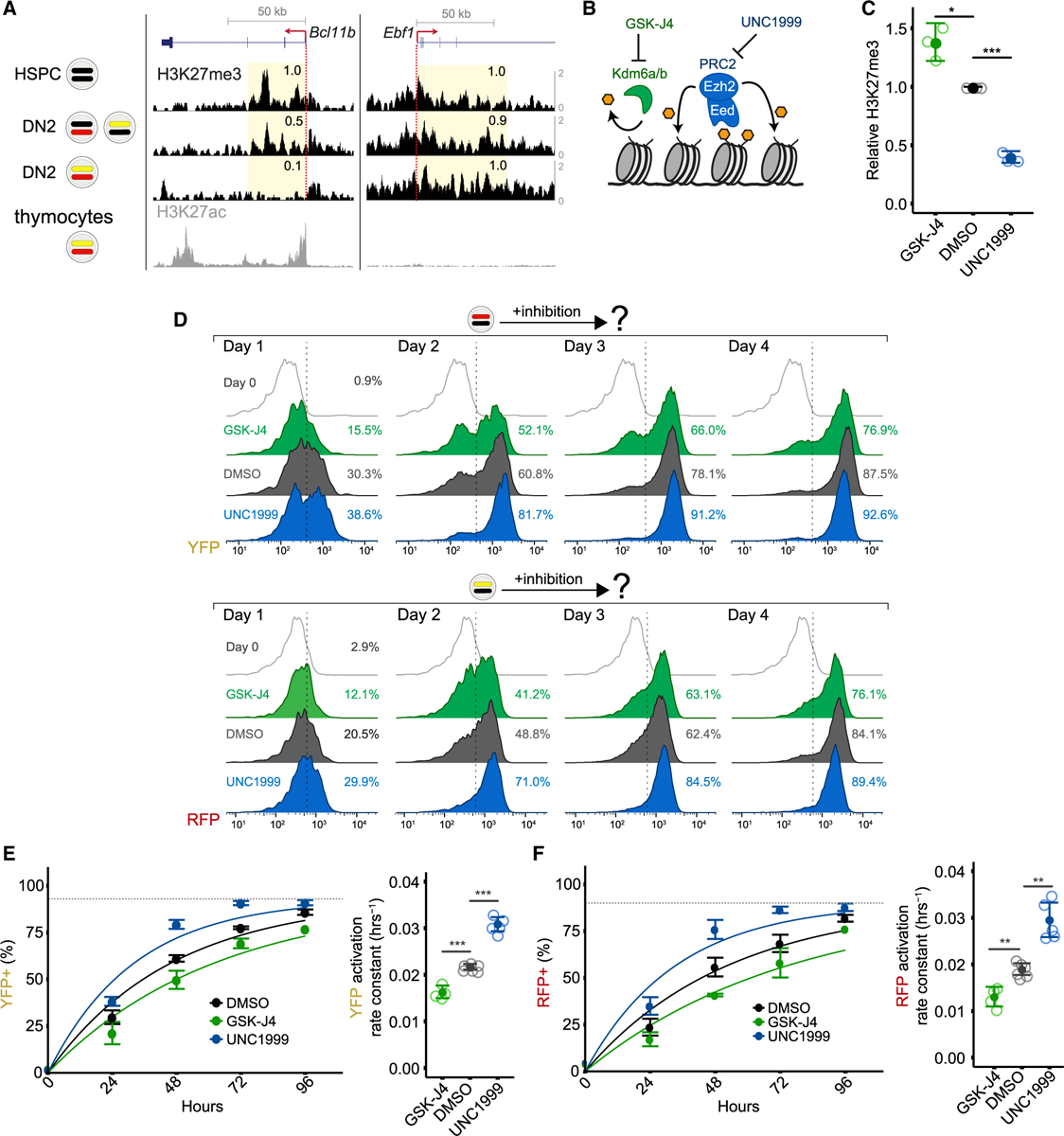
H3K27me3 levels, set by PRC2 and Kdm6a/b demethylases, modulate *Bcl11b* activation timing (A) H3K27me3 distributions were profiled by CUT&RUN in Lin− bone marrow progenitors (hematopoietic stem and progenitor cells [HSPCs]), as well as purified DN2 monoallelic and biallelic *Bcl11b*-expressing cells with UCSC Genome Browser tracks showing H3K27me3 densities at *Bcl11b*, as well as at *Ebf1*, a B cell regulator that is repressed during T cell development. Relative read densities of shaded areas are shown. H3K27ac levels in thymocytes, obtained from ENCODE accession number ENCSR000CCH (Davis et al., 2018), demarcate transcribed region. Data are representative of two independent experiments. (B) Schematic depicting inhibition of H3K27 demethylases Kdm6a/b or H3K27 methyltransferase PRC2. (C) DN2 monoallelic progenitors treated with the indicated inhibitors were sorted for anti-H3K27me3 CUT&RUN followed by qPCR at the *Bcl11b* promoter. Mean values are shown for n = 3 independent experiments (two-sample t test, one-tailed: *p < 0.05; ***p < 0.001). (D) Purified DN2 monoallelic expressing cells were re-cultured with the indicated inhibitors and analyzed by flow cytometry. Histograms show results from one representative experiment. (E and F) (Left) Mean activation percentages and 95% confidence intervals are plotted with curves representing fits to the equation y = *F*(1 − e^−kt^), where *F* = maximum percentage of cells positive for assayed allele (represented by the dotted gray lines). (Right) Data represent mean rate constants, *k*, with 95% confidence intervals (two-sample t test, one-tailed: **p < 0.01; ***p < 0.001; n = 4–6 independent experiments). See also Figure S1.

The repressive histone modification H3K27me3 is highly enriched at silent *Bcl11b* loci in hematopoietic progenitor cells, but not in committed T cells where *Bcl11b* is expressed (Zhang et al., 2012). Therefore, H3K27me3 removal could regulate the epigenetic event controlling *Bcl11b* activation timing. To test this possibility, we first determined whether H3K27me3 marks are removed from the *Bcl11b* locus at the same time it turns on. To pinpoint when H3K27me3 loss occurs relative to locus activation, we measured H3K27me3 levels in three progenitor populations having different numbers of active *Bcl11b* loci. In bone marrow progenitors, where both *Bcl11b* alleles are inactive, there was an abundance of H3K27me3 across the 5′ end of *Bcl11b* (Figure 2A). These broad H3K27me3 peaks were roughly halved in monoallelic *Bcl11b* expressing DN2 progenitors and were almost completely absent in biallelic *Bcl11b* DN2 progenitors (Figure 2A). These results show H3K27me3 is lost from the *Bcl11b* locus concurrently with its activation.

H3K27me3 may modulate the timing of *Bcl11b* activation; alternatively, its loss may simply be a consequence of gene activation due to clearance of methylated nucleosomes by active transcription (Hosogane et al., 2016; Kraushaar et al., 2013). To determine whether H3K27me3 modifications play a causal role in controlling *Bcl11b* activation timing, we cultured monoallelic *Bcl11b* expressing DN2 progenitors with small-molecule inhibitors targeting H3K27me3-modifying enzymes and analyzed the effects on activation of the silent *Bcl11b* allele. These inhibitors, which target either the PRC2 methyltransferase subunit Ezh2 (UNC1999) or the H3K27 demethylases Kdm6a/b (GSKJ4; Figure 2B), resulted in an ~60% decrease and ~40% increase, respectively, in H3K27me3 abundance at the *Bcl11b* promoter in monoallelic *Bcl11b* expressing DN2 progenitors (Figure 2C), indicating that they actively modulate H3K27me3 levels at inactive *Bcl11b* loci.

To determine whether H3K27me3 levels regulate *Bcl11b* activation timing, we assayed the expression of inactive *Bcl11b* alleles in DN2 progenitors over the course of 4 days. In the absence of any inhibitors, the silent *Bcl11b* alleles in monoallelic progenitors activated at an average rate of 0.022 h^−1^ and 0.019 h^−1^ for YFP and RFP alleles, respectively (Figures 2D–2F). Kdm6a/b demethylase inhibition decreased the activation rate for each silent allele in a graded manner (27%–37%). Conversely, Ezh2 inhibition increased the activation rate for each silent allele (41%–58%). Similar graded decreases or increases in *Bcl11b* activation probabilities were observed for another structurally unrelated Kdm6a/b inhibitor, IOX-1, and other Ezh2 inhibitors, GSK126 and GSK-343 (Figure S1A). Furthermore, short-hairpin-RNA-mediated knockdown of another essential PRC2 subunit, *Eed*, also increased the activation rate of silent *Bcl11b* alleles (Figures S1A, S1C, and S1D), arguing against non-specific pharmacological effects. Notably, all H3K27me3 perturbations tested altered the fraction of activated cells without altering expression levels in activated cells (Figure S1A), indicating a specific role of these modifications in tuning gene activation timing. Importantly, perturbing H3K27 methylation rates had no effect on the apoptosis frequency among different populations, indicating that these changes in activation fractions are not due to differential cell death in these populations (Figure S1E). Taken together, these results show that H3K27me3 levels at the *Bcl11b* locus, set by opposing PRC2 methyltransferase and Kdm6a/b demethylase activities, control the timing of *Bcl11b* activation.

### *Bcl11b* activation timing is regulated independently of cell division

**Figure 3.**
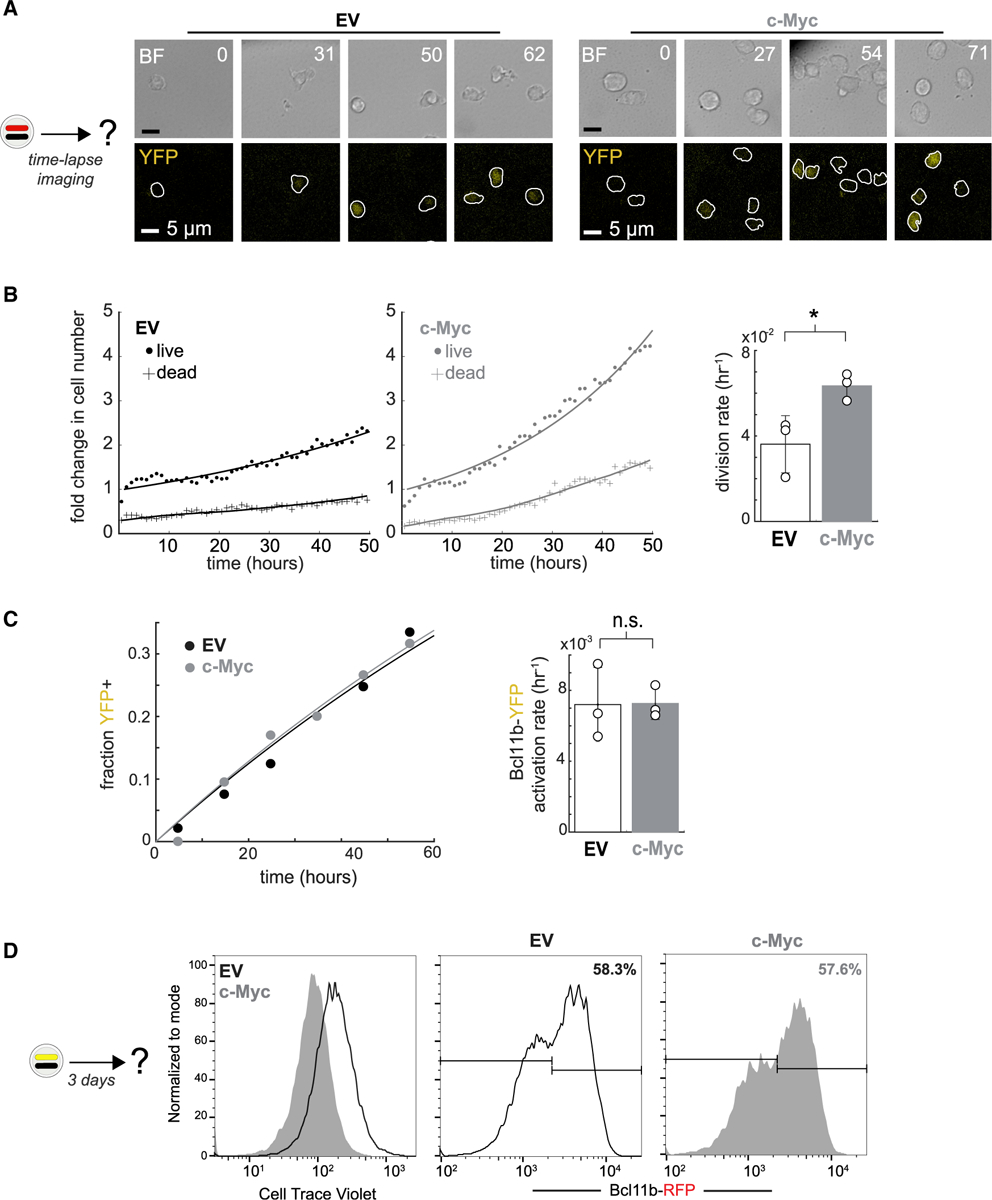
*Bcl11b* activation timing is independent of cell-division speed Bcl11b^YFP−/RFP+^ DN2 progenitors transduced with either an empty vector (EV) or c-Myc overexpressing retroviral vector were purified, re-cultured on DL1-coated plates, and monitored by time-lapse imaging. (A) Time-lapse images. White boundaries show automated cell segmentation. Numbers show elapsed time in hours. (B) (Left) Time evolution of live and dead cell numbers. Data were fitted to a population dynamics model as shown in Figure S2. (Right) Data represent mean and standard deviation of cell division rates for n = 3 independent experiments (paired two-sample t test, one-tailed; *p < 0.025). (C) (Left) Fraction of YFP+ cells over time. (Right) Data represent mean and standard deviation of *Bcl11b-YFP* activation rates for n = 3 independent experiments (paired two-sample t test, one-tailed; n.s., not significant). (D) Bcl11b^RFP+/YFP−^ DN2 progenitors transduced with either EV or c-Myc were re-cultured on OP9-DL1 stromal monolayers for 3 days before analyzing by flow cytometry. See also Figure S2 and Table S1.

Activation delays of polycomb-repressed genes have been proposed to result from the passive dilution of H3K27me3 modified histones with cell division (Coleman and Struhl, 2017; Jiang and Berger, 2017; Strome et al., 2014; Sun et al., 2014). However, the observed regulation of *Bcl11b* activation timing by H3K27 demethylases that counteract PRC2-mediated methylation (Figure 2) suggests an active mechanism is involved that could potentially operate independently of cell division. To determine whether *Bcl11b* activation timing depends on cell division, we accelerated the cell division rate in DN2 RFP+/YFP− progenitors by transducing them with the proto-oncogene c-Myc and used quantitative live-cell imaging to measure the activation kinetics of the silent YFP allele (Figure 3A). As expected, c-Myc overexpression resulted in an ~2-fold increase in the cell division rate (Figure 3B; see also Figure S2, Table S1, and Quantitative and statistical analysis). However, despite accelerating cell division, c-Myc overexpression did not change *Bcl11b* activation timing, with control and accelerated progenitors activating the silent YFP allele with the same exponential time constant (~136 h; Figure 3C). This lack of change in activation timing following cell division acceleration was also observed by flow cytometry for silent RFP alleles (Figure 3D). Therefore, in contrast to the passive dilution paradigm, these results show that the epigenetic switch controlling *Bcl11b* activation generates time delays in gene activation that are independent of cell division.

### A methylation-compaction mechanism for tunable, division-independent timing control

**Figure 4.**
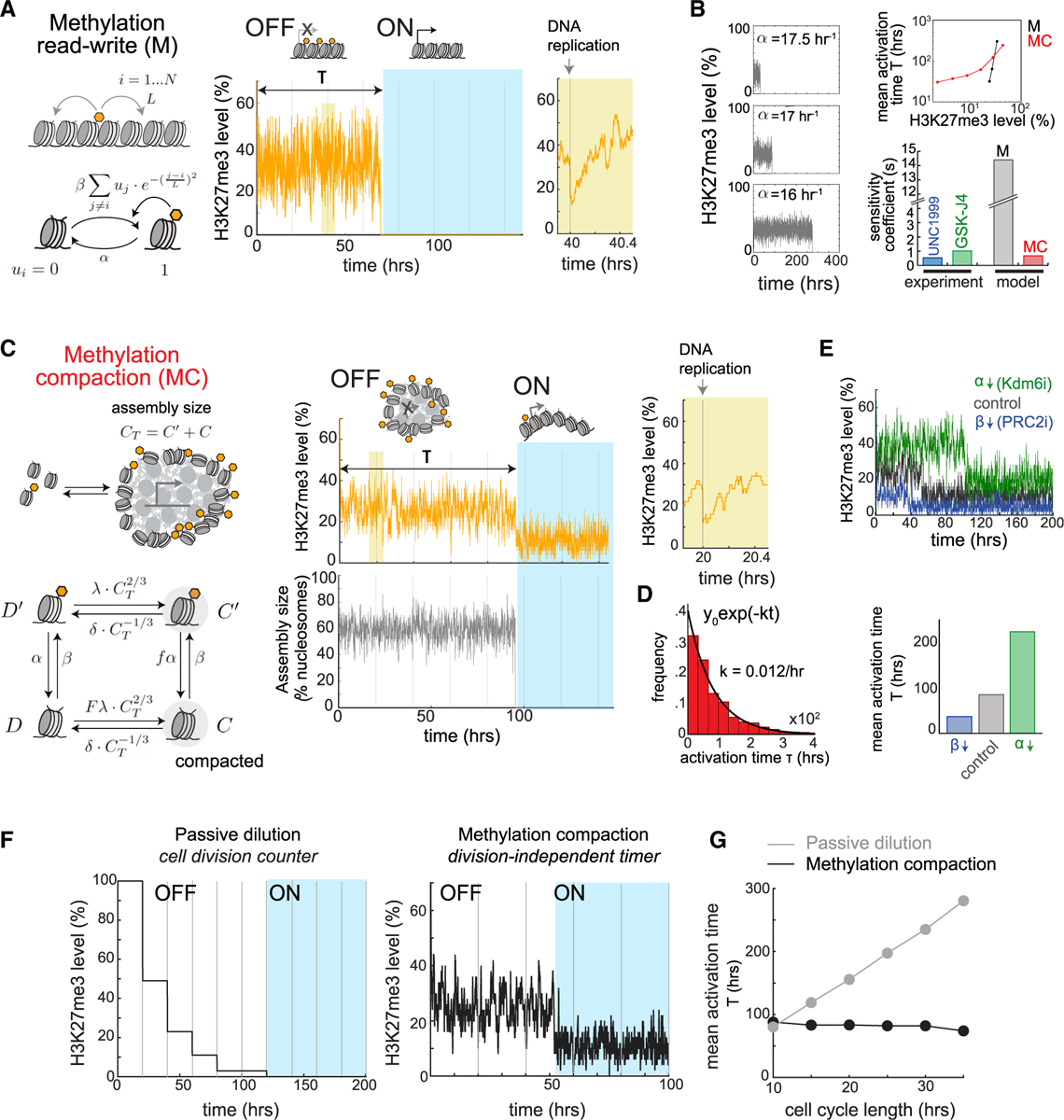
A methylation-compaction switching mechanism generates tunable, division-independent delays in gene activation (A) Methylation read-write (M) model (left), along with representative simulation (right). (B) Representative simulations of M model with different demethylation rates α (left). Mean activation times against H3K27me3 levels (top right) and sensitivity coefficients for this relationship (bottom right) are shown. (C) Methylation compaction (MC) model (left), along with representative simulation (right). (D) Histogram shows distribution of activation times, along with exponential fit. (E) Representative simulations of MC model, simulating PRC2 or Kdm6a/b inhibition (top), along with mean activation times (bottom). (F) Simulations of passive dilution and MC models. Vertical lines indicate DNA replication events. (G) Mean activation times as a function of cell cycle length. See also Figures S3–S7.

The molecular mechanism underlying the timed *Bcl11b* epigenetic switch must account for its observed emergent properties, namely, (1) its ability to robustly set time delays that span multiple cell generations, (2) its stochastic nature, (3) its tunability by histone-modifying enzyme activities, and (4) its cell division independence. To identify mechanisms with these emergent properties, we used mathematical modeling to analyze a series of candidate mechanisms. H3K27me3 can bind PRC2 at an allosteric site and activate its methyltransferase activity (Margueron et al., 2009), allowing these modifications to spread to neighboring nucleosomes and be maintained across cell division. Previous work has shown that such positive feedback, mediated by a read-write mechanism for histone methylation, can generate bistable switching that occurs over timescales spanning many cell generations (Dodd et al., 2007; Zhang et al., 2014). Therefore, we first analyzed a simple model of this positive feedback mechanism. In this methylation read-write (M) model, the *Bcl11b* locus comprises a linear array of *N* nucleosomes, each of which can be methylated or demethylated (Figure 4A; see Methods S1). Each nucleosome is methylated at a rate that increases with the number of nearby methylated nucleosomes and is demethylated at a first-order rate. During DNA replication, nucleosomes randomly segregate to the two daughter strands, resulting in half of the methylated nucleosomes being replaced by demethylated ones (Coleman and Struhl, 2017).

Our simulations revealed that single-nucleosome arrays could switch from a repressed H3K27 methylated state to a demethylated state with stochastic time delays spanning multiple cell divisions (Figure 4A, center), consistent with previous work (Dodd et al., 2007; Zhang et al., 2014). However, in our simulations, activation timing was extremely sensitive to H3K27 methylation levels in the silent state, with minor changes in methylation levels (~10%) causing dramatic changes activation timing (~300-fold; Figure 4B). This extreme sensitivity far exceeded the sensitivity coefficient observed experimentally (Figures 2C–2F and 4B) across a wide range of methyltransferase reach lengths (Figures S3A and S3B) and was also seen in other studies (Dodd et al., 2007; Zhang et al., 2014), indicating that it represents a general feature of positive-feedback-mediated switching and not a specific aspect of our model. By analyzing this system using a transition state theory framework (Figures S3C–S3E; see also Methods S1), we found that switching times scale exponentially with methylation or demethylation rates, thus explaining the observed extreme sensitivity. Thus, models that consider histone modification dynamics alone are inconsistent with the tunable control of activation by H3K27me3-modifying enzymes observed experimentally (Figures 2C–2F).

H3K27me3 modifications repress gene expression by promoting the formation of compacted chromatin assemblies inaccessible to the transcription machinery. Compaction may occur through H3K27me3-dependent recruitment of PRC1, which can self-associate through intrinsically disordered domains on its Cbx2 subunit to promote chromatin condensation (Plys et al., 2019; Tatavosian et al., 2019); alternatively, compaction may involve direct interactions between nucleosomes that are modulated by the H3K27me3 state (Gibson et al., 2019; Sanulli et al., 2019). In both cases, weak multivalent interactions between nucleosomes drive chromatin condensation through phase separation.

In light of these recent findings, we developed a second model, where H3K27me3 does not directly repress transcription per se but instead enhances the strength of nucleosomal interactions to keep the locus compacted and restrain its activation (Figure 4C; see Methods S1). In this methylation-compaction (MC) model, the *Bcl11b* locus consists of an ensemble of nucleosomes that can exist in methylated or demethylated states and can be included in or excluded from a compacted nucleosome assembly. Methylation and demethylation of nucleosomes occur with first-order rates, whereas association and dissociation of nucleosomes with the compacted chromatin assembly occur at rates proportional to its surface area, which we assume scales with the two-thirds power of the number of nucleosomes in the assembly *C*_*T*_. This rate dependency assumes that nucleosomes enter (or exit) the compacted assembly only by formation (or breakage) of weak multivalent interactions with nucleosomes on the assembly surface. Methylation and compaction are coupled such that the methylation state of a nucleosome affects its rate of association with a compacted assembly and vice versa. Below a threshold number of nucleosomes, the chromatin assembly is unstable and dissolves, leading to locus decompaction and consequent gene expression. This stability threshold reflects a minimum nucleus size needed to maintain a phase-separated condensate.

Simulations of the methylation-compaction model revealed that the gene locus can maintain a H3K27 methylated and compacted state for multiple cell divisions before switching in an all-or-none manner to a decompacted, low-methylation state (Figure 4C, center). As with the methylation-only model, the time delay in switching is well described by a first-order stochastic process, with a constant probability of activation per unit time (Figure 4D). However, in contrast with the methylation read-write model but in concordance with our experimental results (Figure 2), changing H3K27me3 levels by varying methylation or demethylation rates changed gene activation timing in a much more graded manner (Figures 4B and 4E). This tunability was robust over different parameter ranges (Figure 4B, top right), different degrees of cooperativity for H3K27 methylation (Figure S4), and different degrees of assembly disruption after DNA replication (Figures S5A–S5E). A transition state theory analysis (see Methods S1 and Figures S3C–S3E) showed that, in order for gene activation timing to be finely tunable, nucleosomes must be able to associate with each other even without H3K27me3 modifications, such that methylated and demethylated nucleosomes can associate with each other with comparable affinities. Consistent with this idea, there are multiple mechanisms for nucleosomal interactions that work independently of H3K27me3 modifications (Francis et al., 2004; Gibson et al., 2019; Larson et al., 2017; Sanulli et al., 2019; Strom et al., 2017).

As cell division rate did not affect timing delays in *Bcl11b* activation (Figure 3), we tested whether the methylation-compaction mechanism also generates cell-division-independent activation delays. Indeed, in contrast to a passive dilution model for H3K27me3 loss (Figure 4F), the methylation-compaction model generated activation delays that were constant over a range of cell cycle speeds (Figures 4G and S5A–S5E). We note that this cell division independence was lost when histone methylation and demethylation rates were reduced to be slower than the cell division rates (Figure S5F), implying a need for rapid histone methylation dynamics for upholding cell cycle independence. Indeed, histone methylation and nucleosome compaction dynamics occur with reported timescales of minutes and seconds (Kristensen et al., 2011; Larson et al., 2017; Sneeringer et al., 2010), respectively, which are far faster than the typical cell division rates which range from hours to days. This explains why the epigenetic state recovers rapidly after DNA replication as observed in our simulations (Figures 4A and 4B, right).

These modeling results suggest that timing control by H3K27me3 has the following characteristics: first, H3K27me3 loss does not directly result in gene activation but instead modulates a separate process that acts as the gatekeeper for gene transcription. Given recent insights into how nucleosomes can interact to form phase-separated structures (Gibson et al., 2019), and how these interactions can be modulated by histone tail modifications and/or binding proteins (Larson et al., 2017; Plys et al., 2019; Strom et al., 2017; Tatavosian et al., 2019), we propose that H3K27me3 loss weakens nucleosomal interactions at the *Bcl11b* locus, promoting gene locus decompaction and gene activation. Second, the compaction process itself must be partially independent of H3K27me3 such that the compacted nucleosome assembly can be maintained by other chromatin-associated proteins (Francis et al., 2004; Larson et al., 2017; Strome et al., 2014).

### The *Bcl11b* locus switches to an extended conformation with activation

**Figure 5.**
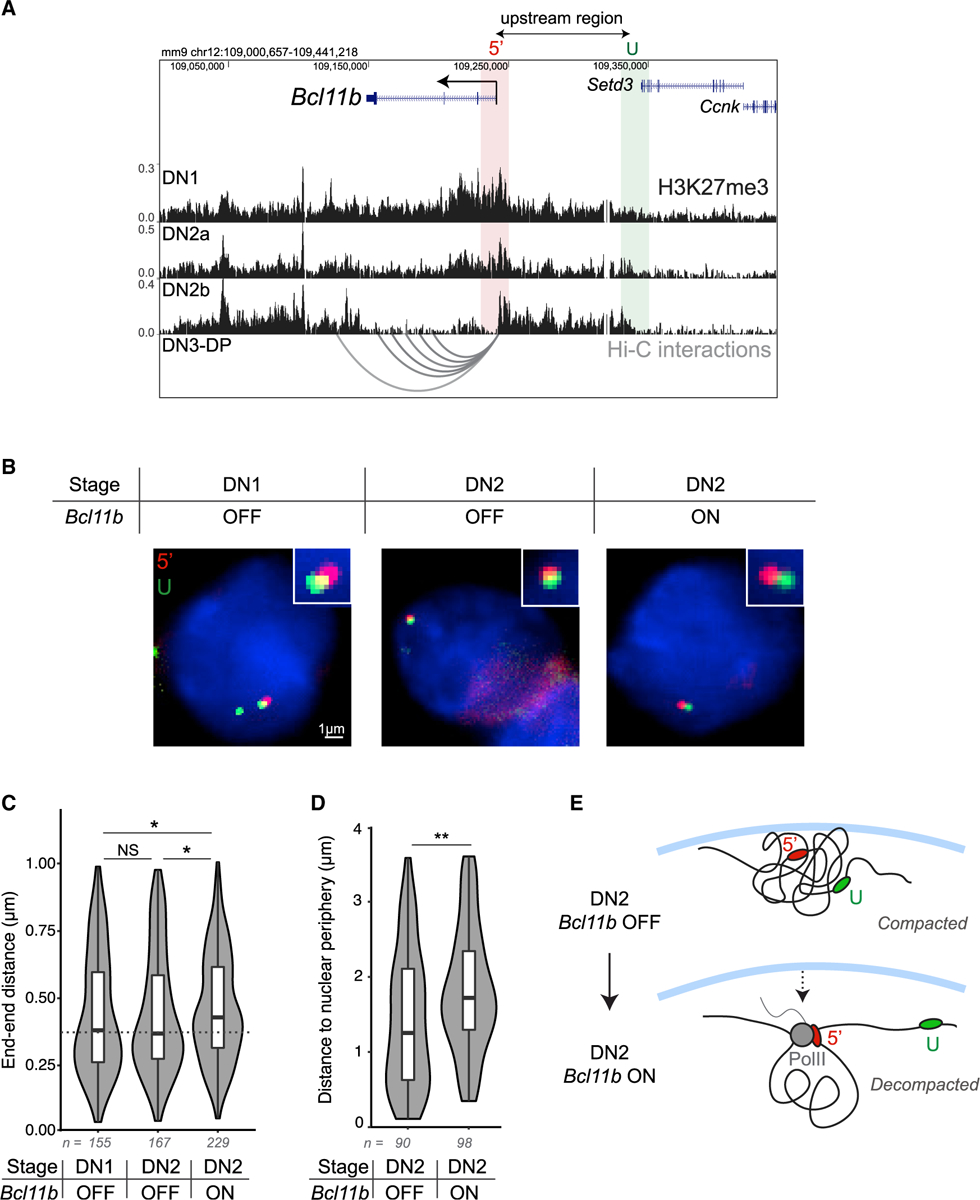
The *Bcl11b* locus switches to an extended conformation with activation (A) UCSC Genome Browser view of H3K27me3 chromatin immunoprecipitation sequencing (ChIP-seq) results in T cell progenitors (Zhang et al., 2012) and Hi-C representation maps of interactions between the *Bcl11b* transcription start site and other DNaseI hypersensitivity sites in Bcl11b+ DN3-DP T cell progenitors (Hu et al., 2018). (B) Representative images for each condition. Centroids for each foci pair shown exist in the same *z*-plane and thus provide visual representation of the Euclidean distance. (C) Violin plots show the results from 3D Euclidean distance measurements between each probe pair (Mann Whitney U test significance: *p < 0.05; n = number of foci pairs). T cell progenitors were sorted based on cell surface markers and *Bcl11b* reporter expression (either *Bcl11b* OFF [RFP−/YFP−] or *Bcl11b* ON [RFP+/YFP+]) before performing DNA-FISH with upstream end-end probes above. (D) Violin plots show the results from 2D measurements between the *Bcl11b* promoter and the nuclear periphery (Mann Whitney U test significance: **p < 0.01; n = number of foci). (E) Schematic depicting decompaction model. In the OFF state, *Bcl11b* exists in a compacted conformation residing at the nuclear lamina. In the ON state, the locus moves away from the nuclear periphery and becomes decompacted, resulting in increased distance between the promoter, 5′, and the upstream, U, regions.

The methylation-compaction mechanism above assumes that the *Bcl11b* locus is compacted prior to activation but switches to an extended, decompacted state during activation. To validate this assumption, we measured the end-to-end distances between genomic regions at the *Bcl11b* locus using DNA fluorescence *in situ* hybridization (FISH), an established approach to estimate the degree of chromatin compaction at a gene locus (Eskeland et al., 2010; Giorgetti et al., 2015). To estimate the degree of compaction independently from RNA polymerase II (RNAPII)-mediated DNA looping at the *Bcl11b* locus (Hu et al., 2018; Zheng et al., 2019), we designed a pair of FISH probe sets that flank a 100-kb region upstream of the promoter. This upstream region begins at the *Bcl11b* promoter and resides at the edge of the putative heterochromatin compaction domain that encompasses *Bcl11b* (Figure 5A). We performed DNA-FISH in early T cell progenitors (DN1) and DN2 progenitors before or after *Bcl11b* activation (RFP−/YFP− versus RFP+/YFP+) and measured end-to-end distances using three-dimensional imaging.

These experiments showed that the *Bcl11b* locus showed similar end-to-end distances in RFP−/YFP− DN1 and DN2 progenitors; however, in RFP+/YFP+ DN2 progenitors, where *Bcl11b* first turns on, this end-to-end distance increased significantly, consistent with this locus maintaining a condensed state through early T cell development but switching abruptly to an extended state during *Bcl11b* activation (Figures 5B and 5C). Repressed heterochromatic regions of the genome frequently reside at the nuclear periphery, where interactions between nuclear lamina proteins and nucleosomes are thought to facilitate chromatin compaction (van Steensel and Belmont, 2017; Ulianov et al., 2019). Therefore, we examined whether *Bcl11b* moves from the nuclear periphery to the interior as it decompacts and turns on, as suggested from previous studies (Isoda et al., 2017). Indeed, the distance between the *Bcl11b* promoter and the nuclear periphery was higher in *Bc111b*-expressing DN2 progenitors compared to *Bcl11b*-non-expressing progenitors at the same DN2 stage, consistent with a transition from the nuclear periphery to the interior upon gene activation (Figure 5D). This finding, in conjunction with the observed increase in the end-end distance of the *Bcl11b* locus, suggests that *Bcl11b* transitions from a condensed heterochromatin-associated state to an accessible euchromatin-associated state during transcriptional activation (Figure 5E), consistent with the methylation-compaction model.

### H3K27me3-independent regulation of *Bcl11b* epigenetic switch timing

The methylation-compaction model predicts that, in order for activation time delays to be tunable, nucleosomes must retain an ability to interact independently of H3K27me3. H3K9me2/3 is also associated with repressive heterochromatin domains and can serve as a binding site for HP1α, which can facilitate nucleosome adhesion in the absence of H3K27me3 (Poleshko et al., 2013; Sanulli et al., 2019; Wang et al., 2019). H3K9me3 is enriched at the *Bcl11b* locus in non-T-cell lineages (Figure S6A); therefore, we tested whether H3K9 methylation also regulates *Bcl11b* activation timing. To do so, we sorted DN2 progenitors with one active *Bcl11b* allele, re-cultured them with inhibitors targeting H3K9me3-modifying enzymes, and quantified the *Bcl11b* activation state after 3 days. We found that inhibition of Lsd1, an H3K9 demethylase, decreased the fraction of biallelic *Bcl11b*-expressing cells, whereas inhibition of G9a, an H3K9 methyltransferase, increased the fraction of biallelic *Bcl11b*-expressing cells (Figures S6B and S6C). The dose-dependent yet moderate degree to which H3K9 methylation perturbations altered *Bcl11b* activation rate was similar to that for H3K27 methylation perturbations, as seen in Figure 2D. Thus, consistent with the methylation-compaction model, multiple histone modifications work together to modulate *Bcl11b* activation timing, conceivably by working together to modulate nucleosomal interactions at the gene locus.

### Transcription factors can tune gene activation time delays

*Bcl11b* activation timing is tunable not only by chromatin-modifying enzymes, as shown above (Figure 2), but also by two transcription factors, Gata3 and TCF-1, through a distal enhancer that physically interacts with the *Bcl11b* promoter before activation (Hu et al., 2018; Isoda et al., 2017; Kueh et al., 2016; Ng et al., 2018). These findings are consistent with broader literature showing that transcription factors and their binding sequences can modulate target gene activation probabilities (Dufourt et al., 2018; Walters et al., 1995; Weintraub, 1988), though it remains unknown how they achieve such tunable timing control. Here, we tested whether the methylation-compaction mechanism could integrate information about transcription factor levels to control activation timing. We first considered a scheme, where transcription factors bind to nucleosomes and block their association with other nucleosomes in the compacted assembly (Figure S7A). Indeed, both Gata3 and TCF-1 have been identified as pioneer factors, which possess affinity to nucleosomes in addition to specific DNA sequences (Fernandez Garcia et al., 2019; Johnson et al., 2018; Meers et al., 2019; Zaret and Carroll, 2011). Such disruption of compaction could also occur via the activation of gene or non-coding RNA transcription (Rinn et al., 2007; Tu et al., 2017) or by recruitment of factors that disrupt interactions between nucleosomes (Kraushaar et al., 2013; Talbert and Henikoff, 2017; Zhou et al., 2016).

Our simulations revealed that the rate constant for gene activation varied with both transcription factor concentration and the number of transcription factor binding sites (Figure S7B). Increasing the number of bound nucleosomes *N*_*B*_ increased the activation rate in a synergistic manner, with a progressive increase in the maximal activation rate with addition of each binding site (Figure S7B, top right). Interestingly, increasing the number of binding sites also increased the transcription factor concentration at which half-maximal activation occurred (Figure S7B, bottom right). These findings suggest that transcription factors can tunably control the timing of gene activation by blocking internucleosomal interactions and chromatin compaction.

Besides blocking nucleosomal interactions, transcription factors may also promote activation by inducing histone demethylation. Histone demethylation could occur by direct recruitment of Kdm6a/b demethylases (Estarás et al., 2013; Seenundun et al., 2010; Williams et al., 2014) or through PRC2 eviction by chromatin-remodeling enzymes (Kadoch et al., 2017). Therefore, we considered a second mechanism, where transcription factors induce the demethylation of *N*_*R*_ nucleosomes around their binding vicinities (Figure S7C). Our simulations revealed that transcription factors could modulate activation timing, as above, but only if they could induce demethylation of a large number of nucleosomes in their binding vicinity (Figure S7D). When a smaller number of nucleosomes were impacted, activation rates shifted only slightly when transcription factor levels increased (Figure S7D). Consistent with this idea, transcription factors bound to a single binding site can often induce chromatin changes over a significant area spanning many nucleosomes (Hass et al., 2015; Heinz et al., 2010).

## DISCUSSION

During development, progenitor cells differentiate, with time delays spanning multiple days and cell generations. These delays are often independent of cell division and are tunably controlled by regulatory inputs. Here, we elucidated the mechanism of a time-delayed epigenetic switch controlling the activation of the T cell specifying gene *Bcl11b* and developed a mathematical model to explain its emergent properties. We show that H3K27me3 levels at the *Bcl11b* locus, set by opposing methyltransferase and demethylase activities, modulate the *Bcl11b* activation time delay by controlling its switch from a compacted, silent state to an extended, actively expressing state. Activation delays generated by this methylation-compaction mechanism robustly span multiple cell generations, can be tunably modulated by both histone-modifying enzymes and transcription factors, and are set independently from cell division.

In contrast to previous epigenetic switching models, which only consider the dynamics of histone modification (Dodd et al., 2007; Zhang et al., 2014), the methylation-compaction model we propose couples histone methylation to nucleosomal interactions. Consistent with this idea, both H3K27me3 and H3K9me2/3 can promote nucleosomal self-association though histone-tail interactions (Gibson et al., 2019) or by recruiting protein complexes that self-associate to form phase-separated condensates (Plys et al., 2018; Sanulli et al., 2019; Tatavosian et al., 2019; Wang et al., 2019). Importantly, in order for the activation times to be tunable, nucleosomes must retain some self-interaction affinity without H3K27me3, such that methylation promotes but is not strictly necessary for nucleosomal association. The concept that modification states of proteins modulate their interaction affinities is well established in the study of cytoskeletal polymers (Howard, 2001; Mitchison, 1992; Phillips et al., 2012) but could provide a fresh perspective on the relationship between chromatin modifications and chromatin structure. Further testing of the methylation-compaction model will require direct interrogation of chromatin states at individual gene loci in single cells, work that will be aided by new methods to simultaneously visualize histone modification states and chromatin folding at single gene loci in single cells (Kundu et al., 2017; Xu et al., 2018; Woodworth et al., 2020).

The methylation-compaction switching mechanism could underlie diverse cell-autonomous timers that have been observed to work independently of cell division (Burton et al., 1999; Gao et al., 1997; Heinzel et al., 2017; Li et al., 2019; Okamoto et al., 2016; Osmond, 1991; Otani et al., 2016). Measuring elapsed time independently of cell division could enable unique functions, including operation in non-dividing cells and constancy amid changes to cell proliferation, which could allow for tunable population size control, an idea we explore in a separate study (Nguyen et al., 2019). Our simulations revealed that such division-independent timing control requires active turnover of H3K27me3 and nucleosome compaction dynamics to be rapid compared to the cell cycle length (Methods S1; Figure S5F). We currently lack methods to measure H3K27me3 turnover kinetics at specific genomic loci *in vivo*; however, the active roles of PRC2 and Kdm6a/b demethylases in modulating H3K27me3 levels at the *Bcl11b* locus (Figure 2), their fast catalysis rates (Kristensen et al., 2011; Sneeringer et al., 2010), along with the observation that nucleosomes within polycomb domains are replaced with kinetics much faster than that of cell division (1.5 h versus 20 h; Deal et al., 2010) suggest that H3K27me3 indeed turns over at a much faster timescale than that of cell division.

Cell type specification during multicellular development is controlled by gene-regulatory networks whose dynamics unfold over timescales spanning many cell generations. The division-independent timed epigenetic switch we describe here is uniquely tunable at multiple levels of gene regulation, including histone modifications, transcription factors, and non-coding *cis*-regulatory elements. Thus, it could serve as a modular building block for gene-regulatory networks that enables robust, adjustable control of developmental timing as well as organism size and form.

## STAR★METHODS

### RESOURCE AVAILABILITY

#### Lead contact

Further information and requests for resources and reagents should be directed to and will be fulfilled by the Lead Contact, Hao Yuan Kueh (kueh@uw.edu).

#### Materials availability

The pMSCV-mTagBFP2-shEed plasmid is available from the Lead Contact upon request.

#### Data and code availability

The accession number for the H3K27me3 CUT&RUN data reported in this paper is NCBI Gene Expression Omnibus: GSE134749. Scripts used for imaging analysis and simulations of mathematical models have been deposited to GitHub: https://github.com/KuehLabUW/Pease_et_al.2021. All other data supporting the findings of this paper will be available from the Lead Contact.

### EXPERIMENTAL MODEL AND SUBJECT DETAILS

#### Animal Models

C57BL/6 *Bcl11b*^*RFP/YFP*^ mice were generated as described before (Ng et al., 2018). Briefly, *Bcl11b*^*YFP/YFP*^ mice were generated by inserting an IRES-H2B-mCitrine-neo cassette into the 3′ UTR of *Bcl11b* and *Bcl11b*^*RFP/RFP*^ mice were generated by inserting an IRES-H2B-mCherrry-neo cassette into the same location. Dual allelic *Bcl11b*^*RFP/YFP*^ mice with identical *Bcl11b* alleles except for fluorescent protein reporters were generated by breeding *Bcl11b*^*RFP/RFP*^ mice to *Bcl11b*^*YFP/YFP*^ mice. Bone marrow derived from F1 *Bcl11b*^*RFP/YFP*^ mice at 2–4 months of age were used for all *in vitro* T cell development assays. Sex was determined not to be influential for these studies, thus male and female bone marrow were combined and analyzed together. All animals were bred and maintained at the University of Washington. All animal protocols were reviewed and approved by the Institute Animal Care and Use Committee at the University of Washington (Protocol No: 4397-01).

#### Cell Line Culture

Primary cells isolated from bone marrow were cultured on a OP9-DL1 monolayer stromal cells (Holmes and Zuniga-Pflucker, 2009) at 37°C in 5% CO_2_ conditions with standard culture medium [80% aMEM (GIBCO), 20% Fetal Bovine Serum (Corning), Pen-Strep-Glutamine (GIBCO)] supplemented with appropriate cytokines (described in Method Details). Phoenix-Eco cells were cultured at 37°C in 5% CO_2_ with standard culture medium [90% DMEM (GIBCO), 10% Fetal Bovine Serum (Corning), Pen-Strep-Glutamine (GIBCO)] All cell lines were tested and found to be negative for mycoplasma contamination.

### METHOD DETAILS

#### Cell purification

To isolate hematopoietic stem and progenitor cells (HSPCs) for *in vitro* differentiation or CUT&RUN experiments, bone marrow cells were harvested from femurs and tibias of 2 to 4 month-old *Bcl11b*^*RFP/YFP*^ mice. CD117 MicroBeads (Miltenyi Biotec) were used to enrich HPSCs which were frozen in 90% FBS and 10% DMSO at 10^6^ cells/mL. For CUT&RUN experiments, HPSCs were further purified by staining with anti-CD117 APC-eFluor780 (ThermoFisher Scientific) and with biotinylated antibodies against a panel of bone marrow lineage markers (CD19, CD11b, CD11c, NK.1.1, Ter119, CD3ε, Gr-1 and B220 (BioLegend)). Cells were then washed with HBH (Hank Balanced Salt Solution (HBSS) with 0.1% bovine serum albumin and 10mM HEPES) and stained with streptavidin-PerCP/Cy5.5 (BioLegend).

#### *In vitro* differentiation of T cell progenitors

To generate double-negative (DN) T cells *in vitro*, thawed CD117-enriched bone marrow progenitors were cultured on OP9-DL1 stromal cell monolayers as described before using standard culture medium [80% αMEM (GIBCO), 20% Fetal Bovine Serum (Corning), Pen-Strep-Glutamine (GIBCO)], grown at 37°C in 5% CO2 conditions]. All *in vitro* T cell generation cultures were supplemented with 5ng/mL Flt3-L and 5 ng/mL IL-7 (Peprotech), and were sorted after 6 to 8 days of culture before transducing with retroviral vectors or treating with small molecule inhibitors. DN2 cells were re-cultured in the same conditions following all cell sorting experiments.

#### Flow cytometry and cell sorting

Fluorescence activated cell sorting (FACS) was used to isolate DN2 cells of interest with the following protocol. Bone marrow derived cell cultures were scraped and incubated in 2.4G2 Fc blocking solution and stained with anti-CD25 APC-eFluor 780 (Clone PC61.5, eBioscience) and with biotinylated antibodies against a panel of lineage markers (CD19, CD11b, CD11c, NK.1.1, Ter119, CD3ε, Gr-1 and B220 (BioLegend)). Stained cells were washed with HBH (Hank Balanced Salt Solution (HBSS) with 0.1% bovine serum albumin (BSA) and 10mM HEPES and stained with streptavidin-PerCP/Cy5.5 (BioLegend). Stained cells were washed, resuspended in HBH, and filtered through a 40-um nylon mesh for sorting with a BD FACS Aria III (BD Biosciences) with assistance from the University of Washington Pathology Flow Cytometry Core Facility. A benchtop MacsQuant VYB flow cytometer (Miltenyi Biotec) and a benchtop Attune NxT Flow Cytometer (ThermoFisher Scientific) were used to analyze time course and perturbation experiments and acquired data were analyzed with FlowJo software (Tree Star).

#### Retroviral construct and transduction

Overexpression of c-Myc was achieved using cMyc H2B-mCerulean MSCV retroviral vector which was described previously (Kueh et al., 2016). Retroviral mir30-based constructs (a gift from J. Zuber) were used as a backbone for delivering short hairpin RNA (Fellmann et al., 2013). pBAD-mTagBFP2 (a gift from V. Verkhusha, Addgene plasmid #34632) was used to substitute mTagBFP2 for the existing GFP using PCR cloning with the restriction enzymes NcoI and SalI. The pMSCV-mTagBFP2-shEed retroviral construct was generated by PCR cloning as previously described (Fellmann et al., 2013) using the mir-30-shEed PCR template sequence and pMSCV-mir-30 backbone described in the Key resources table.

Retroviral particles were generated using the Phoenix-Eco packaging cell line. Viral supernatants were collected at 2 and 3 days after transfection and immediately frozen at −80°C. To infect bone marrow derived T cell progenitors, 33 μg/mL retronectin (Clontech) and 2.67 μg/mL of DL1-extracellular domain fused to human IgG1 Fc protein (a gift from I. Bernstein) were added in a volume of 250 μL per well in 24-well tissue culture plates (Costar, Corning) and incubated overnight. Viral supernatants were added the next day into coated wells and centrifuged at 2000 r*cf*. for 2 hours at 32°C. Bone marrow derived derived T cell progenitors used for viral transduction were cultured for 6–7 days according to conditions described above, disaggregated, filtered through a 40-μm nylon mesh, and 10^6^ cells were transferred onto each retronectin/DL1-coated virus-bound well supplemented with 5 ng/mL SCF (Peprotech), 5 ng/mL Flt3-L, and 5 ng/mL IL-7.

#### CUT&RUN H3K27me3 profiling

CUT&RUN experiments were carried out as previously described (Skene et al., 2018) with the following modifications: 1–2.5×10^5^ cells were isolated by FACS as described in sections above, bound to Concanavalin A coated magnetic beads (Bangs Laboratories), and permeabilized with 0.025% (wt/vol) digitonin. Permeabilized cells were incubated overnight at 4°C with 5ug of anti-H3K27me3 (Active Motif) and then washed before incubating with protein A-MNase fusion protein (a gift from S. Henikoff) for 15 minutes at room temperature. After washing, cells were incubated in CaCl_2_ to induce MNase cleavage activity for 30 minutes at 0°C. The reaction was stopped with 2xSTOP buffer (200 mM NaCl, 20 mM EDTA, 4 mM EGTA, 50 μg/mL RNase A, 50 μg/mL glycogen, and 2pg/mL of yeast spike-in DNA). Histone-DNA complexes were isolated from insoluble nuclear chromatin by centrifugation and DNA was extracted with a NucleoSpin PCR Clean-up kit (Macherey-Nagel). For CUT&RUN quantitative PCR, human Kasumi-1 cell line (ATCC CRL-2724) were added before binding the cells to Concanavalin A beads for internal standard instead of yeast spike-in DNA.

#### CUT&RUN library preparation and sequencing

Library preparation from CUT&RUN products was completed with KAPA Hyper Prep Kit (KAPA Biosystems) following standard protocol with PCR amplification settings adjusted so that annealing and extension steps are combined into one step at 60°C for 10 s. Library products were size selected to be within 200 – 300 bp range using AMPure beads (Agencourt). Libraries were sequenced using an Illumina MiSeq system with paired-end 25 bp sequencing read length and TruSeq primer standard for approximately 5 millions reads per sample.

#### CUT&RUN sequencing analysis

Paired-end sequencing reads were aligned separately to mouse (NCBI37/mm9) and yeast (SacCer_Apr2011/sacCer3) genomes using Bowtie2 (Langmead and Salzberg, 2012) with the following setting:–local–very-sensitive-local–no-unal–no-mixed–no-discordant -I 10 -X 700 as suggested for mapping CUT&RUN sequencing data (Skene et al., 2018). The alignment setting was designed to specifically search with high stringency for only appropriately paired reads with the proper orientation. The resulting alignments were converted to BAM files with SAMtools (Li et al., 2009) and then converted to BED files with BEDTools (Quinlan and Hall, 2010). Reads were sorted and filtered to remove random chromosomes. BEDTools genomecov was used to generate histograms for the mapped reads using a scaling factor that is the product of the number of spiked-in yeast reads and the number of input cells. The resulting bedGraph files were visualized using the UCSC Genome Browser (Davis et al., 2018; Kent et al., 2002).

#### CUT&RUN qPCR

Extracted DNA from CUT&RUN samples was size selected with Ampure XP magnetic beads (Beckman Coulter) to remove fragments > 800bp. Primers were designed to detect the mouse *Bcl11b* promoter (see Key resources table for sequences). PowerUp SYBR Green Master Mix (ThermoFisher Scientific) and CFX96 Real-Time PCR Detection System (Bio-Rad) were used for quantitative PCR. Since Kasumi-1 cells were used as internal standard, relative enrichment of H3K27me3 at *Bcl11b* was quantified by the ΔΔCq method using the human *PAX5* promoter for normalization to account for differences in efficiency and sample loss during processing.

#### Cell preparation for time-lapse imaging

T cell progenitors derived from the *in vitro* differentiation protocol above were harvested and infected with either a MSCV empty vector or c-Myc overexpression vector harboring an IRES-H2B-mCerulean reporter cassette. 16–24 hours later CFP-positive cells were purified by FACS and seeded onto PDMS micromesh (250 μm hole diameter, Microsurfaces) mounted on top of a 24-well glass bottom plate (Mattek). To prepare the stromal-free differentiation system, which facilitates cell identification during imaging, the top face of PDMS micromesh was first blocked by incubating in solution of 130 μg/ml BSA while mounted on top of a 24-well plate overnight at 4°C. This step prevents subsequent binding of retronectin to the side of the micromesh walls. Blocked micromeshes was then transferred to a clean 24-well glass bottom plate. The well and mesh constructs were incubated in a solution of 10 μg/ml retronection and 3 μg/ml DL-1 overnight at 4°C. The well was then washed with PBS, and culture media [80% αMEM (GIBCO), 20% Fetal Bovine Serum (Corning), Pen-Strep-Glutamine (GIBCO), 5 ng/ml IL-7 (Clontech), 5 ng/ml Flt-3 (Clontech), 50 ng/ml mSCF (Clontech), 50 μM beta-mercaptoethanol (Sigma) grown at 37°C in 5% CO2 conditions] was added, and sorted cells were introduced at a concentration of 5–10 cells per microwell. This stromal-free system enables a greater fold enhancement of cell division rate by cMyc transduction and better resolution for imaging as well as recapitulating Bcl11b activation and T cell lineage commitment, but supports a lower baseline rate of proliferation in unmodified cells compared to the OP9-DL1 system.

#### Oligopaint DNA-FISH

The OligoMiner pipeline was used to design Oligopaint libraries (Beliveau et al., 2018). 35–52bp probes were designed to target 20kb regions at a density of approximately 12–14 probes per kilobase. Bone marrow progenitors were grown on OP9-DL1 stromal cells for 8 days under normal growth conditions. Cells were filtered through a 70uM filter and incubated with 2.4G2 blocking buffer before staining with anti-CD25 APC-eFluor 780 (Clone PC61.5, eBioscience), anti-CD44 APC (Clone IM7, eBioscience), and biotinylated antibodies against a panel of lineage markers (CD19, CD11b, CD11c, NK.1.1, Ter119, CD3ε, Gr-1 and B220 (BioLegend)). DN1 progenitors (CD25^−^/CD44^+^/Bcl11b^RFP−/YFP−^), DN2a progenitors (CD25^+^/CD44^+^/Bcl11b^RFP−/YFP−^) and DN2b progenitors (CD25^+^/CD44^+^/Bcl11b^RFP+/YFP+^) were purified by FACS and centrifuged on top of poly-L-lysine coated 18-well chambered glass coverslips (Ibidi). Cells were then fixed with 4% paraformaldehyde for 10 minutes and permeabilized for 10 minutes in 0.1% Triton X-100 before performing the Oligopaint DNA-FISH protocol (Beliveau et al., 2017). Permeabilized cells were incubated in 0.1N HCl for 5 minutes, followed by RNaseA (100ug/ml) for 1 hour at 37°C. Cells were then washed with 2x SSCT (2x saline sodium citrate + 0.1% Tween-20) and incubated with 2x SSCT + 50% formamide for 20 minutes at 60°C. A hybridization mixture was prepared containing 50% formamide, 2x SSCT, 3mM sodium azide, 10% dextran sulfate, 100nM of adaptor oligos, 100nM of fluorescently labeled reporter oligos, and 500nM of probes. The hybridization mixture was added and incubated for 3 minutes at 78°C before incubating overnight in a humidifier chamber at 37°C. Approximately 18 hours later, cells were washed with pre-heated 2x SSCT for 5 minutes at 60°C. This step was repeated four times before performing the final wash at room temperature. Cells were then stained with 10ug/mL Hoechst 33342 (ThermoFisher) for 15 minutes before washing with PBS and imaging in with a photoprotective buffer (10% glucose, 200mM Tris, glucose oxidase (GLOX), catalase, 1mM methyl viologen hydrate, 1mM ascorbic acid).

#### FISH imaging and analysis

Cells were imaged with an inverted widefield fluorescence microscope (Leica DMi8) using a 100X oil objective, using an sCMOS camera (Photometrics Prime 95B) and a motorized stage (ASI MS-2000). Z sections were collected at a step size of 100nm. Chromatic aberrations were corrected for using Fiji (Schindelin et al., 2012) and BUnwarpJ (Arganda-Carreras et al., 2006) as described previously (Giorgetti et al., 2015). After nuclei segmentation, the *z* slice with the maximum intensity for each foci was chosen as the *z* coordinate. Each selected *z* slice was then fit to a two-dimensional Gaussian to determine the *xy* coordinates for the centroid of the foci. Euclidean distances between each pair of foci and between each labeled promoter focus and the nearest nuclear edge were calculated.

### QUANTIFICATION AND STATISTICAL ANALYSIS

The following statistical tests were used in this study: two-sample, one-tailed t test (Figures 2E, 2F, 3B, 3C, and S1D); and the Mann Whitney U-test (Figures 5C and 5D). Details for statistical tests performed are described in the indicated figure legends. All statistical tests were performed using MATLAB or R.

#### Modeling simulations

All models were simulated using the Gillespie algorithm provided in the Tellurium package in Python 2.7 (Choi et al., 2018). Plotting of simulation results was done in MATLAB. A detailed description of the models can be found in the mathematical appendix (see Methods S1: Mathematical Appendix, related to STAR Methods).

#### Image analysis of time-lapse movies

##### Image segmentation

Cell segmentation was performed in MATLAB (Mathworks, Natick, MA) using custom scripts described previously (Kueh et al., 2016; Ng et al., 2018). The segmentation algorithm was performed on CFP fluorescent signals as all transduced cells carried an H2B-mCerulean CFP reporter cassette. Briefly, images underwent (1) correction by subtraction of uneven background signal stemming from the bottom of the glass plate or the side of the PDMS microwells (2) Gaussian blur followed by pixel value saturation to fix uneven signal intensity within the nucleus of the cell and (3) Laplacian edge detection algorithm to identify the nucleus boundary. Non-cell objects were excluded via size and shape limit exclusions, and segmentation parameters were chosen such that the number of non-cell objects are < 1% of the total segmented cells.

##### Identification of live and dead cell population

In movies of cMyc or empty vector (EV) transduced cells, live and dead cells possessed distinct morphological features as observed in the CFP fluorescence channel. Live cell nuclei had a round, smooth oval shape while dead cell nuclei tended to be more granular, with small but very bright puncta. To provide unbiased, automated recognition of live and dead cells based on these features, we applied a Laplacian mask filter to each segmented cell to delineate the ‘smoothness’ of its signal, then applied a threshold-cutoff to identify regions with high CFP signal. The resultant list of object features were recorded for each cell object: 1) nuclei area, 2) perimeter, 3) fluorescent intensity, 4) puncta number, 5) mean puncta area, 6) mean puncta perimeter, and 7) area for puncta above the cutoff threshold. Approximately one hundred individual cell images (10% of each dataset) were then manually annotated as ‘live’ or ‘dead’. Annotations were then linked to the above feature matrix, and a decision tree supervised machine learning algorithm was then used to generate a model based on the annotated live/dead classification and matrix features of the training images (Figures S2A–S2C). Finally, built-in MATLAB model evaluation functions resubLoss and crossval were used to validate that mis-assignment error is below 15% for all datasets. This approach was utilized to provide an objective, automated method to distinguish between live and dead populations.

##### Bcl11b activation rate fitting

The following procedure was used to quantify *Bcl11b* activation rate from timelapse movies: first, the YFP and RFP signal intensity of segmented cells were calculated. Next, each cell object was classified as ‘live’ or ‘dead’, using classification prediction by trained model described in the previous section. Cells classified as ‘live’ were selected, and their YFP RFP fluorescence 2D histograms were then fitted to a two-component mixed 2D Gaussian model to obtain the fraction of YFP-OFF and YFP-ON cells in the population at a given time. To calculate background-corrected fluorescent values of the Bcl11b YFP and Bcl11b RFP signals, we calculated the pixel intensity of an annulus surrounding the segmented cell and subtracted this value from the raw signal intensity in the cell interior. This approach eliminates autofluorescence from the bottom of the glass plate as well as at the edge of the PDMS microwell.

To obtain the time evolution of Bcl11b biallelic population fractions from initial Bcl11b YFP-RFP+ population, cells were first filtered based on their ‘live/dead’ category, and only ‘live’ cells were included in subsequent calculations. We used a modified version of least-squares fit of a two-component mixed 2D Gaussian function described by Ng et al. (2018) to fit the 2D histogram of Bcl11b YFP and Bcl11b RFP fluorescence levels. Specifically, let y and r be the intensity of Bcl11b YFP and Bcl11b RFP fluorescence, respectively, the overall fit, *F*(*r*,*y*), is given by:

(Equation 1) $$F(r,y)=\overset{2}{\underset{i=1}{\sum }}f_{i}(r,y)$$

Each 2D Gaussian *f* is given by:

(Equation 2) $$f_{i}(r,y)=\frac{N_{i}}{2\pi \sigma_{r,i}\sigma_{y,i}\sqrt{1-\rho_{i}^{2}}}\cdot exp\;\left(-\frac{1}{2\left(1-\rho_{i}^{2}r^{2}\right)}\left[\frac{{\left(r-\mu_{r,i}\right)}^{2}}{\sigma_{r,i}^{2}}+\frac{{\left(y-\mu_{y,i}\right)}^{2}}{\sigma_{r,i}^{2}}+\frac{2\rho \left(r-\mu_{r,i}\right)\left(y-\mu_{r,i}\right)}{\sigma_{r,i}\sigma_{y,i}}\right]\right)$$

Here, *i* = 1,2 correspond to the red mono-allelic and biallelic populations, since all starting cells are red mono-allelic, we excluded the other two populations (non-expressing and yellow mono-allelic). *N*_*i*_ is the volume under the Gaussian curve when integrated over r and y and is the approximation for the number of cells in each population in Bcl11b RFP mono-allelic and biallelic states.

To fit our data to *F*(*r*,*y*), we followed a two-step process described previously (Ng et al., 2018): (1) We fitted Bcl11b YFP/RFP 2D histogram at an early time point (0 < t < 20) to *f*_1_(*r*,*y*)to obtain the means, standard deviations, and correlation coefficients (*u*_*r*,1_,*σ*_*r*,1_,*u*_*y*,1_,*σ*_*y*,1_,*p*_1_)) of the Bcl11b RFP mono-allelic population. At this early time point, cells remained inactive for the Bcl11b YFP allele. (2) Next, we fitted the 2D histograms of Bcl11b YFP/RFP levels at successive time bins of 20 hours, fixing the parameter of the first Gaussian *f*_1_(*r*,*y*), and enabling the parameters for the second Gaussian *f*_2_(*r*,*y*), to vary within bounds observed in the fluorescent distributions of Bcl11b biallelic populations. After fitting, the fraction of biallelic cell at a given time window centered on time t is given by:

(Equation 3) $$f_{2}^{obs}(t)=\frac{N_{2}(t)}{N_{1}(t)+N_{2}(t)}$$

The confident bounds for $f_{i}^{obs\;}(t)$ is given by:

(Equation 4) $$\delta f_{i}^{obs\;}(t)=f_{i}^{abs\;}\sqrt{{\left(\frac{\delta N_{i}}{N_{i}}\right)}^{2}+\frac{\overset{2}{\underset{i=1}{\sum }}\delta N_{i}^{2}}{{\left(\overset{2}{\underset{j=1}{\sum }}N_{j}\right)}^{2}}}$$

Afterward, the resulting fraction of biallelic cells as a function of time window centered at time t from the mixed Gaussian fit was then fitted to the probability density function of a first order process:

(Equation 5) $$F_{bi}(t)=1-e^{-\lambda t}$$

Where *λ* is the rate for activation of the initially silent Bcl11b-YFP allele. We chose this function for activation rate fitting since our histone dynamics simulations suggested that Bcl11b activation can be estimated as a first order stochastic process (see Figure 4D). For this function, fitting was done using the MATLAB fit function and a 95% confidence interval for the fit was recorded.

#### Population dynamics model and fitting

We built a mathematical model to describe the population dynamics of progenitor cells transfected with an empty vector (EV) and c-Myc. From initial inspection of time-lapse movies (Figure 3), progenitors transduced with c-Myc appear to expand more quickly than control progenitors, as expected. Faster expansion of c-Myc-transduced cells could be due to faster cell cycling or slower cell death. To disentangle these two effects, we quantified numbers of both live and dead cells over time (Figure 3B) and fit these data to population dynamics models to obtain division and death rates:

The model includes a population of live cells (*X*) with a division rate *k*_*b*_ and a death rate *k*_*d*_ to generate the observable dead cell population (*Y*). This population in turn has a clearance rate δ representing the process by which CFP level degrades and dead cells become undetectable.


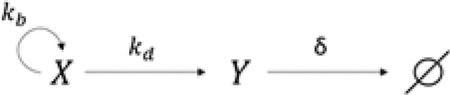


We then obtained the division rate *k*_*b*_ and the death rate *k*_*d*_ through a two-step curve fitting process. First the difference between these two rates*K* = *k*_*b*_ − *k*_*d*_was obtained by fitting the number of live cells over time::

(Equation 6) $$X(T)=X_{o}e^{\left(k_{b}-k_{d}\right)T}=X_{o}e^{KT}$$

where *X*_*o*_ is the initial number of live cells at the start of imaging. The death rate was then obtained by fitting the number of observable dead cells over time, which, given the above transition scheme, is given by the following: :

(Equation 7) $$Y(T)=Y_{o}P(T)+\overset{T}{\underset{t=1}{\sum }}X(t)\cdot k_{d}\cdot P(T-t)$$

Here *Y*_*o*_is initial observed number of dead cells at the start of imaging and *P*(*τ*)gives the probability of the dead cell remaining observable in the CFP fluorescent channel a period of time *τ* after its first appearance. In this model, whenever a cell starts to die, its probability of being detected decreases as per function *P*(*τ*), and the number of dead cells at a given time T is the sum of all the still-detectable dead cells generated since the start of imaging up until T. This decrease in detection probability arises because progressive dimming of CFP fluorescence, together with morphological changes following death results in the failure of the cells to be segmented.

We determined *P*(*τ*) empirically for EV and cMyc population separately by manually following 30 different dead cells and recording the time period in which it was detected and undetected until complete disappearance. We then calculated the fraction of dead cells that remained detectable after a given time had elapsed. An exponential function decay function was used to fit this ‘fraction detected’ curve and to estimate value for clearance rate (Figures S2B and S2C):

(Equation 8) $$P(\tau )=e^{-\delta \tau }$$

Here, *P*(*τ*) is the probability of a given dead cell to be detected under the CFP fluorescent channel after a period of time since its initial death. *δ* is the clearance rate of this process.

To fit imaging data to Equation 6, we classified segmented cell objects as live or dead using a trained machine learning model as described in ‘Image analysis of time-lapse movies’ section. Number of live cells as a function of time was fitted to Equation 6 using a MATLAB fit function and 95% confidence interval for the fit was recorded.

To fit imaging data to Equation 7, we tested a series of candidate *k*_*d*,*i*_values; for each *k*_*d*,*i*_, a predicted *Y*_*p*,*i*_(*t*)curve was generated based on Equation 7 where *T* = *t*_1_,*t*_2_,*t*_3_,…with *t*_*i*_ being the time point at which experimental measurement took place. *Y*_*p*,*i*_(*T*)is then compared to the experimentally observed dead cell number *Y*_*exp*_(*T*)using sum square error method:

(Equation 9) $$sse_{i}=\underset{t=t_{1},t_{2},t_{3},\ldots }{\sum }{\left[Y_{exp}(t)-Y_{p,i}(t)\right]}^{2}$$

The best fit *k*_*d*_value is chosen to be the value *k*_*d*,*i*_, whose *sse*_*i*_is the smallest.

In order to calculate the confidence bound of the fit, we first performed a nonlinear regression by calculating the residuals of the model’s predicted values *Y*_*p*_(*t*_*i*_):

(Equation 10) $$dY_{t_{i}}=Y_{exp}\left(t_{i}\right)-Y_{p}\left(t_{i}\right)$$

We then calculated to the Jacobian of the model function to estimate the covariance at each time point, given by:

(Equation 11) $$J_{t_{i}}=\frac{\partial Y_{p}\left(t_{i}\right)}{\partial k_{d}}$$

These inputs were used to estimate 95% confidence interval using MATLAB ‘Nonlinear regression parameter confidence intervals’ function nlparci.

A summary of results from data fitting are tabulated in Table S1.

## Supplementary Material

1

2

## Figures and Tables

**Table:** KEY RESOURCES TABLE

REAGENT or RESOURCE	SOURCE	IDENTIFIER
Antibodies		
Anti-mouse Ter119 Biotin (clone TER-119)	eBioscience	Cat#13-5921-85; RRID:AB_466798
Anti-mouse NK1.1 Biotin (clone PK136)	eBioscience	Cat#13-5941-85; RRID:AB_466805
Anti-mouse Gr-1 Biotin (clone RB6–8C5)	eBioscience	Cat#13-5931-86; RRID:AB_466802
Anti-mouse CD11c Biotin (clone N418)	eBioscience	Cat#13-0114-85; RRID:AB_466364
Anti-mouse CD11b Biotin (clone M1/70)	eBioscience	Cat#13-0112-86; RRID:AB_466361
Anti-mouse CD19 Biotin (clone 1D3/6D5)	eBioscience	Cat#13-0193-85; RRID:AB_657658
Anti-mouse CD3e Biotin (clone 145-2 C11)	eBioscience	Cat#13-0031-85; RRID:AB_466320
Anti-human/mouse B220 Biotin (clone RA3-6B2)	eBioscience	Cat#13-0452-85; RRID:AB_466450
Anti-human/mouse CD44 eFluor 450 (clone IM7)	eBioscience	Cat#48-0441-82; RRID:AB_1272246
Anti-mouse CD25 APC-eFluor 780 (clone PC61.5)	eBioscience	Cat#47-0251-82; RRID:AB_1272179
Streptavidin PerCP-Cyanine5.5	Biolegend	Cat#405214; RRID:AB_2716577
APC Annexin V	Biolegend	Cat#640919
Bacterial and virus strains		
Stable Competent *E.coli*	NEB	Cat#C3040
Chemicals, peptides, and recombinant proteins		
Recombinant Human Flt3-Ligand	PreproTech	Cat#300–19
Recombinant Human IL-7	PreproTech	Cat#200–07
Recombinant Human Stem Cell Factor (SCF)	PreproTech	Cat#300–07
Anti-H3K27me3 antibody	Active Motif	Cat#39156
Retronectin	Takara	Cat#T100B
DL1-ext IgG Protein	Gift from Irwin Bernstein (Varnum-Finney et al., 2000)	N/A
FuGENE 6 Transfection Reagent	Promega	Cat#E2691
UNC1999	Caymen Chemical	Cat#14621
GSKJ4	Caymen Chemical	Cat#12073
IOX1	Caymen Chemical	Cat#11572
GSK126	Caymen Chemical	Cat#15415
GSK343	Caymen Chemical	Cat#14094
Concavalin A	Bangs Laboraatories	Cat#BP531
protein A-MNase	gift from Steven Henikoff (Skene et al., 2018)	N/A
Deposited data		
T cell progenitor H3K27me3 CUT&RUN	This paper	Gene Expression Omnibus GSE134749
Thymocyte H3K27ac ChIP-sequencing	Davis et al., 2018	ENCODE accession ENCSR000CCH
Erythroblast H3K9me3 ChIP-sequencing	Davis et al., 2018	ENCODE accession ENCSR000DHN
T cell progenitor H3K27me3 ChIP-sequencing	Zhang et al., 2012	Gene Expression Omnibus GSE31235
DN3-DP thymocyte Hi-C	Hu etal., 2018	Gene Expression Omnibus GSE79875
Experimental models: cell lines		
OP9-DL1-GFP	Holmes and Zúñiga-Pflücker, 2009	N/A
Kasumi-1	ATCC	Cat#CRL-2724; RRID:CVCL_0589
Pheonix-Eco	ATCC	Cat#CRL-3214, RRID:CVCL_H717
Experimental models: organisms/strains		
Mouse: Bcl11b^RFP/YFP^	Ng et al., 2018	N/A
Oligonucleotides		
*Bcl11b* forward primer: TCCACCTACCAGACCCCGAA	IDT	N/A
*Bcl11b* reverse primer: CTTCTTCAAAGTGCTTGGCCTC	IDT	N/A
*PAX5* forward primer: CCAGGATGTGCTGCTGTCCCAG	IDT	N/A
*PAX5* reverse primer: CTCCCTGGTGCTGTGCACTGA	IDT	N/A
mir-30-shEed template: TGCTGTTGACAGTGAGCGAAGGCATTATAAGAATAATTAATAGTGAAGCCACAGATGTATTAATTATTCTTATAATGCCTCTGCCTACTGCCTCGGA	IDT	N/A
Recombinant DNA		
pBAD-mTagBFP2	Addgene	Cat #34632
pMSCV-miR-30	Gift from Johannes Zuber (Fellmann et al., 2013)	N/A
pMSCV-c-Myc-H2B-mCerulean	Kueh et al., 2016	N/A
pBanshee-CFP	Kueh et al., 2016	N/A
pCL-Eco	Imgenex	Cat#NBP2-29540
Software and algorithms		
Software: FlowJo (v10.0.8)	Tree Star	https://www.flowjo.com/
Software: MATLAB (R2018b)	MathWorks	https://www.mathworks.com/products/matlab.html
Software: R (v3.9)	N/A	https://www.r-project.org/
Rstudio (v1.2.5042)	N/A	https://rstudio.com/
Ggplot2 (v.3.3)	N/A	https://ggplot2.tidyverse.org/
Bedtools (v2.17.0)	Quinlan and Hall, 2010	https://bedtools.readthedocs.io/en/latest/
Samtools (v0.1.19-96b5f2294a)	Li et al., 2009	http://samtools.sourceforge.net/
Fiji (v2.0.0)	Schindelin et al., 2012	https://imagej.net/Downloads
BUnwarpJ	Arganda-Carreras et al., 2006	https://imagej.net/BUnwarpJ
Python(v3.6)	N/A	https://www.python.org
Other		
BD FACS Aria III Cell Sorter	BD Biosciences	N/A
NucleoSpin PCR Clean-up	Macherey-Nagel	Cat#740609.50
Illumina MiSeq	Illumina	N/A
Attune NxT Acoustic Focusing Cytometer	ThermoFisher Scientific	N/A
KAPA HyperPrep Kit	Roche	Cat#07962312001
PowerUp SYBR Green Master Mix	ThermoFisher Scientific	Cat#A25741
Ampure XP magnetic beads	Beckman Coulter	Cat#A63880
CFX96 Real-Time PCR Detection System	Bio-Rad	Cat#1855196
CD117 Microbeads	Miltenyi Biotec	Cat#130-091-224
LS Columns	Miltenyi Biotec	Cat#130-042-401
250mm-diameter PDMS circular micromesh arrays	Microsurfaces Pty Ltd	Cat#MMA-0250-100-08-01
Custom scripts for imaging analysis and simulations	This paper	https://github.com/KuehLabUW/Pease_et_al.2021

## Highlights

- Timed epigenetic switch delays *Bcl11b* activation over multiple cell cycles
- H3K27me3 levels, set by PRC2 and Kdm6a/b, tunably set this activation delay
- This activation time delay is set independently from cell cycle length
- Quantitative methylation-compaction model explains tunable, division-independent delays
