# Supplementary material for: Tunable, division-independent control of gene activation timing by a polycomb switch
Source: Cell Rep. Author manuscript; Available in PMC 2021 Apr 7. (PMC8024876; doi:10.1016/j.celrep.2021.108888)
Supplement: 1 [file NIHMS1686913-supplement-1.pdf]

Cell Reports, Volume *34*

Supplemental information

Tunable, division-independent control of gene activation timing by a polycomb switch

Nicholas A. Pease, Phuc H.B. Nguyen, Marcus A. Woodworth, Kenneth K.H. Ng, Blythe Irwin, Joshua C. Vaughan, and Hao Yuan Kueh

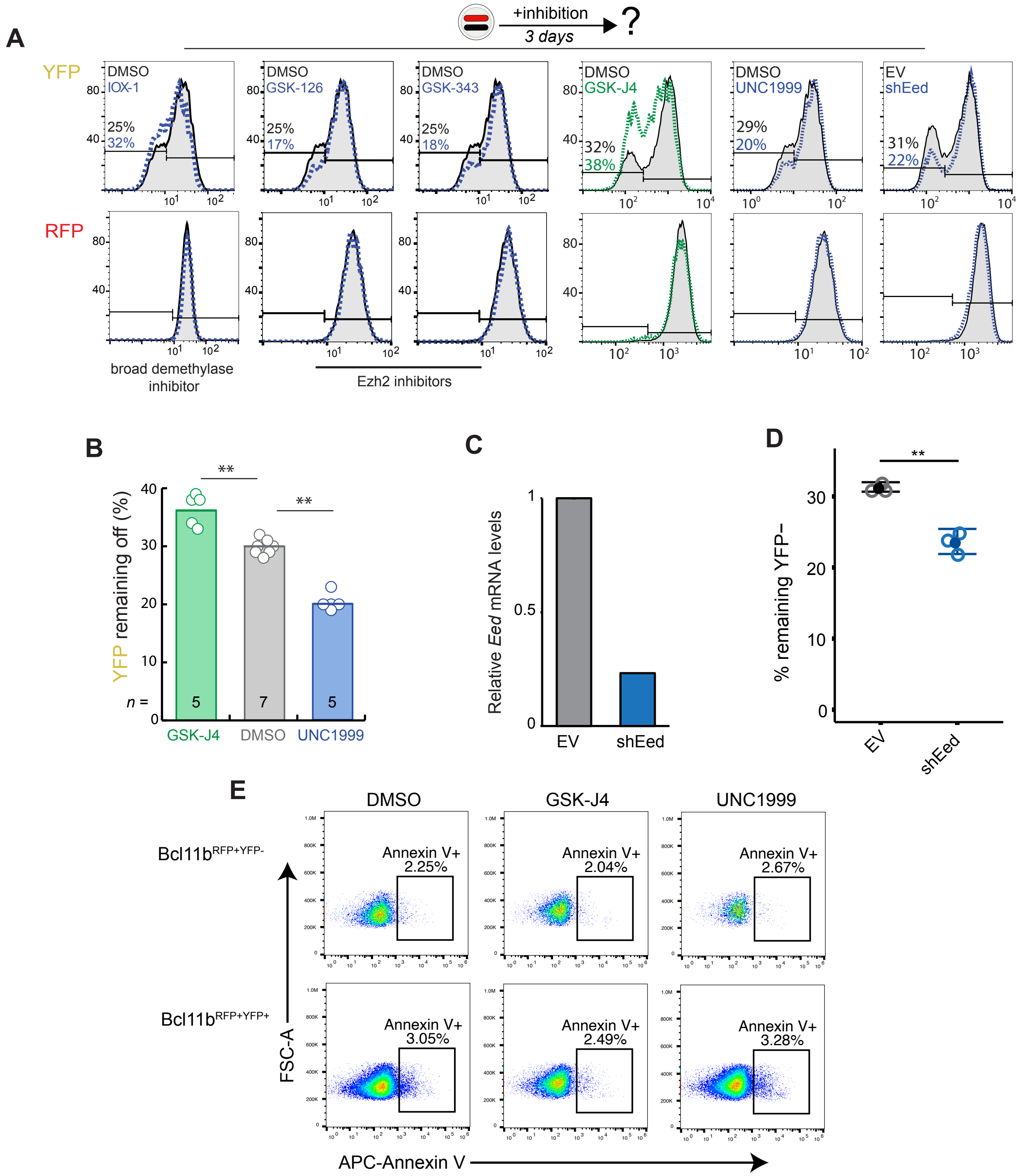

Supplementary Figure S1. Unrelated H3K27me3 perturbations modulate *Bcl11b* activation timing, related to Figure 2. (A) DN2 Bcl11b$^{RFP+YFP-}$ monoallelic expressing cells were sorted, re-cultured on OP9-DL1 cells in the presence of different small molecule inhibitors and analyzed by flow cytometry 3 days later. Structurally unrelated inhibitors show similar effects of decreasing or increasing *Bcl11b* activation probabilities as observed in Figure 2D (left). All histograms show that while the perturbations affect the all-or-none activation probability for the initially inactive alleles (top), the pertubations have no effect on the expression maintenance nor magnitude of the initially active alleles (bottom). (B) Mean percentage of cells remaining Bcl11b YFP-negative (n = independent experiments, *p < 0.05, **p < 0.01, two-sample t-test, two-tailed). (C) Relative mRNA levels of Eed were measured by qPCR. (D) Mean percentage of cells remaining YFP-negative after DN2 Bcl11b$^{RFP+/YFP-}$ monoallelic cells were transduced with retroviral constructs and recultured for 3 days (two-sample t-test, one-tailed, **p < 0.01, n = 3 independent experiments, error bars = 95% confidence interval). (E) Progenitors from Figure 2D (day 3) were stained with Annexin V to detect apoptotic cells.

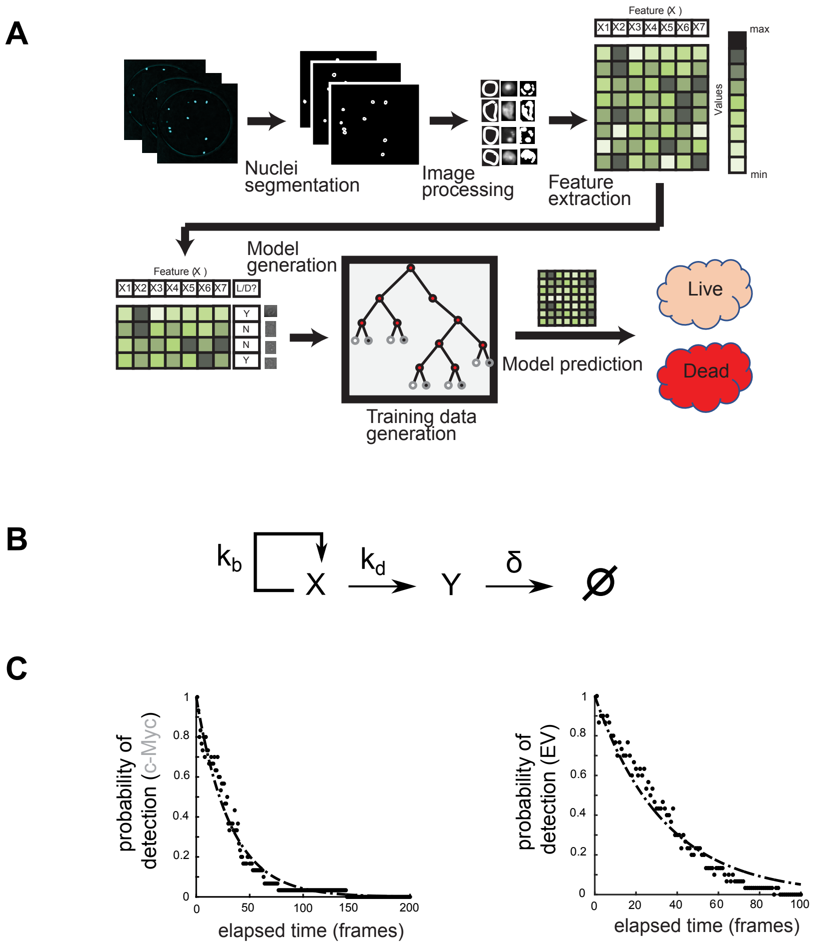

Supplementary Figure S2. Image processing pipeline for identification of live and dead cells in imaging experiments, related to Figure 3. (A) Cyan fluorescent protein (CFP) signals from transduced cells are used for segmentation of individual cells. Segmented populations were analyzed using image processing tools in MATLAB, and specific features such as object area, perimeter, and CFP intensity were collected. Approximately 10% of the total individual cells were then manually labeled as live or dead and fed into classification tree machine learning algorithm to generate a classification model. The rest of segmented cells are classified subsequently via the trained model. (B) Mathematical model describing the population dynamics of Bcl11b$^{RFP+/YFP-}$ cells transduced with c-Myc and empty vector (EV). The model includes a population of live cells (X) with a birth rate k_b and a death rate k_d that generates the dead cell population (Y). This population then has a permanent clearance rate of δ, indicating the process in which CFP degrades in these cells and the dead cells become undetected. (C) Experimentally determined decay of dead cells' detectability in time-lapsed movies. Individual dead cells were followed until their CFP level completely diminished and was undetectable by the segmentation algorithm, and the elapsed time was recorded. Thirty different individuals cells were recorded for each c-Myc and EV populations. Data was fit to an exponential decay function $P(\tau) = e^{-\delta\tau}$ with $\delta = 0.032$ per frame for c-Myc and $\delta = 0.030$ per frame for EV population.

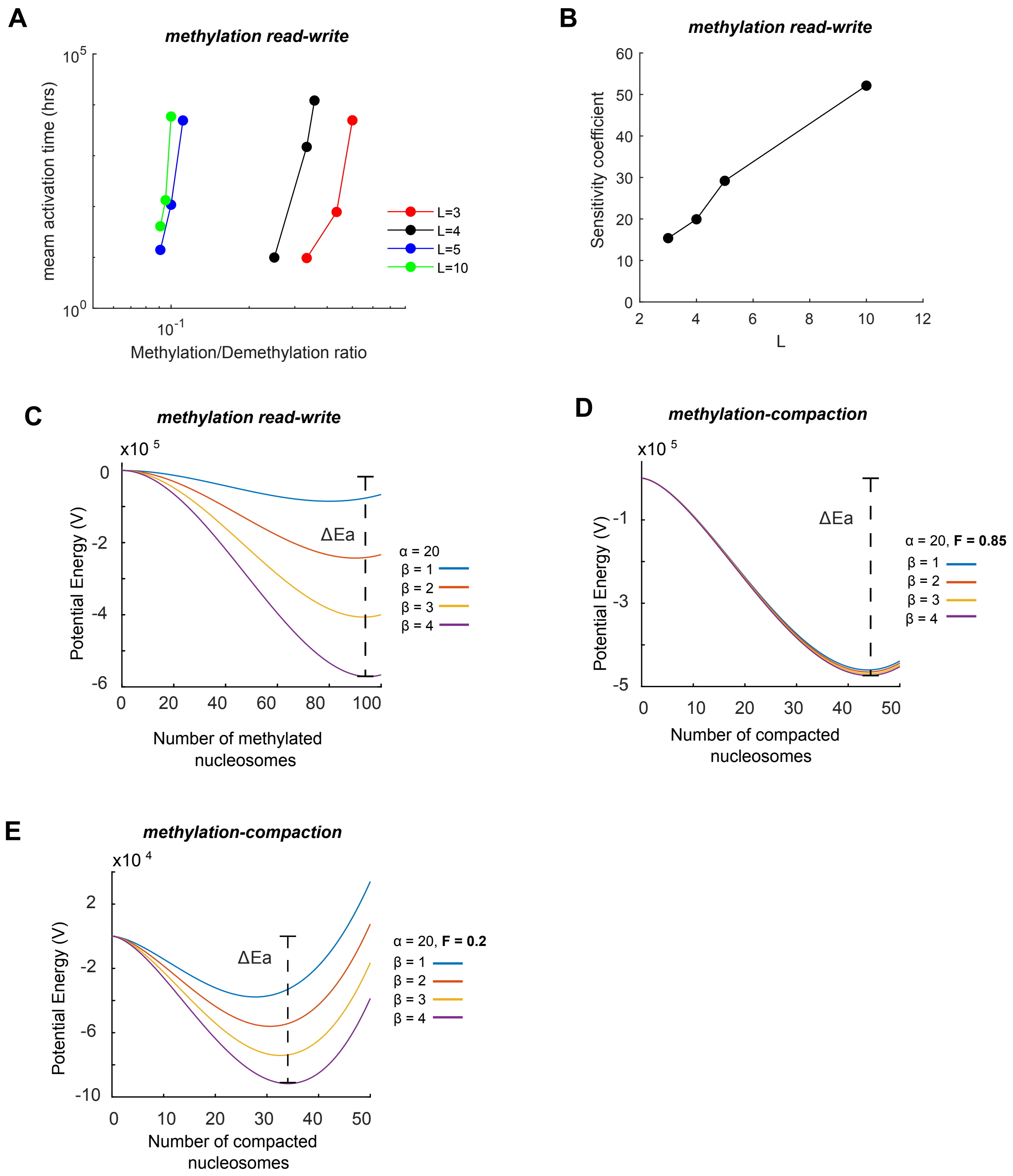

Supplementary Figure S3. Activation energy is robust to changes in methylation rate when interaction affinities between methylated and demethylated nucleosomes are similar, related to Figure 4. (A) Mean activation time as a function of the methylation/demethylation ratio derived from methylation read-write model simulations while decreasing the number of nulceosomes within 'reach' of a the PRC2 complex, L. (B) Sensitivity coefficient (ΔlogY/ΔlogX) as a function of L. (C) Potential energy landscapes of methylation read-write model. (D-E) Potential energy landscapes of the methylation-compaction model. Parameter F dictates how sensitve nucleosome compaction affinity is to demethylation (i.e. when F is high, the compaction affinity is only moderately affected by demethylation; see Mathematical Appendix for more details). The activation energy barrier (Ea) is defined as the potential energy (V) height between the local maximum and local minimum of the potential energy landscape. Each potential curve was plotted with demethylation parameter set to 20 hrs^{-1} and methylation rate parameter as indicated by the curve's color see Mathematical Appendix).

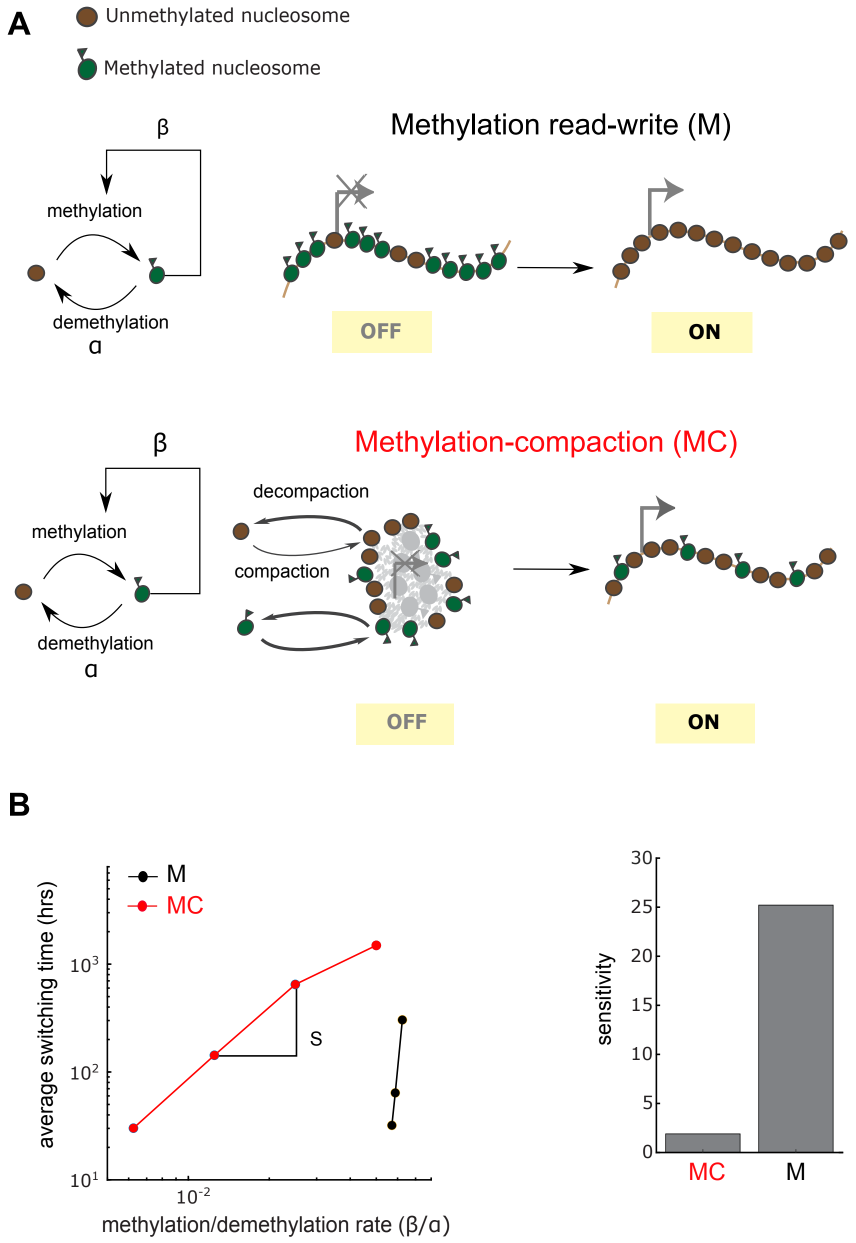

Supplemental Figure S4. Cooperativity in methylation-compaction model increases switching time tunability compared to methylation read-write model, related to Figure 4. (A, top) Methylation read-write model enables gene activation via complete eviction of methylation marks. (A, bottom) Methylation compaction model with cooperative methylation rate. A nucleosome's methylation rate increases with the number of methylated nucleosomes in the system. (B) Average switching times as a function of methylation (β) and demethylation rate constant (α) ratio for the methylation read-write (black) and methylation-compaction (red) models. Sensitivity coefficient (ΔlogY/ΔlogX) for each plot was calculated by taking the slope of the linear fit y = ax+b for the methylation model data set and the last 5 data points for the compaction model.

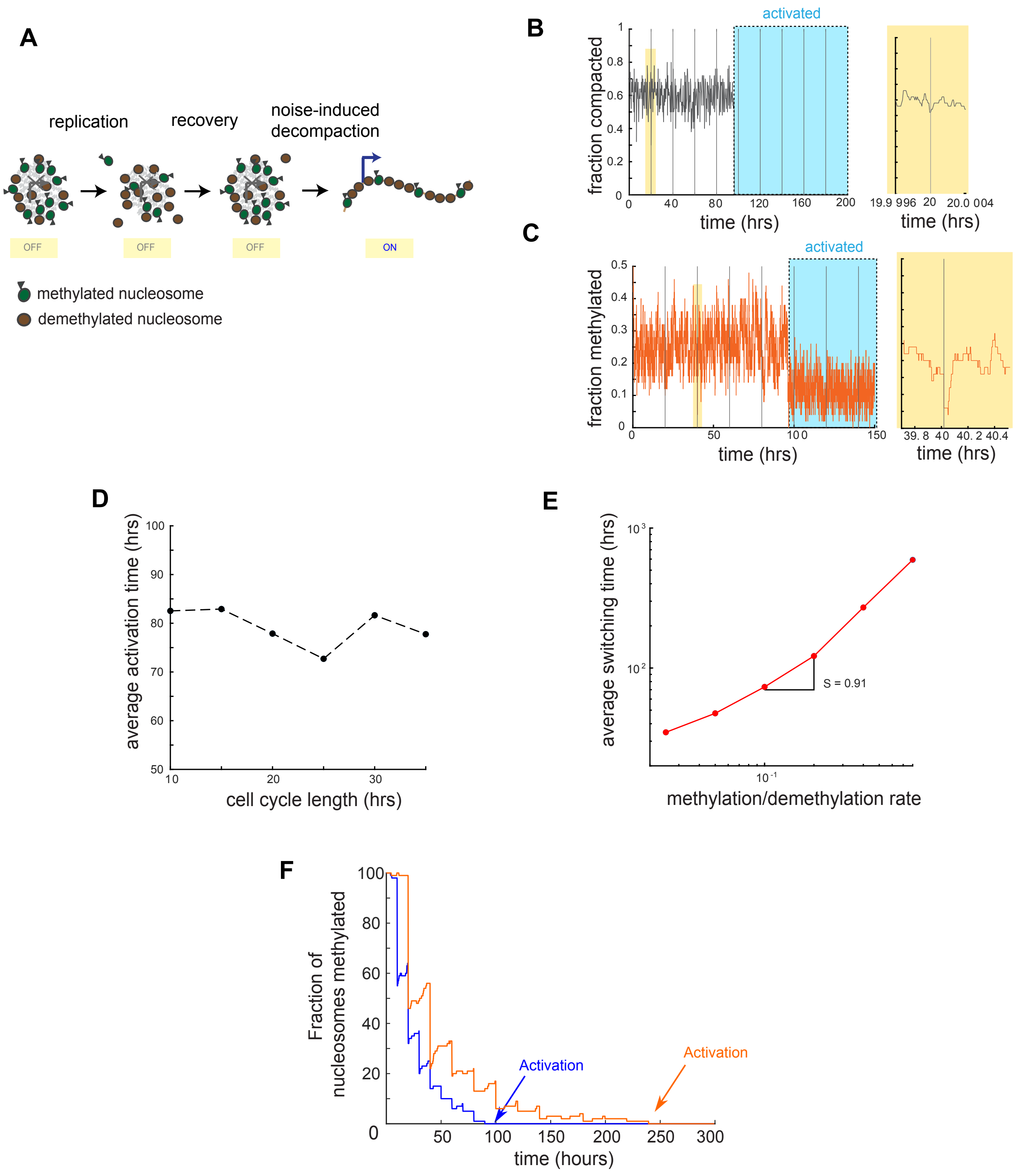

Supplementary Figure S5. Perturbations to the compacted state by DNA replication does not affect tunability or division-independence in the methylation compaction model, related to Figure 4. (A) Modified methylation compaction model where every cell division leads to 50% reduction in methylation state and 10% reduction in compaction state. (B-C) Compaction and methylation state as a function of time. Zoomed in first replication event. (D) Average switching time of the system as a function of cell cycle length. (E) Average switching times as a function of methylation and demethylation rate ratio. Tunability coefficient S (Δ logY/ΔlogX) for each plot was calculated by taking the slope of the linear fit y = ax+b for the methylation model data set. (F) Fractions of methylated histones are shown for the methylation read-write model with cell division lengths set to be 10 hrs (blue) and 20 hrs (orange). Methylation and demethylation rates were set to 0.001 per hour (see Mathematical Analysis for more details).

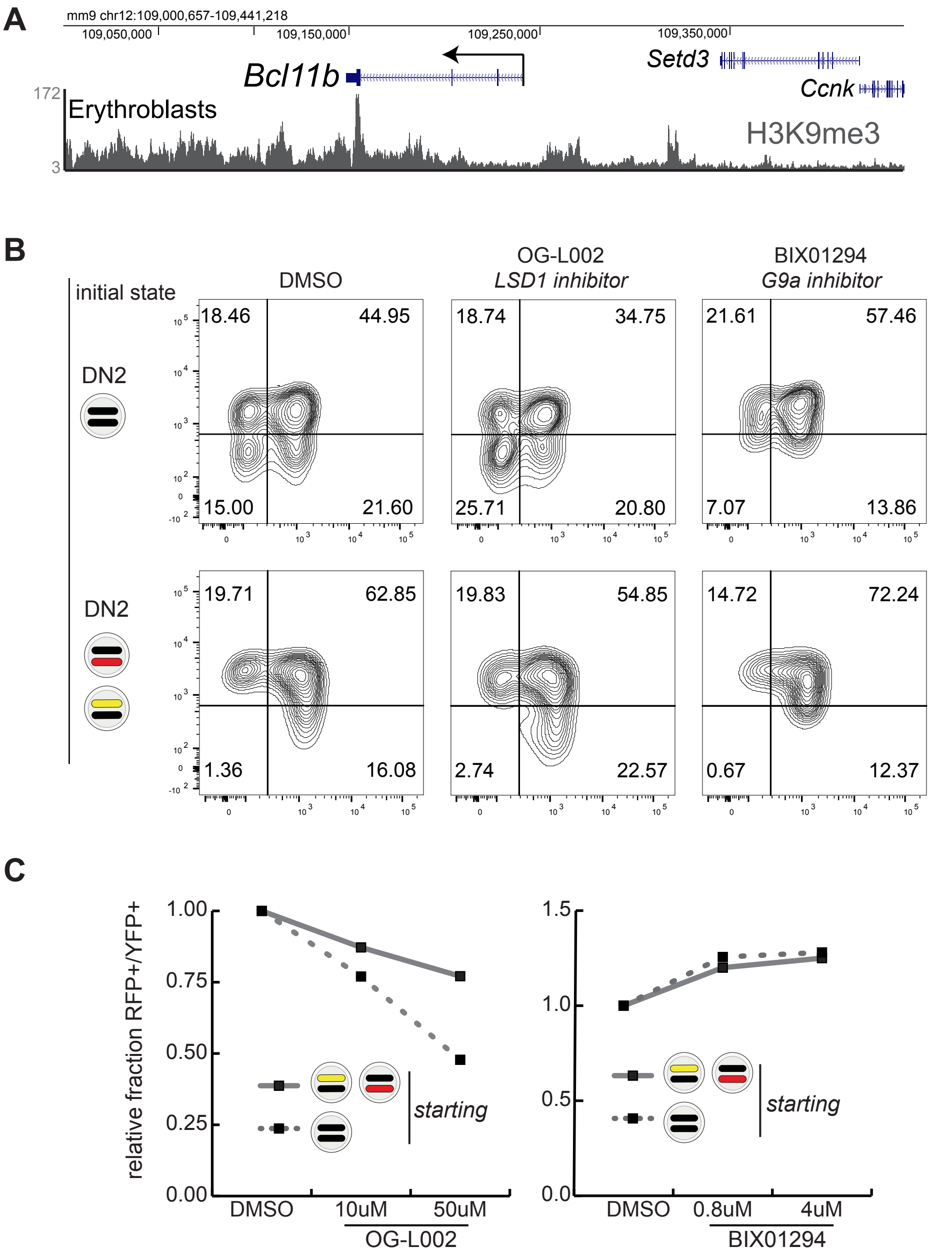

Supplemental Figure S6. H3K9-modifying enzymes regulate *Bcl11b* activation probability, related to Figure 4. (A) H3K9me3 ChIP-sequencing data from mouse erythroblasts (Davis et al., 2018; ENCODE accession ENCSR000DHN) visualized in the UCSC Genome Browser. (B) DN2 progenitors, either Bcl11b$^{RFP-/YFP-}$ (top) or Bcl11b$^{RFP-/YFP+}$ and Bcl11b$^{RFP+/YFP-}$ (bottom), were purified and re-cultured on OP9-DL1 cells for 3 days in the presence of OG-L002 (LSD1 inhibitor) or BIX01294 (G9a inhibitor) before analyzing reporter expression. (C) Relative fraction of progenitors RFP+/YFP+ after 3 days normalized to the respective DMSO controls.

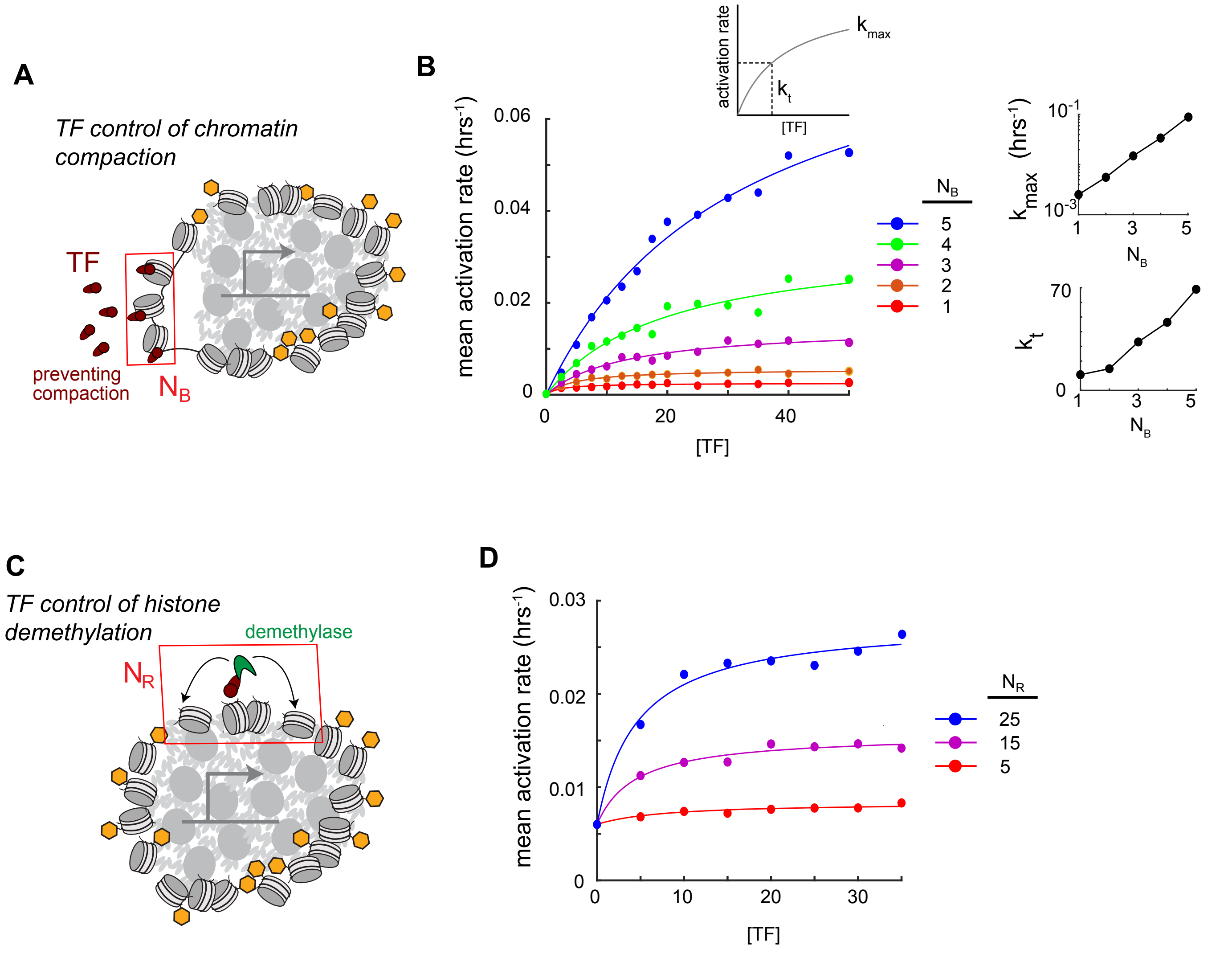

Supplementary Figure S7. Transcription factors can control gene activation timing by modulating nucleosome methylation or compaction, related to Figure 4. (A) In the MC model, pioneer TFs can bind to nucleosomes, preventing them from entering the compacted assembly. N_B indicates the number of TF binding sites, assumed to occur on different nucleosomes. (B) Mean simulated gene activation rate against TF concentration for different values of N_B (left). Maximal activation rate (k_{max}) and half-maximal concentration (k_t) as a function of the number of nucleosomes bound (N_B) (right). (C) TFs can also induce demethylation of a number of nucleosomes, N_R, within its vicinity of binding. (D) Mean gene activation rate as a function of TF concentration for different values of N_R.

	cMyc					
	Live Rate (*K*)	95% CI	Death Rate (k_d)	95% CI	Division Rate ($k_b = K + K_d$)	95% CI
Trial 1	0.031 hrs^{-1}	± 0.001	0.034 hrs^{-1}	± 0.001	0.065 hrs^{-1}	± 0.001
Trial 2	0.043 hrs^{-1}	± 0.001	0.013 hrs^{-1}	± 0.000	0.057 hrs^{-1}	± 0.001
Trial 3	0.035 hrs^{-1}	± 0.001	0.027 hrs^{-1}	± 0.001	0.069 hrs^{-1}	± 0.001

	EV					
	Live Rate (*K*)	95% CI	Death Rate (k_d)	95% CI	Division Rate ($k_b = K + K_d$)	95% CI
Trial 1	0.017 hrs^{-1}	± 0.001	0.028 hrs^{-1}	± 0.001	0.045 hrs^{-1}	± 0.001
Trial 2	0.014 hrs^{-1}	± 0.001	0.007 hrs^{-1}	± 0.001	0.027 hrs^{-1}	± 0.001
Trial 3	0.024 hrs^{-1}	± 0.001	0.019 hrs^{-1}	± 0.001	0.043 hrs^{-1}	± 0.001

Supplemental Table S1, related to Figures 3 and S2. Tabulated doubling (K) and death (k_d) rates calculated from data fitting of live and dead populations from three independent imaging experiments. Data was fit to population dynamics model described in Statistical and Quantitative Analysis section.

Methods S1: Mathematical Appendix, related to STAR Methods

Introduction

To understand the timed epigenetic switch controlling the *Bcl11b* activation, we used mathematical modeling to analyze a series of candidate biophysical mechanisms. This mathematical modeling analysis seeks to uncover the essential emergent properties of the switch, namely (1) its irreversible, all-or-none nature; (2) its long, stochastic time delay; (3) the heritability of its inactive and active states over DNA replication; and (4) its tunability with respect to changes in H3K27me3 levels and modifying-enzyme activity.

We consider two main candidate models. In the methylation read-write model (M), individual nucleosomes within in a one-dimensional lattice can be methylated or unmethylated. Gene expression is assumed to occur when the total fraction of methylated nucleosomes in this lattice falls below a threshold value. In the methylation compaction (MC) model, individual nucleosomes are also methylated and demethylated; in addition, these nucleosomes also interact to form a compacted assembly with rates dependent on their H3K27me3 state. Unlike the methylation read-write model, gene expression does not depend directly on H3K27me3 levels, but on the compaction state of the nucleosome assembly, which in turn depends on methylation states of individual nucleosomes. Both models explicitly model DNA replication as a process involving random segregation of modified nucleosomes into daughter strands. From our analysis, we find that the methylation-compaction mechanism, but not the methylation read-write mechanism, explains the emergent behaviors of the timed epigenetic switch controlling *Bcl11b* activation, and thus represents our favored model.

Model I: The Methylation Read-Write Mechanism (M)

Here, we adopt a standard framework for histone modification dynamics previously shown to generate multi-stability (Angel et al., 2011; Dodd et al., 2007). In this model, individual nucleosomes reside in a one-dimensional lattice, and exist in two states, a methylated state, corresponding to an H3K27 tri-methylated state, and demethylated state. We do not describe multiple demethylated states in our model (i.e. mono-methylation, di-methylation, and an un-methylated state), though our analysis, together with previous work (Dodd et al., 2007), indicates that our main conclusions should also hold in more complex models with additional states. As with previous models, the methylation rate of a given nucleosome depends on the number and distance of methylated nucleosomes in its vicinity, reflecting observations that PRC2 can bind and be activated by H3K27me3-marked nucleosomes to write H3K27me3 on neighboring nucleosomes. The positive feedback generated by this methylation read-write mechanism provides a basis for bi-stabilty in this model. Here, demethylation is taken to occur at a first order rate. We assume there is no spontaneous methylation in the absence of existing methylated nucleosomes; thus, once all nucleosomes are demethylated, the system irreversibly enters an activated state.

Methylation. We explicitly model mark binding and methyltransferase activities of the PRC2 complex, as well as the methylation state of each individual nucleosome. Take the gene locus to a linear array of N nucleosomes. Let $i = 1..N$ denote the index for the ith nucleosome, and let p_i be its H3K27 methylation state. $p_i = 0$ denotes the de-methylated state while $p_i = 1$ denotes the methylated state.

Let u' and u denote the transitions between the methylation state and demethylation state, respectively. The model is set up as follows:

For $i \in \{1, .. , N\}$:

$$u': (p_i = 0) \rightarrow (p_i = 1)$$

$$u: (p_i = 1) \rightarrow (p_i = 0)$$

With:

$$Pr(u') = \beta \cdot (1 - p_i) \cdot \sum_{j \neq i} \quad p_j \cdot e^{-\left(\frac{j-i}{L}\right)^2} \qquad (1)$$

$$Pr(u) = \alpha \cdot p_i \qquad (2)$$

The parameter L can be interpreted as the 'reach' of the PRC2 complex to neighboring nucleosomes. A large value of L indicates a long length scale for nucleosome interactions. This effect is set to have a gaussian shape so that nucleosome closest to the anchored PRC2 complex has the highest methylation rate. Similar distributions of activity have been reported for artificially tethered enzymes (Hass et al., 2015), as well as for histone modifications around transcription factor binding sites (Heinz et al., 2010). Moreover, we assume periodic boundary conditions for the one-dimensional lattice, though similar results were observed with other non-repeating boundary conditions (not shown).

Cell division. To model the transmission of histone marks across cell divisions, we assume that methylated nucleosomes segregate randomly to the two daughter DNA strands upon replication; thus, each nucleosome position has a one-half probability of inheriting a nucleosome that is methylated. Experimental evidence suggests that approximately half of total global H3K27me3 partitioning of parental marks to the subsequent generations (Alabert et al., 2015).

From stochastic simulations of this model, we find that this methylation read-write mechanism can generate a time-delayed, stochastic switches from an inactive H3K27me3-high state to an active state without H3K27me3 (Fig. 4A); however, switching times are hypersensitive to mild changes to methylation and de-methylation rates (Fig. 4B), and therefore inconsistent with the graded changes in switching times observed upon inhibition of PRC2 methyltransferase or Kdm6a/b demethylases (Fig. 2). To understand the origins of this hypersensitivity, we re-formulate this model using a chemical kinetics framework amenable to analysis using transition state theory. To do so, we first consider the limit where $L \rightarrow \infty$, such that each H3K27me3-bound PRC2 methylates all other un-methylated nucleosomes with the same reaction rate. In this limit, we can completely describe the state of the system by a single variable, the number of methylated nucleosomes *N'*. As the rates of adding or subtracting one methylated nucleosome from the system would reduce to become a function of *N'*, independent of spatial arrangement. Consequently:

$$N \underset{k_2}{\overset{k_1}{\rightleftharpoons}} N'$$

where

$$k_1 = \beta N'(N_T - N') \qquad (3)$$

$$k_2 = \alpha N' \quad (4)$$

and N_T is the total number of nucleosomes. The master equation describing the time evolution of this system is given by:

$$\frac{\partial p_n}{\partial t} = -[k_1(n) + k_2(n)] \cdot p_n + k_1(n-1)p_{n-1} + k_2(n+1)p_{n+1} \quad (5)$$

where p_n is the probability of having N' methylated nucleosomes. When the total number of nucleosomes is large, we can approximate the number of methylated nucleosomes to be a continuous variable x. In this limit, we can rewrite the master equation as Fokker-Planck equation:

$$\frac{\partial p(x,t)}{\partial t} = \frac{\partial}{\partial x}[v(x)p(x)] + \frac{1}{2} \cdot \frac{\partial^2}{\partial x^2}[D(x)p(x)] \quad (6)$$

where, we have ignored third and higher order terms, and where:

$$v(x) = k_1(x) - k_2(x) \quad (7)$$

$$D(x) = k_1(x) + k_2(x) \quad (8)$$

Given the velocity and diffusion constants for this system as a function of methylated nucleosome number, the switching of the system is essentially given by the first-passage time of the system to reach the absorbing state $x = 0$. A closed-form solution of this first-passage time distribution for the given rate functions is hard to obtain; Nevertheless, we note that our system operates in the regime where the timescales of individual methylation and demethylation reactions are much shorter than switching times for this system. In this regime, switching times are well described by the Kramer's theory for escape of a Brownian particle over a potential well (Kramers, 1940), and would thus approximately scale exponentially with the height of a potential energy barrier. We can obtain the functional form of this potential barrier by relating it to the velocity function:

$$-\frac{dV}{dx} = v(x) \quad (9)$$

From equations (3), (4), and (7), we can then integrate the system to explicitly derive the potential function:

$$V(x) = \frac{\beta}{3}x^3 + \frac{\alpha - \beta N_T}{2}x^2 + W \quad (10)$$

Where W is an arbitrary number. A plot of $V(x)$ is demonstrated in Fig. S3C. The energy landscape possesses a local minimum at a nonzero value of x, indicating the metastable state. The landscape has a local maximum near $x = 0$. State switching occurs when the system reaches the absorbing state $x = 0$. Thus, we define E_a as the height in $V(x)$ between the local maxima and the metastable state minima. When we plotted this potential energy for different values of β (Fig. S3C), we found that moderate changes in β led to significant changes in potential well height. As switching rates scale roughly exponentially with well height, we would expect this system would show extreme sensitivity in switching times with respect to changes in methylation rate changes.

Model II: The Methylation Compaction Mechanism (MC)

Because the methylation read-write mechanism above does not account for the tunable characteristics of the *Bcl11b* activation timing switch, we considered a second model, where histone methylation facilitates interactions between nucleosomes to enable the stable maintenance of a repressed, compacted chromatin state at the *Bcl11b* locus. There are multiple mechanisms by which H3K27me3 could facilitate interactions between nucleosomes: H3K27me3 could recruit polycomb repressive complex 1 (PRC1), which could oligomerize through contacts on its Bmi1 or Phc subunits (Eskeland et al., 2010; Gray et al., 2016; Isono et al., 2013; Kahn et al., 2016), or undergo weak, multivalent interactions on its Cbx2 subunit that result in liquid-liquid phase separation (Howard, 2001; Larson et al., 2017). Alternatively, H3K27me3 could modulate affinities of weak multivalent interactions between nucleosomes (Gibson et al., 2019), and thereby modulate their ability to phase separate.

The methylation compaction model consists of two main modules: (1) a H3K27 methylation and demethylation mechanism, and (2) a dynamic chromatin decompaction mechanism linked to H3K27me3 modification state that ultimately underlies gene switching. In our description of compaction dynamics, we do not explicitly model the spatial extent of the compacted nucleosomal assembly; instead, we adopt a mean-field approach that is established in models of cytoskeletal polymer dynamics (Erickson and Pantaloni, 1981; Jackson and Berkowitz, 1980). With this approach, the numbers of un-methylated and methylated nucleosomes within a compacted assembly are given by C and C' respectively, along with those outside the assembly are given by D and D' respectively. As a result, the dynamical system is described by four states: 1) Compacted-Methylated 2) Compacted-Demethylated 3) Decompacted-Methylated and 4) Compacted-Demethylated:

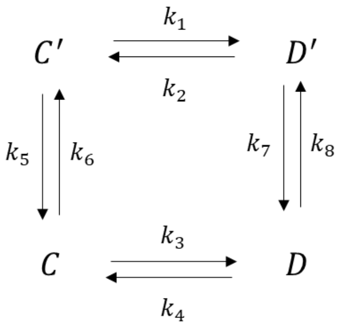

Here, C', C, D', and D denote the number of nucleosomes in these states, respectively. k_1 to k_8 denote the transition rates between them, which will be defined below. The gene is taken to be activated when all nucleosomes exist in a de-compacted state. The two mechanisms are intertwined so that methylation states affect compaction rates and vice versa. Detailed descriptions of the rates are given below:

Methylation. In this model, un-methylated nucleosomes convert into a methylated state with a first-order rate constant β. We assume this rate constant is the same regardless of whether nucleosomes are inside or outside the compacted assembly. Methylated nucleosomes convert into a de-methylated state with a rate constant of α if the nucleosome is outside the assembly (D'), or a lower rate constant of $f\alpha$, $(f < 1)$ if the nucleosome is inside the assembly. This lower rate constant

assumes that the demethylation reaction is less efficient on compacted nucleosomes, possibly due to competition for demethylase binding by compaction proteins, or due to the exclusion of demethylases through steric occlusion or phase separation. The rates describing these reactions on the four nucleosomal species are given by:

$$C' \underset{k_6}{\overset{k_5}{\rightleftarrows}} C \qquad D' \underset{k_8}{\overset{k_7}{\rightleftarrows}} D$$

Where:

$$k_5 = f\alpha\, C' \qquad (11)$$
$$k_6 = \beta\, C \qquad (12)$$

$$k_7 = \alpha\, D' \qquad (13)$$
$$k_8 = \beta\, D \qquad (14)$$

H3K27 methylation and demethylation rates are based on the catalytic activity of the Ezh2 subunit of the PRC2 complex and Kdm6a/b demethylases, respectively. Specifically, these rate constants were chosen to represent the conversion between H3K27me2 and H3K27me3. For simplicity, we do not model the H3K27me-binding dependent H3K27 methylation activity previously described (Margueron et al., 2009), though we show below that explicit modeling of this read-write effect would not significantly alter the conclusion of the model. Kdm6a/b demethylate H3K27me3 (Agger et al., 2007), and to our knowledge no cooperative activity of these complexes have not been reported.

Compaction. We adopt a mean-field description of the compacted nucleosomal assembly, following kinetic models of multi-stranded cytoskeletal polymer assembly (Howard, 2001). This description assumes that the nucleosome assembly is a roughly spherical structure held together by weak, multivalent interactions between individual nucleosomes, and can add or lose individual nucleosomes at its surface. Both methylated and demethylated nucleosomes can incorporate into the assembly; thus the assembly has a total size of:

$$C_T = C + C' \qquad (15)$$

where C and C' represent the number of methylated and demethylated nucleosomes in the assembly, respectively. Unlike other polymer models (MacPherson et al., 2018; Nuebler et al., 2018), we do not explicitly model physical connections between nucleosomes due to DNA; such connections would be expected to result in a spatial dependence of reaction rates within this chromatin domain; however, as the entire domain (100 nucleosomes) has a length scale greater than the persistence length of chromatin (~15-20 nucleosomes, from (Arbona et al., 2017)), and would thus enable free interactions between non-neighboring nucleosomes, we would expect the essential properties of our minimal model in a more realistic physical model that incorporates nucleosome connectedness.

The addition and removal of methylated and demethylated nucleosomes from the assembly is described by the following rate equations:

$$C' \underset{k_2}{\overset{k_1}{\rightleftarrows}} D' \qquad\qquad C \underset{k_4}{\overset{k_3}{\rightleftarrows}} D$$

Where:

$$k_1 = \frac{\delta}{C_T^{\frac{1}{3}}} C' \qquad (16)$$

$$k_2 = \lambda\, C_T^{\frac{2}{3}} D' \qquad (17)$$

$$k_3 = \frac{\delta}{C_T^{\frac{1}{3}}} C \qquad (18)$$

$$k_4 = F\,\lambda\, C_T^{\frac{2}{3}} D \qquad (19)$$

if $C_T > C_N$

$$k_1 = \frac{\delta}{C_T^{\frac{1}{3}}} C' \qquad (20)$$

$$k_2 = 0 \qquad (21)$$

$$k_3 = \frac{\delta}{C_T^{\frac{1}{3}}} C \qquad (22)$$

$$k_4 = 0 \qquad (23)$$

if $C_T < C_N$

Here, methylated nucleosomes incorporate into the compacted assembly with a rate constant λ; however, importantly, demethylated nucleosomes can also incorporate into the assembly with a reduced rate constant $F\lambda$ (where $F < 1$). The effect of methylation state on compaction rate is experimentally observed in instances such as recruitment of PRC1 complex by H3K27me3 marks (Kahn et al., 2016). The complex's subunits such as Ring1B and Phc-1 have been shown to be important in chromatin compaction and gene silencing (Eskeland et al., 2010; Francis et al., 2004; Isono et al., 2013). However, as PRC1 recruitment is not the only compaction mechanism *in vivo*, and because PRC1 can bind to nucleosomes independently of H3K27me3 (Francis et al., 2004), this model treats methylation as only a part, but not solely responsible for chromatin condensation. In choosing rate constants; we assume that compaction and decompaction is faster than histone methylation and demethylation rates, though timescales for both processes are assumed to be much faster than that for cell division. Fast compaction kinetics relative of modification is supported by *in vitro* studies of H3K27me3 methylation and demethylation kinetics, as well as *in vitro* DNA compaction by $HP1_{\alpha}$ and chromatin condensation experiments (Kristensen et al., 2011; Ladoux et al., 2000; Larson et al., 2017; Sneeringer et al., 2010).

The reaction rates for nucleosome incorporation (loss) scales with assembly size as $\sim C_T^{\frac{2}{3}}$ ($\sim C_T^{-\frac{1}{3}}$), as these reactions only take place on the surface of the assembly. Assuming a compacted nucleosome complex is spherical, the compaction rate would thus be proportional to the surface area. Likewise, the decompaction rate is also proportional to the surface area but reversely proportional to the total number compacted nucleosome in the complex.

In this description, there is a critical threshold number of compacted nucleosomes, C_N, below which the complex is thermodynamically unstable. The existence of a minimal nucleus size is a fundamental property of phase-separated assemblies held together by weak-multivalent, whereby addition of a new subunit to an already formed complex is thermodynamically more favorable than formation of the initial nucleus itself (Erickson and Pantaloni, 1981; Jackson and Berkowitz, 1980). Below this critical threshold C_N, the compacted assembly disintegrates, and gene turns on.

Cell division. Heritability of histone marks and chromatin states are crucial in maintaining gene expression states across cellular generations. As with the methylation read-write model above, we assume that methylated nucleosomes partition randomly between two daughter strands upon replication; the total number of nucleated nucleosomes is then obtained by sampling from binomial distribution with one half probability and N equal to the total number of nucleosomes at the point of DNA replication. Furthermore, we assume that compacted nucleosomes persist within a compacted assembly reside upon passage of DNA polymerase. This model feature assumes that new nucleosomes rapidly incorporate into a compacted assembly after passage of DNA polymerase; however, in the subsequent version of this model below, we will relax this assumption to allow for disruption of compaction state by DNA polymerase passage (see below).

From Monte-Carlo simulations, we found that this dynamic methylation compaction model can recapitulate all the essential emergent properties of the *Bcl11b* activation switch. Specifically, this model shows the following dynamic properties:

1) *Irreversible all-or-none switching to an H3K27me3-low, de-compacted state.* From simulations, we found that the system adopts a stable compacted assembly of nucleosomes with higher H3K27me3 marking density, but switches abruptly to a de-compacted state with lower H3K72me3 levels. As there is no re-nucleation of the compacted assembly after its elimination, this de-compacted state represents an absorbing, permanently active expressing state. The abrupt decrease in the H3K27me3 levels arises because compacted nucleosomes demethylate at a lower rate; thus, upon total decompaction, the percent of methylated nucleosomes lowers to a new steady state level.

2) *Noise induced gene activation.* Transition to the completely decompacted state, or gene activated state, occurs via stochastic deviation of the system from its compaction meta-stable state. Activation is triggered when the system reaches below the threshold number of compacted nucleosomes.

3) *Tunable activation rates*. The model is able to generate a gene switch with slow, tunable activation rate. Delay in activation is in order of days and can be finely adjusted by modifying methylation and demethylation rates, and/or changing H3K27me3 levels at the gene locus (Fig. 4E), as experimentally observed (Fig. 2). This ability to tune activation rates by changing H3K27me3 densities distinguishes this methylation compaction model from the methylation read-write model above, and thus represents a more plausible model for describing the activation mechanism of this switch. Why is this model uniquely tunable? In this model, locus de-compaction and gene activation are determined by a dynamic balance between rates of nucleosome entry or exit from a compacted assembly. The system still be sensitive to changes in these rates; however, as demethylated nucleosomes can still enter and exit a compacted assembly

at a reduced rate, changes in the fraction of demethylated nucleosomes would cause a fine change in these entry or exit rates, and thus give rise to a plausible tuning parameter for controlling activation timing.

4) *Division-independent timing control*. When the cell cycle length is changed in this model, activation kinetics remain largely unaffected, implying that the methylation-compaction mechanism functions as a cell division-independent delay timer. These conclusions hold, as long as the dynamic methylation and compaction mechanisms operate on timescales much faster than the cell cycle length.

To gain insights into the origins of tunability for the methylation compaction model, we adopt an approach, where we reduce this problem to using the Fokker-Planck approach, as utilized to analyze the methylation read-write mechanism (Fig. S3D,E). The full system with both methylation and compaction reactions would correspond to diffusive motion of a particle in a three-dimensional state space describing both chemical and physical states of nucleosomes. However, to simplify this problem to gain intuition, we will first take the methylation and demethylation reactions to be fast compared to the compaction and de-compaction reactions, such that the system can be described a single parameter C_T, corresponding to the total number of compacted nucleosomes. At any given time, the number of methylated and demethylated nucleosomes in the compacted state is at quasi-steady state, with values:

$$C' = \frac{\beta}{\beta + f\alpha} \cdot C_T \qquad (24)$$

and

$$C = \frac{f\alpha}{\beta + f\alpha} \cdot C_T \qquad (25)$$

Similarly, assuming that the system is at quasi steady state, the number of methylated and demethylated nucleosomes in the uncompacted state is given by:

$$D' = \frac{\beta}{\beta + \alpha} \cdot D_T \qquad (26)$$

and

$$D = \frac{\alpha}{\beta + \alpha} \cdot D_T \qquad (27)$$

Let $N_T = C_T + D_T$. With this approximation, the averaged rate of adding or removing a nucleosome from the compacted assembly is then given by:

$$k_{add} = k_2 + k_4 = \lambda C_T^{\frac{2}{3}} \frac{\beta}{\beta + \alpha} \cdot D_T + F \cdot \lambda C_T^{\frac{2}{3}} \frac{\alpha}{\beta + \alpha} \cdot D_T = \left(\frac{\beta}{\alpha} + F\right) \lambda C_T^{\frac{2}{3}} \cdot \frac{N_T - C_T}{1 + \frac{\beta}{\alpha}} \qquad (28)$$

$$k_{remov} = k_1 + k_3 = \frac{\delta}{C_T^{\frac{1}{3}}} C' + \frac{\delta}{C_T^{\frac{1}{3}}} C = \delta C_T^{\frac{2}{3}} \qquad (29)$$

Let the total number of compacted nucleosomes C_T be x. By writing down the master equation for this system, and by further applying the Fokker-Planck approximation, as performed in (5) and (6) we then have:

$$\frac{\partial p(x,t)}{\partial t} = \frac{\partial}{\partial x}[v(x)p(x)] + \frac{1}{2} \cdot \frac{\partial^2}{\partial x^2}[D(x)p(x)] \qquad (30)$$

where:

$$v(x) = \delta x^{\frac{2}{3}} - \left(\frac{\beta}{\alpha} + F\right) \lambda x^{\frac{2}{3}} \cdot \frac{N_T - x}{1 + \frac{\beta}{\alpha}} \qquad (31)$$

$$D(x) = \delta x^{\frac{2}{3}} + \left(\frac{\beta}{\alpha} + F\right) \lambda x^{\frac{2}{3}} \cdot \frac{N_T - x}{1 + \frac{\beta}{\alpha}} \qquad (33)$$

As before, we define a potential energy for this system:

$$-\frac{dV}{dx} = v(x) \qquad (34)$$

The analytical solution for the potential energy $V(x)$ for the methylation compaction model is:

$$V(x) = \frac{3}{5}\left[\delta - \frac{N}{1 + \frac{\beta}{\alpha}}\left(\frac{\beta}{\alpha} + F\right)\lambda\right] x^{\frac{5}{3}} + \frac{3}{8} \cdot \frac{1}{1 + \frac{\beta}{\alpha}}\left(\frac{\beta}{\alpha} + F\right)\lambda x^{\frac{8}{3}} + W \qquad (35)$$

A plot of $V(x)$ is demonstrated in Fig. S3D-E. We found that increasing methylation rate results in a much more attenuated increase in activation energy E_a with the methylation compaction model. This confirms that the improved switching rate tunability in the MC model stems from the decreased sensitivity to changes in activation barrier height by methylation rate. This result intuitive explains why this system shows significantly more graded changes in switching times when methylation rates are changed.

This tunability of switching times with respect to histone methylation depends on the relative association strengths of demethylated and methylated nucleosomes for each other in forming a compacted assembly. In our initial simulations, demethylated nucleosomes show only a moderate decrease in affinity for other compacted nucleosomes relative to methylated nucleosome (*F = 0.85*). However, when the binding strength of a demethylated nucleosome is much weaker than that of a methylated nucleosome (*F = 0.2*), we find changes in potential well heights become more significant, indicating that the system loses its tunability with respect to methylation changes (see Fig. S6E-D). This prediction, that methylated and demethylated nucleosomes have comparable strengths of association for a compacted assembly agrees well with evidence that unmethylated nucleosomes can nonetheless aggregate through a variety of H3K27me-independent mechanisms (Larson et al., 2017; Strom et al., 2017).

Model II.1: The Methylation Compaction Mechanism, with Compaction Disrupted by Division (Fig. S5)

This version of the model includes modified cellular division process in which upon replication, 50% of methylated nucleosomes become demethylated and 10% of compacted nucleosomes become uncompacted. This exit of nucleosomes from a compacted assembly due to DNA replication reflects the possibility that as the DNA replication machinery enters the compacted nucleosomal structure, it creates decompaction 'defects' in the condensed locus because nucleosomes near the replication forks are replaced. However, we reason that such defect would have a small effect to the overall stability of the structure because, at any given time, the site of replication would only take up a small region of the entire compacted domain.

In order to simulate both changes in methylation and compaction at the point of DNA replication, we must describe probabilistically how each of the four nucleosomal species are affected: 1) The Compacted-Methylated species (C'); 2) the Compacted-Demethylated species (C); 3) the Decompacted-Methylated species (D'); and 4) the Compacted-Demethylated species (D). Since methylation state is reduced by 50%, approximately half of Decompacted-Methylated species is transferred to Decompacted-Demethylated pool. Similarly, on average, 10% of the Compacted-Demethylated species are transferred to Decompacted-Demethylated pool due to DNA replication. Compacted-Methylated species have 50% chance to demethylate and 10% chance to decompact. Assuming these are two independent processes, this species has 5% chance to convert into Decompacted-Demethylated or Decompacted-Methylated and 45% chance to become Compacted-Demethylated. These observations are implemented as follows:

Let vector $S = [S_1, S_2, S_3, \dots, S_n]$ be the result from sampling a multinomial distribution with probabilities $\pi_1, \pi_2, \pi_3, \dots, \pi_n$, where $\pi_1 + \pi_2 + \pi_3 + \cdots + \pi_n = 1$. Let $S_i(\pi_1, \pi_2, \pi_3, \dots, \pi_n)$ be the ith element of S and N be the sample size. Let c', c, d', d be the number of compacted-methylated, compacted-unmethylated, decompacted-methylated, and decompacted-unmethylated nucleosomes, respectively immediately preceding the cellular division event. Partitioning of each species occurs as follows:

$$C' = S_4(0.45, 0.05, 0.05, 0.45) \quad (36)$$

$$D' = S_1(0.5, 0.5) + S_2(0.45, 0.05, 0.05, 0.45) \quad (37)$$

$$C = S_2(0.1, 0.9) + S_1(0.45, 0.05, 0.05, 0.45) \quad (38)$$

$$D = d + S_2(0.5, 0.5) + S_1(0.1, 0.9) + S_3(0.45, 0.05, 0.05, 0.45) \quad (39)$$

From stochastic simulations (Fig. S5A-E), we find that this modified methylation compaction model shows similar dynamic characteristics compared to the original methylation compaction model (Model II): it shows stochastic, all-or-none switching between inactive and active states; has an activation delay that can be tuned by changing H3K27me levels and enzyme activity; and shows division-independence in its activation time delay. Thus, we conclude that the essential features of

this model hold, even upon mild disruption of the inactive, compacted assembly by passage of DNA polymerase.

Model II.2: The Methylation Compaction Mechanism with Cooperative Methylation (Fig. S4)

PRC2 is known to be allosterically activated by H3K27me3 binding via its EED subunit (Margueron et al., 2009). Here, we consider this cooperative property of PRC2 by specifying that methylation rate increases with the total number of methylated nucleosomes in the model system. This assumption is likely valid when the number of nucleosomes in the condensed structure is small, and all the nucleosomes are more or less in close proximity with each other. To simulate this, we modified the methylation rates K_6 and K_8 so that their magnitude has a spontaneous term μ and the cooperative term β that is proportional to the total number of methylated species in the simulation:

$$C' \underset{k_6^*}{\overset{k_5}{\rightleftharpoons}} C \qquad\qquad D' \underset{k_8^*}{\overset{k_7}{\rightleftharpoons}} D$$

$$k_6{}^* = [\mu + \beta(C' + D')]C \qquad (40)$$

$$k_8{}^* = [\mu + \beta(C' + D')]D \qquad (41)$$

From stochastic simulations (Fig. S4), we find that this system is also capable of generating long, stochastic delays in all-or-none switching in locus compaction state, and that switching times can be finely tuned by changing H3K27me3 levels, as with our simpler methylation compaction model (Model II). We conclude that incorporation of a cooperative H3K27me3 methylation rate in our methylation compaction model does not alter its main conclusions.

Model II.3: Transcription factor tuning in the methylation-compaction model (Fig. 5)

Model II.3.1. Transcription factors prevent chromatin compaction

Here, we first consider a scenario where the transcription factors prevent a small number of nucleosomes from compaction. This can be accomplished by designating a small portion of nucleosomes to carry binding sites for the transcription factors. Upon binding to these factors, these nucleosomes can no longer associate with other nucleosomes in the compacted assembly:

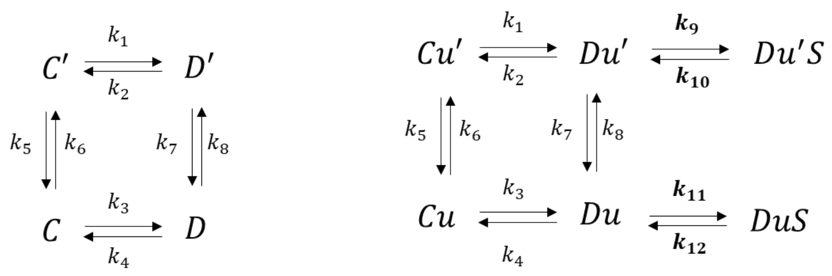

The nucleosomes carrying transcription factor binding sites are labeled *Cu, Cu', Du,* and *Du'*. Reaction rates $k_{1\text{-}8}$ are the same as the standard model. When these nucleosomes are decompacted, they can bind to the transcription factors with the following rates:

$$k_9 = K_{ON} \cdot TF \cdot Du' \qquad (42)$$

$$k_{10} = K_{OFF} \cdot Du'S \qquad (43)$$

$$k_{11} = K_{ON} \cdot TF \cdot Du \qquad (44)$$

$$k_{12} = K_{OFF} \cdot DuS \qquad (45)$$

Here $[TF]$ is the transcription factor concentration. Let C_T be the total number of compacted nucleosomes and let N_B be number of nucleosomes that carry transcription factor binding sites. We run our simulation with the assumption that the total number of nucleosomes is constant, and N_B can be varied.

From simulations (Fig. 5A), we find that activation timing can be tuned with the transcription factor copy number. Additionally, activation rate increases synergistically with number of binding sites. Therefore, timing modulation can be achieved via transcription factor acting as nucleosome sequester.

Model II.3.2. Transcription factor induce histone demethylation

We next consider an alternative mechanism where a transcription factor is recruited to the nucleosome assembly at a single site. Once bound, the transcription factor acts as a demethylase to removes methyl marks on the nucleosomes in its vicinity:

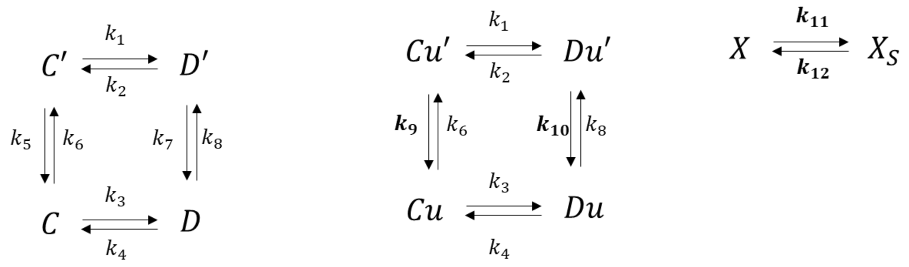

Here *Cu, Cu', Du,* and *Du'* are nucleosomes that are in the recruited demethylase's reach. The unrecruited demethylase is denoted as *X*, and X_S is the recruited transcription factor–bound demethylase. Since the simulation involves one nucleosomal array, the number of demethylases is limited to 1, such that $X + X_S = 1$. Conversion rate probability between *X* and X_S and the demethylation rate of the nucleosomes inside the reach of the methylase are described below:

$$k_9 = \alpha_{TF} \cdot X \cdot f \cdot Cu' \qquad (46)$$

$$k_{10} = \alpha_{TF} \cdot X_S \cdot Du' \qquad (47)$$

$$k_{11} = K_{ON} \cdot TF \cdot X \qquad (48)$$

$$k_{12} = K_{OFF} \cdot X_S \qquad (49)$$

Here, $\alpha_{TF}$is the transcription factor recruited demethylation rate constant, and *f* is the fraction reduction of the rate when the nucleosome is decompacted. In our simulations, C_T is the total number of compacted nucleosomes, as before, and N_R is the maximum number of nucleosomes that can be affected by the recruited demethylase. We run our simulation varying N_R, while keeping the total number of nucleosomes is constant. From simulations, we find that, unlike transcription factors that impact chromatin compaction, transcription factors that recruit demethylase according to this mechanism requires a large effective range to appreciably tune switching rate (Figure 5D).

Parameter List

a. Methylation Read-Write Model (Figure 4 A-B)

Pure Methylation Model			
**Parameters**	**Description**	**Value**	**Comments/references**
β_{ON}	cooperative methylation rate constant	1 hrs^{-1}	Sneeringer et al., 2010
α_{OFF}	demethylation rate constant	16.5 hrs^{-1}	[16 , 17 , 17.5] for parameter scan<br>Kristensen et al., 2011
L	reach of anchored methylating enzyme to neighboring nucleosome	15	Hass et al., 2015
N	number of simulated nucleosomes	100	
cell division length	cell division length	20 hrs	

b. Compaction Methylation Model (Figure 4 C-D)

Compaction Methylation Model			
**Parameters**	**Description**	**Value**	**Comments/references**
β	methylation rate constant	1 hrs^{-1}	[0.2 , 0.4 , 0.8 , 1.6 , 3.2 , 6.4] for parameter scan<br>Sneeringer et al., 2010
α	demethylation rate constant	8 hrs^{-1}	Kristensen et al., 2011
f	fraction of de-methylation rate when nucleosome is in compacted state	0	
λ	compaction rate constant	310 hrs^{-1}	Larson et al., 2017; Ladoux et al., 2000
δ	decompaction rate constant	5300 hrs^{-1}	Larson et al., 2017; Ladoux et al., 2000
F	fraction of compaction rate when nucleosome is in demethylated stated	0.85	
N	Number of simulated nucleosomes	50	
C_N	nucleation threshold for compacted nucleosomal complex	5	
cell division length	cell division length	20 hrs	

c. Cell division dependence of Pure Dilution vs Compaction Methylation Models (Figure 4 F-G)

Pure Dilution Model			
**Parameters**	**Description**	**Value**	**Comments/references**
β_{ON}	cooperative methylation rate constant	0	
α_{OFF}	demethylation rate constant	0	
L	reach of anchored methylating enzyme to neighboring nucleosome	0	
N	number of simulated nucleosomes	100	
cell division length	cell division length	20 hrs	[10 , 15 , 20 , 25 , 30 , 35] for cycle scan
**Compaction Methylation Model**			
**Parameters**	**Description**	**Value**	**Comments/references**
β	methylation rate constant	1 hrs^{-1}	Sneeringer et al., 2010
α	demethylation rate constant	8 hrs^{-1}	Kristensen et al., 2011
f	fraction of de-methylation rate when nucleosome is in compacted state	0	
λ	compaction rate constant	310 hrs^{-1}	
δ	decompaction rate constant	5300 hrs^{-1}	
F	fraction of compaction rate when nucleosome is in demethylated stated	0.85	
N	Number of simulated nucleosomes	50	
C_N	nucleation threshold for compacted nucleosomal complex	5	
cell division length	cell division length	20 hrs	[10 , 15 , 20 , 25 , 30 , 35] for cycle scan

d. Effects of Transcription Factors on Methylation Compaction Model's Activation Timing (Figure 5)

Transcription Factor Affecting Compaction Model			
Parameters	Description	Value	Comments/references
β	methylation rate constant	6.4 hrs^{-1}	Sneeringer et al., 2010
α	demethylation rate constant	8 hrs^{-1}	Kristensen et al., 2011
f	fraction of de-methylation rate when nucleosome is in compacted state	0	
λ	compaction rate constant	315 hrs^{-1}	Larson et al., 2017; Ladoux et al., 2000
δ	decompaction rate constant	5300 hrs^{-1}	Larson et al., 2017; Ladoux et al., 2000
F	fraction of compaction rate when nucleosome is in demethylated stated	0.85	
N	number of simulated nucleosomes	50	
C_N	nucleation threshold for compacted nucleosomal complex	5	
K_{ON}	transcription factor association constant	1 hrs-1	
K_{OFF}	transcription factor dissociation constant	5 hrs-1	
TF	transcription factor concentration	0-100	
N_B	total number of nucleosomes carrying transcription factor binding sites	1-5	
cell division length	cell division length	20 hrs	
**Compaction Methylation Model With Permanently demethylated Nucleosomes**			
Parameters	Description	Value	Comments/references
β	methylation rate constant	6.4 hrs^{-1}	Sneeringer et al., 2010
α	demethylation rate constant	8 hrs^{-1}	Kristensen et al., 2011
f	fraction of de-methylation rate when nucleosome is in compacted state	0	
λ	compaction rate constant	295 hrs^{-1}	Larson et al., 2017; Ladoux et al., 2000
δ	decompaction rate constant	5300 hrs^{-1}	Larson et al., 2017; Ladoux et al., 2000
F	fraction of compaction rate when nucleosome is in demethylated stated	0.85	
N	Total number of simulated nucleosomes	50	
C_N	nucleation threshold for compacted nucleosomal complex	5	
K_{ON}	transcription factor association constant	1 hrs-1	
K_{OFF}	transcription factor dissociation constant	5 hrs-1	
TF	transcription factor concentration	0-35	
N_B	total number of nucleosomes carrying transcription factor binding sites	5-25	
α_{TF}	recruited demethylase rate constant	1600 hrs-1	
cell division length	cell division length	20 hrs	

e. Methylation model with different cooperative reach parameter L (Supplementary Figure 3A-B)

Pure Methylation Model			
Parameters	Description	units	values
β_{ON}	cooperative methylation rate constant	hrs^{-1}	1
α_{OFF}	demethylation rate constant	hrs^{-1}	[2, 2.3, 3] for L = 3
			[2.8, 3, 4] for L = 4
			[9, 10, 11] for L = 5
			[10, 10.5, 11] for L = 10
L	reach of anchored methylating enzyme to neighboring nucleosome	nucleosomes	[3, 4, 5 ,10]
N	number of simulated nucleosomes	nucleosomes	100
cell division length	cell division length	hrs	20

f. Potential energy landscapes analysis for pure methylation model and methylation compaction model (Supplementary Figure 3C - E)

Pure Dilution Model		
Parameters	Description	Value
β	cooperative methylation rate constant	1-4 hrs^{-1}
α	demethylation rate constant	20 hrs^{-1}
N	number of simulated nucleosomes	100
W	arbitrary constant	100
**Compaction Methylation Model**		
Parameters	Description	Value
β	methylation rate constant	1-4 hrs^{-1}
α	demethylation rate constant	20 hrs^{-1}
λ	compaction rate constant	31 hrs^{-1}
δ	decompaction rate constant	530 hrs^{-1}
F	fraction of compaction rate when nucleosome is in demethylated stated	0.85
N	Number of simulated nucleosomes	50

g. Compaction with Cooperative Methylation Model (Supplementary Figure 4)

Compaction Cooperative Methylation Model			
Parameters	Description	Value	Comments/references
β	cooperative methylation rate constant	0.02 hrs^{-1}	
μ	spontaneous rate constant	[0.05 , 0.1 , 0.2 , 0.4] hrs^{-1}	
α	demethylation rate constant	8 hrs^{-1}	Kristensen et al., 2011
f	fraction of de-methylation rate when nucleosome is in compacted state	0	
λ	compaction rate constant	310 hrs^{-1}	Larson et al., 2017; Ladoux et al., 2000
δ	decompaction rate constant	5300 hrs^{-1}	Larson et al., 2017; Ladoux et al., 2000
F	fraction of compaction rate when nucleosome is in demethylated stated	0.85	
N	number of simulated nucleosomes	50	
C_N	nucleation threshold for compacted nucleosomal complex	5	
cell division length	cell division length	20 hrs	
**Pure Methylation Model**			
Parameters	Description	Value	Comments
$β_{ON}$	cooperative methylation rate constant	1 hrs^{-1}	Sneeringer et al., 2010
$α_{OFF}$	demethylation rate constant	[16 , 17 , 17.5] hrs^{-1}	for parameter scan, Kristensen et al., 2011
L	reach of anchored methylating enzyme to neighboring nucleosome	15	Hass et al., 2015
N	number of simulated nucleosomes	100	
cell division length	cell division length	20 hrs	

h. Compaction Methylation Model with Compaction State Disruption by Cell Division (Supplementary Figure 5A-E)

Compaction Methylation Model with Compaction State Affected by Cell Division			
Parameters	Description	Value	Comments/references
β	methylation rate constant	1 hrs^{-1}	[0.2 , 0.4 , 0.8 , 1.6 , 3.2 , 6.4] for parameter scan in Supplemental Figure 2E<br>Sneeringer et al., 2010
α	demethylation rate constant	8 hrs^{-1}	Kristensen et al., 2011
f	fraction of de-methylation rate when nucleosome is in compacted state	0	
λ	compaction rate constant	310 hrs^{-1}	Larson et al., 2017; Ladoux et al., 2000
δ	decompaction rate constant	5300 hrs^{-1}	Larson et al., 2017; Ladoux et al., 2000
F	fraction of compaction rate when nucleosome is in demethylated stated	0.85	
N	Number of simulated nucleosomes	50	
C_N	nucleation threshold for compacted nucleosomal complex	5	
cell division length	cell division length	20 hrs	[10 , 20 , 25 , 30 , 35] for parameter scan in Supplemental Figure 2D

i. Pure Dilution Model with Minimal Methylation and Demethylation Rates (Supplementary Figure 5F)

Dilution Model With Minimal Enzymatic Activities			
Parameters	Description	Value	Comments/references
$β_{ON}$	cooperative methylation rate constant	0.001 hrs-1	
$α_{OFF}$	demethylation rate constant	0.001 hrs-1	
L	reach of anchored methylating enzyme to neighboring nucleosome	15	Hass et al., 2015
N	number of simulated nucleosomes	100	
cell division length	cell division length	10 and 20 hrs	
