# Supplementary material for: Tunable, division-independent control of gene activation timing by a polycomb switch
Source: Cell Rep. Author manuscript; Available in PMC 2021 Apr 7. (PMC8024876; doi:10.1016/j.celrep.2021.108888)
Supplement: 2 [file NIHMS1686913-supplement-2.pdf]

Report

Cell Reports

Tunable, division-independent control of gene activation timing by a polycomb switch

Graphical abstract

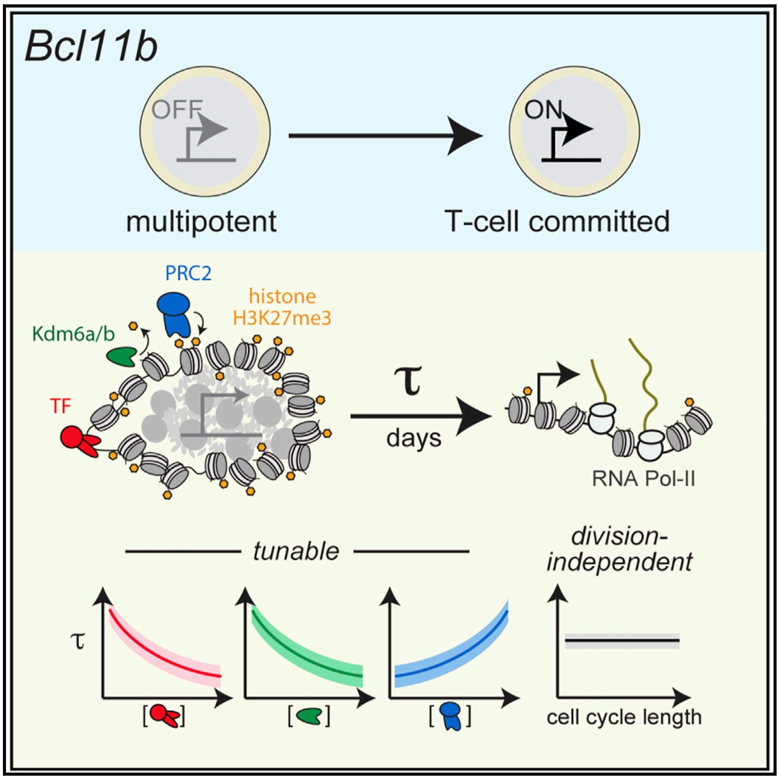

Authors

Nicholas A. Pease, Phuc H.B. Nguyen, Marcus A. Woodworth, Kenneth K.H. Ng, Blythe Irwin, Joshua C. Vaughan, Hao Yuan Kueh

Correspondence

kueh@uw.edu

In brief

H3K27me3 is found on repressed lineage-specifying gene loci in stem cells and may delay their activation in response to signals. Pease et al. identify an adjustable timer mechanism, where H3K27me3 levels at the T cell commitment gene *Bcl11b*, set by opposing writer and eraser enzymes, modulate the timing of gene locus decompaction and activation.

Highlights

- Timed epigenetic switch delays *Bcl11b* activation over multiple cell cycles
- H3K27me3 levels, set by PRC2 and Kdm6a/b, tunably set this activation delay
- This activation time delay is set independently from cell cycle length
- Quantitative methylation-compaction model explains tunable, division-independent delays

Pease et al., 2021, Cell Reports *34*, 108888
March 23, 2021 © 2021 The Author(s).
https://doi.org/10.1016/j.celrep.2021.108888

CellPress

Cell Reports

CellPress
OPEN ACCESS

Report

Tunable, division-independent control of gene activation timing by a polycomb switch

Nicholas A. Pease,[1,2,8] Phuc H.B. Nguyen,[1,3,8] Marcus A. Woodworth,[4,5] Kenneth K.H. Ng,[1] Blythe Irwin,[1] Joshua C. Vaughan,[5,6] and Hao Yuan Kueh[1,7,9,*]
[1]Department of Bioengineering, University of Washington, Seattle, WA 98195, USA
[2]Molecular and Cellular Biology Program, University of Washington, Seattle, WA 98195, USA
[3]Molecular Engineering and Sciences Institute, University of Washington, Seattle, WA 98195, USA
[4]Biological Physics, Structure and Design Program, University of Washington, Seattle, WA 98195, USA
[5]Department of Chemistry, University of Washington, Seattle, WA 98195, USA
[6]Department of Physiology and Biophysics, University of Washington, Seattle, WA 98195, USA
[7]Institute for Stem Cell and Regenerative Medicine, University of Washington, Seattle, WA 98195, USA
[8]These authors contributed equally
[9]Lead contact
*Correspondence: kueh@uw.edu
https://doi.org/10.1016/j.celrep.2021.108888

SUMMARY

During development, progenitors often differentiate many cell generations after receiving signals. These delays must be robust yet tunable for precise population size control. Polycomb repressive mechanisms, involving histone H3 lysine-27 trimethylation (H3K27me3), restrain the expression of lineage-specifying genes in progenitors and may delay their activation and ensuing differentiation. Here, we elucidate an epigenetic switch controlling the T cell commitment gene *Bcl11b* that holds its locus in a heritable inactive state for multiple cell generations before activation. Integrating experiments and modeling, we identify a mechanism where H3K27me3 levels at *Bcl11b*, regulated by methyltransferase and demethylase activities, set the time delay at which the locus switches from a compacted, silent state to an extended, active state. This activation delay robustly spans many cell generations, is tunable by chromatin modifiers and transcription factors, and is independent of cell division. With their regulatory flexibility, such timed epigenetic switches may broadly control timing in development.

INTRODUCTION

During multicellular development, stem and progenitor cells often differentiate many days and cell divisions after receiving instructive signals. These differentiation delays must be robust yet tunable over timescales spanning multiple cell generations for precise control over differentiated population sizes. In diverse contexts, cell differentiation delays are generated by timing mechanisms operating autonomously in single cells. In classic studies of oligodendrocyte differentiation, precursor cells exposed to signals delayed their differentiation by up to eight cell divisions, due to a cell-autonomous timing mechanism (Gao et al., 1997; Temple and Raff, 1986). Such autonomous timing control is seen in diverse systems, from brain and muscle development to adaptive immunity (Burton et al., 1999; Heinzel et al., 2017; Otani et al., 2016).

Although timing delays in some embryonic systems are generated by mechanisms that count cell divisions (Amodeo et al., 2015; Newport and Kirschner, 1982), differentiation delays during later vertebrate development or in adult stem cells are often uncoupled from cell cycle progression, such that changes to rates of cell division do not affect delay duration (Burton et al., 1999; Gao et al., 1997; Heinzel et al., 2017; Li et al., 2019; Okamoto et al., 2016; Osmond, 1991; Otani et al., 2016). A mechanism for setting the elapsed time to differentiation apart from cell division could provide functional advantages to cells, including operation in non-dividing cells and an ability to modulate cell expansion while maintaining a constant temporal schedule for differentiation. However, it is unknown how division-independent timing control is implemented on a molecular level.

Polycomb repressive mechanisms, involving histone H3 lysine-27 trimethylation (H3K27me3), are important for differentiation timing control. H3K27me3 modifications are often found at the loci of lineage-specifying genes in stem cells, where they restrain the expression of these genes and resultant differentiation (Boyer et al., 2006; Lee et al., 2006). During differentiation, instructive signals activate transcription factors that bind to lineage-specifying genes and initiate H3K27me3 removal. However, although transcription factors usually bind rapidly upon signal exposure within minutes, H3K27me3 loss and gene activation often occur much more slowly, such that gene loci can heritably maintain a silent state over multiple cell generations prior to activation (Berry et al., 2017; Kaikkonen et al., 2013; Bintu et al., 2016; Mayran et al., 2018). In a prevailing view, this epigenetic maintenance of the silent state before activation

This is an open access article under the CC BY license (http://creativecommons.org/licenses/by/4.0/).

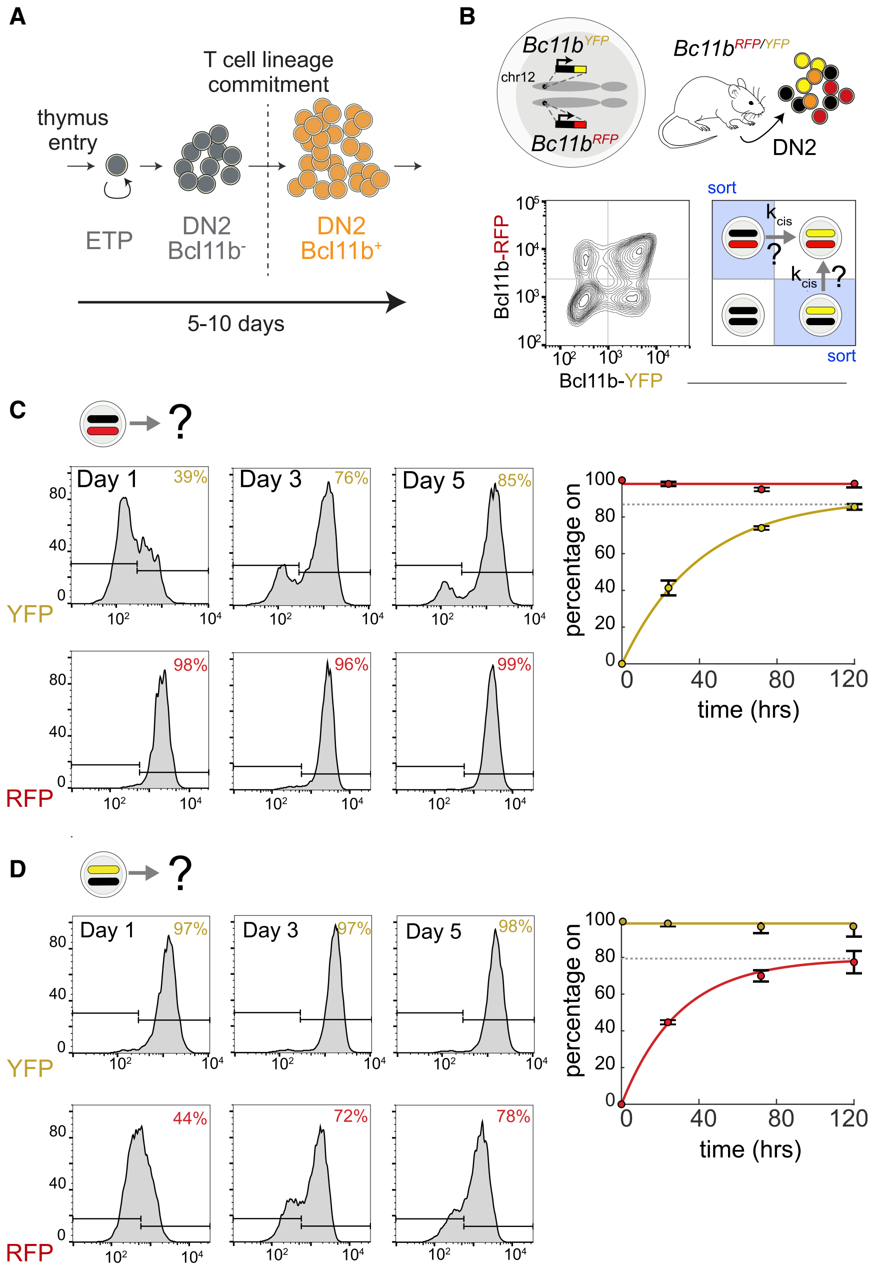

Figure 1. A timed epigenetic switch delays *Bcl11b* activation and T cell lineage commitment

(A) *Bcl11b*, a transcription factor that drives T cell lineage commitment, turns on with a multi-day time delay.

(B) Dual-allelic *Bcl11b* reporter mouse (top), along with flow cytometry plot showing levels of each *Bcl11b* allele in DN2 progenitors (bottom left) and with strategy to purify *Bcl11b* monoallelic expressing progenitors for live-cell analysis of epigenetic switch timing, k_{cis}.

(C and D) DN2 monoallelic *Bcl11b*-expressing progenitors were purified, cultured on OP9-DL1 feeders with 5 ng/mL interleukin-7 (IL-7) and Flt3L, and analyzed by flow cytometry.

Data represent means and 95% confidence intervals for n = 3 independent experiments. Curves represent fits to the equation $y = F(1 - e^{-kt})$, where F is the final percentage of cells positive for assayed allele (represented by the dotted gray lines); $k = 0.025\ h^{-1} \pm 0.005$ for YFP activation and $k = 0.034\ h^{-1} \pm 0.009$ for RFP activation.

results from the passive dilution of H3K27me3 modified histones through serial cell division (Gaydos et al., 2014; Jadhav et al., 2020; Sun et al., 2014). However, although passive dilution mechanisms can delay differentiation over multiple cell divisions, these delays would depend on cell cycle duration and be inconsistent with the division-independent timers found in vertebrates. Alternatively, H3K27me3 loss may be actively controlled by the opposing polycomb repressive complex 2 (PRC2) methyltransferase and Kdm6a/b demethylase activities (Li et al., 2014; Park et al., 2014; Seenundun et al., 2010). However, it is unclear whether such active mechanisms could generate differentiation delays spanning many cell divisions and whether these delays could be both tunable and cell-division independent.

To address these questions, we investigated the mechanism of a time-delayed epigenetic switch controlling the activation of *Bcl11b*, a transcription factor essential for T cell lineage commitment and identity (Hosokawa et al., 2018; Ikawa et al., 2010; Li et al., 2010; Figure 1A). In early T cell progenitors, Notch signals activate *Bcl11b*, both directly (Ikawa et al., 2010; Li et al., 2010) and indirectly, by activating its upstream regulators Gata3 and TCF-1 (García-Ojeda et al., 2013; Germar et al., 2011; Zhou et al., 2019). However, although these upstream regulators become active shortly after thymic entry, *Bcl11b* activation and T cell lineage commitment occur ~5–10 days later, during which progenitors proliferate 1,000-fold (Manesso et al., 2013; Porritt et al., 2003; Zhou et al., 2019). Using a dual-allele *Bcl11b* reporter strain, where each endogenous gene copy is tagged with distinguishable fluorescent protein reporters (Ng et al., 2018), we found that this long delay in *Bcl11b* activation arises partly because of an epigenetic switch acting at individual *Bcl11b* loci, in *cis*. This epigenetic switch activates probabilistically, with a multi-day time constant that is controlled by Gata3 and TCF-1 (Kueh et al., 2016), along with a distal enhancer to which these factors bind (Ng et al., 2018). However, our previous studies did not clarify the mechanism of the timed epigenetic switch itself or its basis for generating controllable timing delays in gene activation. We address these outstanding questions here.

CellPress
OPEN ACCESS

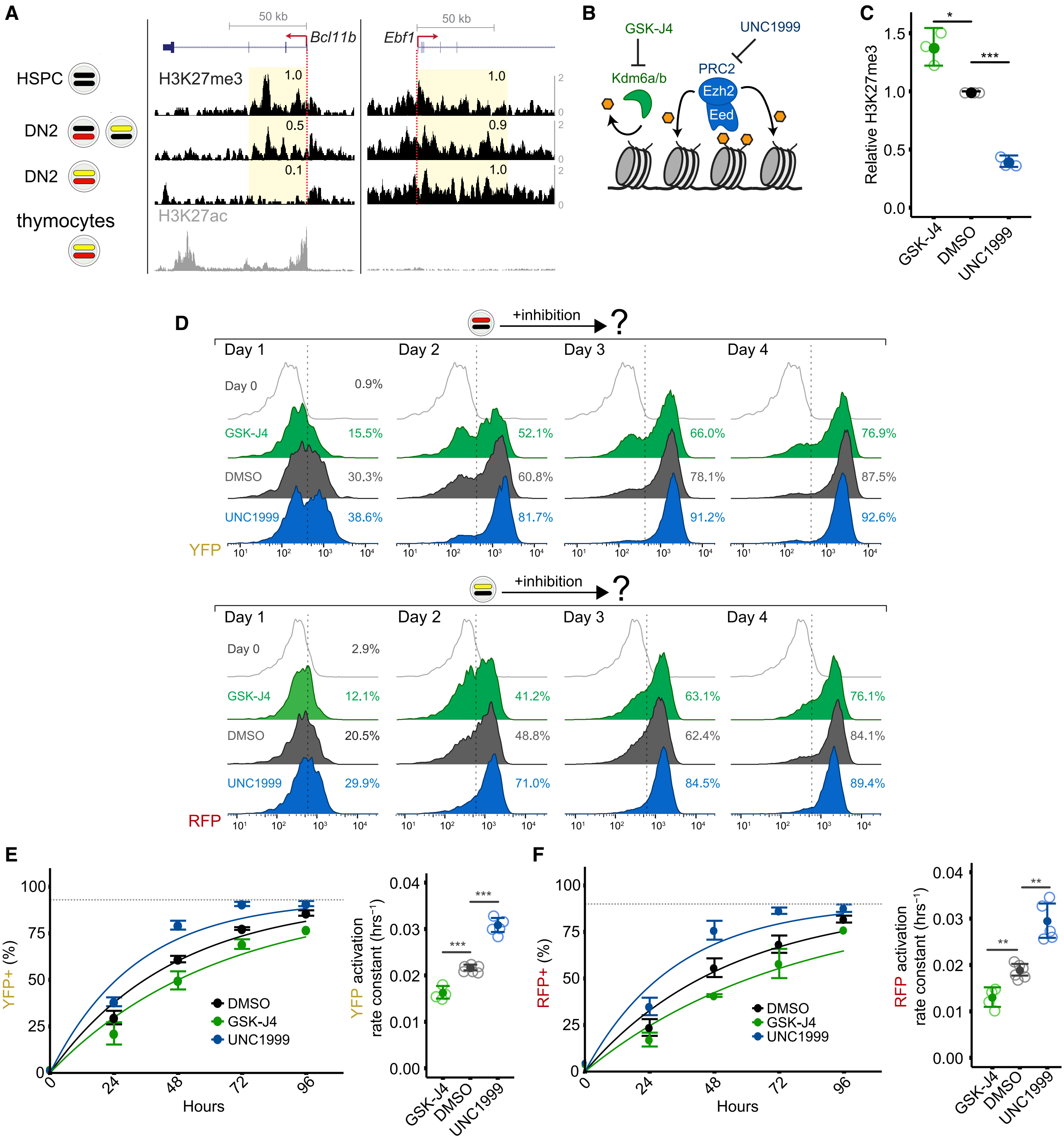

Figure 2. H3K27me3 levels, set by PRC2 and Kdm6a/b demethylases, modulate *Bcl11b* activation timing

(A) H3K27me3 distributions were profiled by CUT&RUN in Lin− bone marrow progenitors (hematopoietic stem and progenitor cells [HSPCs]), as well as purified DN2 monoallelic and biallelic *Bcl11b*-expressing cells with UCSC Genome Browser tracks showing H3K27me3 densities at *Bcl11b*, as well as at *Ebf1*, a B cell regulator that is repressed during T cell development. Relative read densities of shaded areas are shown. H3K27ac levels in thymocytes, obtained from ENCODE accession number ENCSR000CCH (Davis et al., 2018), demarcate transcribed region. Data are representative of two independent experiments.

(B) Schematic depicting inhibition of H3K27 demethylases Kdm6a/b or H3K27 methyltransferase PRC2.

(C) DN2 monoallelic progenitors treated with the indicated inhibitors were sorted for anti-H3K27me3 CUT&RUN followed by qPCR at the *Bcl11b* promoter. Mean values are shown for n = 3 independent experiments (two-sample t test, one-tailed: *$p < 0.05$; ***$p < 0.001$).

(D) Purified DN2 monoallelic expressing cells were re-cultured with the indicated inhibitors and analyzed by flow cytometry. Histograms show results from one representative experiment.

(legend continued on next page)

RESULTS

A timed epigenetic switch delays *Bcl11b* activation and T cell lineage commitment

To study the *cis*-acting epigenetic event controlling *Bcl11b* activation timing in isolation from other events occurring in *trans*, we analyzed *Bcl11b* locus activation dynamics in progenitors that already have one *Bcl11b* allele active and must therefore contain all *trans*-factors necessary for expression (Figure 1B). Using fluorescence-activated cell sorting (FACS), we purified monoallelic *Bcl11b* expressing DN2 progenitors from dual-allelic reporter mice and analyzed activation of the silent allele by co-culture with OP9-DL1 cells, an *in vitro* system that recapitulates early transitions in T cell development (Holmes and Zuniga-Pflucker, 2009). Inactive *Bcl11b* alleles turned on after a long time delay such that the fraction of biallelically expressing cells increased progressively over the course of 5 days (Figures 1C and 1D). Activation kinetics were similar for both YFP (mCitrine yellow fluorescent protein) and RFP (mCherry red fluorescent protein) alleles and were well described by a single exponential curve, consistent with activation being controlled by a single stochastic event occurring with equal likelihood at each allele.

H3K27me3 levels at the *Bcl11b* locus tune activation timing

The repressive histone modification H3K27me3 is highly enriched at silent *Bcl11b* loci in hematopoietic progenitor cells, but not in committed T cells where *Bcl11b* is expressed (Zhang et al., 2012). Therefore, H3K27me3 removal could regulate the epigenetic event controlling *Bcl11b* activation timing. To test this possibility, we first determined whether H3K27me3 marks are removed from the *Bcl11b* locus at the same time it turns on. To pinpoint when H3K27me3 loss occurs relative to locus activation, we measured H3K27me3 levels in three progenitor populations having different numbers of active *Bcl11b* loci. In bone marrow progenitors, where both *Bcl11b* alleles are inactive, there was an abundance of H3K27me3 across the 5′ end of *Bcl11b* (Figure 2A). These broad H3K27me3 peaks were roughly halved in monoallelic *Bcl11b* expressing DN2 progenitors and were almost completely absent in biallelic *Bcl11b* DN2 progenitors (Figure 2A). These results show H3K27me3 is lost from the *Bcl11b* locus concurrently with its activation.

H3K27me3 may modulate the timing of *Bcl11b* activation; alternatively, its loss may simply be a consequence of gene activation due to clearance of methylated nucleosomes by active transcription (Hosogane et al., 2016; Kraushaar et al., 2013). To determine whether H3K27me3 modifications play a causal role in controlling *Bcl11b* activation timing, we cultured monoallelic *Bcl11b* expressing DN2 progenitors with small-molecule inhibitors targeting H3K27me3-modifying enzymes and analyzed the effects on activation of the silent *Bcl11b* allele. These inhibitors, which target either the PRC2 methyltransferase subunit Ezh2 (UNC1999) or the H3K27 demethylases Kdm6a/b (GSK-J4; Figure 2B), resulted in an ~60% decrease and ~40% increase, respectively, in H3K27me3 abundance at the *Bcl11b* promoter in monoallelic *Bcl11b* expressing DN2 progenitors (Figure 2C), indicating that they actively modulate H3K27me3 levels at inactive *Bcl11b* loci.

To determine whether H3K27me3 levels regulate *Bcl11b* activation timing, we assayed the expression of inactive *Bcl11b* alleles in DN2 progenitors over the course of 4 days. In the absence of any inhibitors, the silent *Bcl11b* alleles in monoallelic progenitors activated at an average rate of 0.022 h^{-1} and 0.019 h^{-1} for YFP and RFP alleles, respectively (Figures 2D–2F). Kdm6a/b demethylase inhibition decreased the activation rate for each silent allele in a graded manner (27%–37%). Conversely, Ezh2 inhibition increased the activation rate for each silent allele (41%–58%). Similar graded decreases or increases in *Bcl11b* activation probabilities were observed for another structurally unrelated Kdm6a/b inhibitor, IOX-1, and other Ezh2 inhibitors, GSK-126 and GSK-343 (Figure S1A). Furthermore, short-hairpin-RNA-mediated knockdown of another essential PRC2 subunit, *Eed*, also increased the activation rate of silent *Bcl11b* alleles (Figures S1A, S1C, and S1D), arguing against non-specific pharmacological effects. Notably, all H3K27me3 perturbations tested altered the fraction of activated cells without altering expression levels in activated cells (Figure S1A), indicating a specific role of these modifications in tuning gene activation timing. Importantly, perturbing H3K27 methylation rates had no effect on the apoptosis frequency among different populations, indicating that these changes in activation fractions are not due to differential cell death in these populations (Figure S1E). Taken together, these results show that H3K27me3 levels at the *Bcl11b* locus, set by opposing PRC2 methyltransferase and Kdm6a/b demethylase activities, control the timing of *Bcl11b* activation.

Bcl11b activation timing is regulated independently of cell division

Activation delays of polycomb-repressed genes have been proposed to result from the passive dilution of H3K27me3 modified histones with cell division (Coleman and Struhl, 2017; Jiang and Berger, 2017; Strome et al., 2014; Sun et al., 2014). However, the observed regulation of *Bcl11b* activation timing by H3K27 demethylases that counteract PRC2-mediated methylation (Figure 2) suggests an active mechanism is involved that could potentially operate independently of cell division. To determine whether *Bcl11b* activation timing depends on cell division, we accelerated the cell division rate in DN2 RFP+/YFP− progenitors by transducing them with the proto-oncogene c-Myc and used quantitative live-cell imaging to measure the activation kinetics of the silent YFP allele (Figure 3A). As expected, c-Myc overexpression resulted in an ~2-fold increase in the cell division rate (Figure 3B; see also Figure S2, Table S1, and Quantitative and statistical analysis). However, despite accelerating cell division,

(E and F) (Left) Mean activation percentages and 95% confidence intervals are plotted with curves representing fits to the equation $y = F(1 - e^{-kt})$, where F = maximum percentage of cells positive for assayed allele (represented by the dotted gray lines). (Right) Data represent mean rate constants, k, with 95% confidence intervals (two-sample t test, one-tailed: $^{**}p < 0.01$; $^{***}p < 0.001$; n = 4–6 independent experiments).
See also Figure S1.

CellPress
OPEN ACCESS

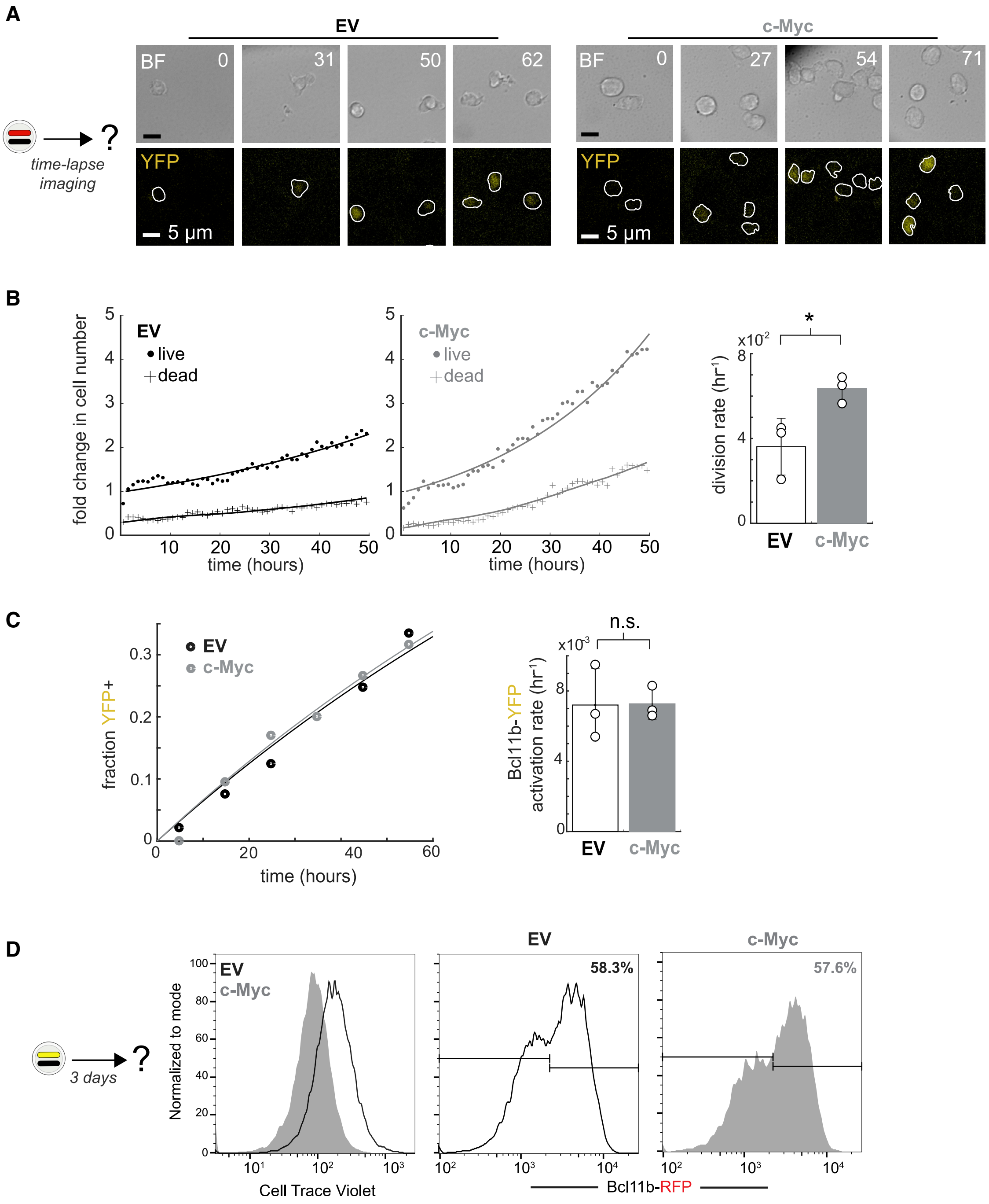

Figure 3. *Bcl11b* activation timing is independent of cell-division speed

Bcl11b$^{YFP-/RFP+}$ DN2 progenitors transduced with either an empty vector (EV) or c-Myc overexpressing retroviral vector were purified, re-cultured on DL1-coated plates, and monitored by time-lapse imaging.

(A) Time-lapse images. White boundaries show automated cell segmentation. Numbers show elapsed time in hours.

(B) (Left) Time evolution of live and dead cell numbers. Data were fitted to a population dynamics model as shown in Figure S2. (Right) Data represent mean and standard deviation of cell division rates for n = 3 independent experiments (paired two-sample t test, one-tailed; *$p < 0.025$).

(C) (Left) Fraction of YFP+ cells over time. (Right) Data represent mean and standard deviation of *Bcl11b-YFP* activation rates for n = 3 independent experiments (paired two-sample t test, one-tailed; n.s., not significant).

(D) Bcl11b$^{RFP+/YFP-}$ DN2 progenitors transduced with either EV or c-Myc were re-cultured on OP9-DL1 stromal monolayers for 3 days before analyzing by flow cytometry.

See also Figure S2 and Table S1.

c-Myc overexpression did not change *Bcl11b* activation timing, with control and accelerated progenitors activating the silent YFP allele with the same exponential time constant (~136 h; Figure 3C). This lack of change in activation timing following cell division acceleration was also observed by flow cytometry for silent RFP alleles (Figure 3D). Therefore, in contrast to the passive dilution paradigm, these results show that the epigenetic switch controlling *Bcl11b* activation generates time delays in gene activation that are independent of cell division.

A methylation-compaction mechanism for tunable, division-independent timing control

The molecular mechanism underlying the timed *Bcl11b* epigenetic switch must account for its observed emergent properties, namely, (1) its ability to robustly set time delays that span multiple cell generations, (2) its stochastic nature, (3) its tunability by histone-modifying enzyme activities, and (4) its cell division independence. To identify mechanisms with these emergent properties, we used mathematical modeling to analyze a series of candidate mechanisms. H3K27me3 can bind PRC2 at an allosteric site and activate its methyltransferase activity (Margueron et al., 2009), allowing these modifications to spread to neighboring nucleosomes and be maintained across cell division. Previous work has shown that such positive feedback, mediated by a read-write mechanism for histone methylation, can generate bistable switching that occurs over timescales spanning many cell generations (Dodd et al., 2007; Zhang et al., 2014). Therefore, we first analyzed a simple model of this positive feedback mechanism. In this methylation read-write (M) model, the *Bcl11b* locus comprises a linear array of N nucleosomes, each of which can be methylated or demethylated (Figure 4A; see Methods S1). Each nucleosome is methylated at a rate that increases with the number of nearby methylated nucleosomes and is demethylated at a first-order rate. During DNA replication, nucleosomes randomly segregate to the two daughter strands, resulting in half of the methylated nucleosomes being replaced by demethylated ones (Coleman and Struhl, 2017).

Our simulations revealed that single-nucleosome arrays could switch from a repressed H3K27 methylated state to a demethylated state with stochastic time delays spanning multiple cell divisions (Figure 4A, center), consistent with previous work (Dodd et al., 2007; Zhang et al., 2014). However, in our simulations, activation timing was extremely sensitive to H3K27 methylation levels in the silent state, with minor changes in methylation levels (~10%) causing dramatic changes activation timing (~300-fold; Figure 4B). This extreme sensitivity far exceeded the sensitivity coefficient observed experimentally (Figures 2C–2F and 4B) across a wide range of methyltransferase reach lengths (Figures S3A and S3B) and was also seen in other studies (Dodd et al., 2007; Zhang et al., 2014), indicating that it represents a general feature of positive-feedback-mediated switching and not a specific aspect of our model. By analyzing this system using a transition state theory framework (Figures S3C–S3E; see also Methods S1), we found that switching times scale exponentially with methylation or demethylation rates, thus explaining the observed extreme sensitivity. Thus, models that consider histone modification dynamics alone are inconsistent with the tunable control of activation by H3K27me3-modifying enzymes observed experimentally (Figures 2C–2F).

H3K27me3 modifications repress gene expression by promoting the formation of compacted chromatin assemblies inaccessible to the transcription machinery. Compaction may occur through H3K27me3-dependent recruitment of PRC1, which can self-associate through intrinsically disordered domains on its Cbx2 subunit to promote chromatin condensation (Plys et al., 2019; Tatavosian et al., 2019); alternatively, compaction may involve direct interactions between nucleosomes that are modulated by the H3K27me3 state (Gibson et al., 2019; Sanulli et al., 2019). In both cases, weak multivalent interactions between nucleosomes drive chromatin condensation through phase separation.

In light of these recent findings, we developed a second model, where H3K27me3 does not directly repress transcription per se but instead enhances the strength of nucleosomal interactions to keep the locus compacted and restrain its activation (Figure 4C; see Methods S1). In this methylation-compaction (MC) model, the *Bcl11b* locus consists of an ensemble of nucleosomes that can exist in methylated or demethylated states and can be included in or excluded from a compacted nucleosome assembly. Methylation and demethylation of nucleosomes occur with first-order rates, whereas association and dissociation of nucleosomes with the compacted chromatin assembly occur at rates proportional to its surface area, which we assume scales with the two-thirds power of the number of nucleosomes in the assembly C_T. This rate dependency assumes that nucleosomes enter (or exit) the compacted assembly only by formation (or breakage) of weak multivalent interactions with nucleosomes on the assembly surface. Methylation and compaction are coupled such that the methylation state of a nucleosome affects its rate of association with a compacted assembly and vice versa. Below a threshold number of nucleosomes, the chromatin assembly is unstable and dissolves, leading to locus decompaction and consequent gene expression. This stability threshold reflects a minimum nucleus size needed to maintain a phase-separated condensate.

Simulations of the methylation-compaction model revealed that the gene locus can maintain a H3K27 methylated and compacted state for multiple cell divisions before switching in an all-or-none manner to a decompacted, low-methylation state (Figure 4C, center). As with the methylation-only model, the time delay in switching is well described by a first-order stochastic process, with a constant probability of activation per unit time (Figure 4D). However, in contrast with the methylation read-write model but in concordance with our experimental results (Figure 2), changing H3K27me3 levels by varying methylation or demethylation rates changed gene activation timing in a much more graded manner (Figures 4B and 4E). This tunability was robust over different parameter ranges (Figure 4B, top right), different degrees of cooperativity for H3K27 methylation (Figure S4), and different degrees of assembly disruption after DNA replication (Figures S5A–S5E). A transition state theory analysis (see Methods S1 and Figures S3C–S3E) showed that, in order for gene activation timing to be finely tunable, nucleosomes must be able to associate with each other even without H3K27me3 modifications, such that methylated and

CellPress
OPEN ACCESS

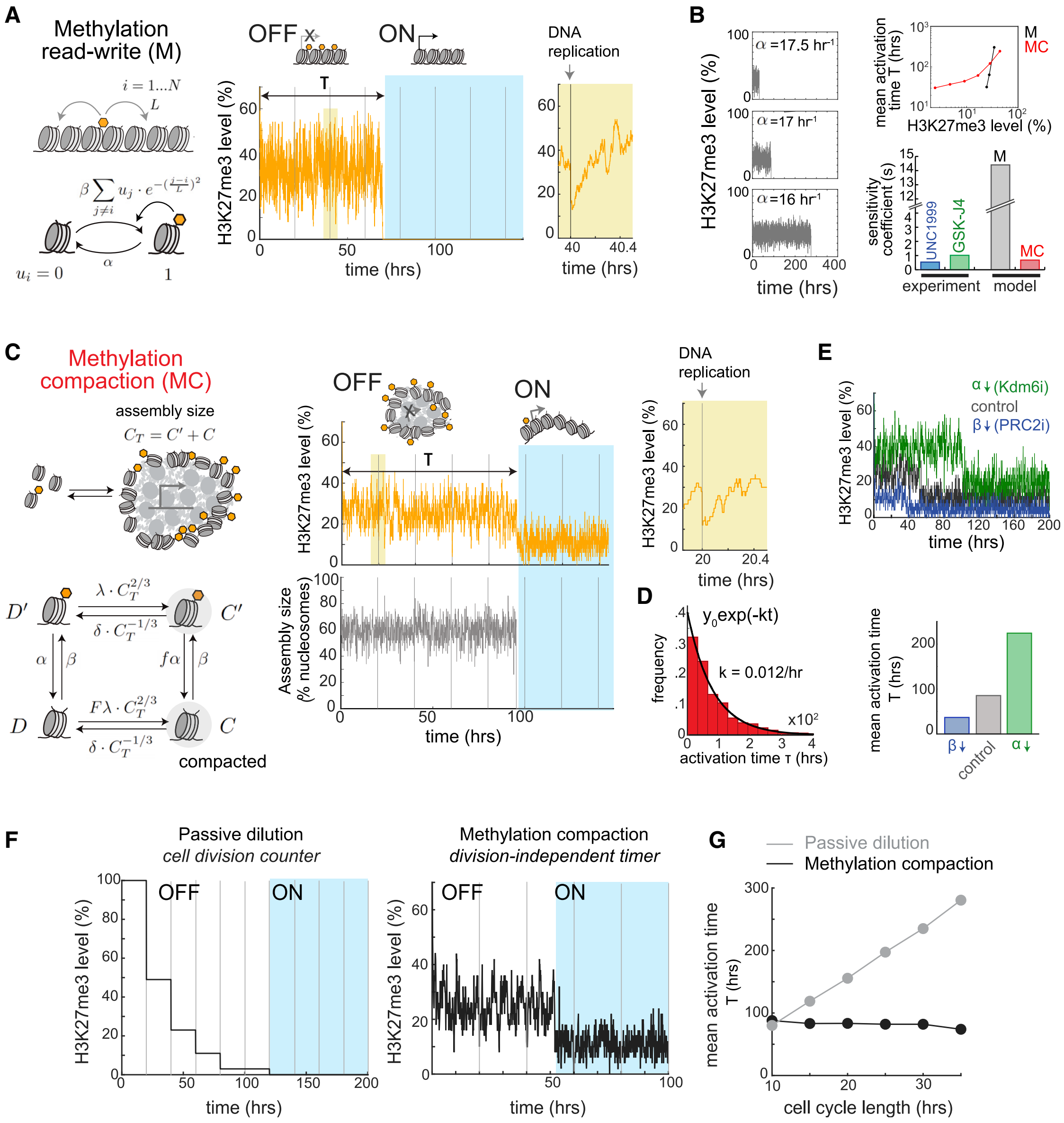

Figure 4. A methylation-compaction switching mechanism generates tunable, division-independent delays in gene activation
(A) Methylation read-write (M) model (left), along with representative simulation (right).
(B) Representative simulations of M model with different demethylation rates α (left). Mean activation times against H3K27me3 levels (top right) and sensitivity coefficients for this relationship (bottom right) are shown.
(C) Methylation compaction (MC) model (left), along with representative simulation (right).
(D) Histogram shows distribution of activation times, along with exponential fit.
(E) Representative simulations of MC model, simulating PRC2 or Kdm6a/b inhibition (top), along with mean activation times (bottom).
(F) Simulations of passive dilution and MC models. Vertical lines indicate DNA replication events.
(G) Mean activation times as a function of cell cycle length.
See also Figures S3–S7.

demethylated nucleosomes can associate with each other with comparable affinities. Consistent with this idea, there are multiple mechanisms for nucleosomal interactions that work independently of H3K27me3 modifications (Francis et al., 2004; Gibson et al., 2019; Larson et al., 2017; Sanulli et al., 2019; Strom et al., 2017).

As cell division rate did not affect timing delays in *Bcl11b* activation (Figure 3), we tested whether the methylation-compaction mechanism also generates cell-division-independent activation delays. Indeed, in contrast to a passive dilution model for H3K27me3 loss (Figure 4F), the methylation-compaction model generated activation delays that were constant over a range of

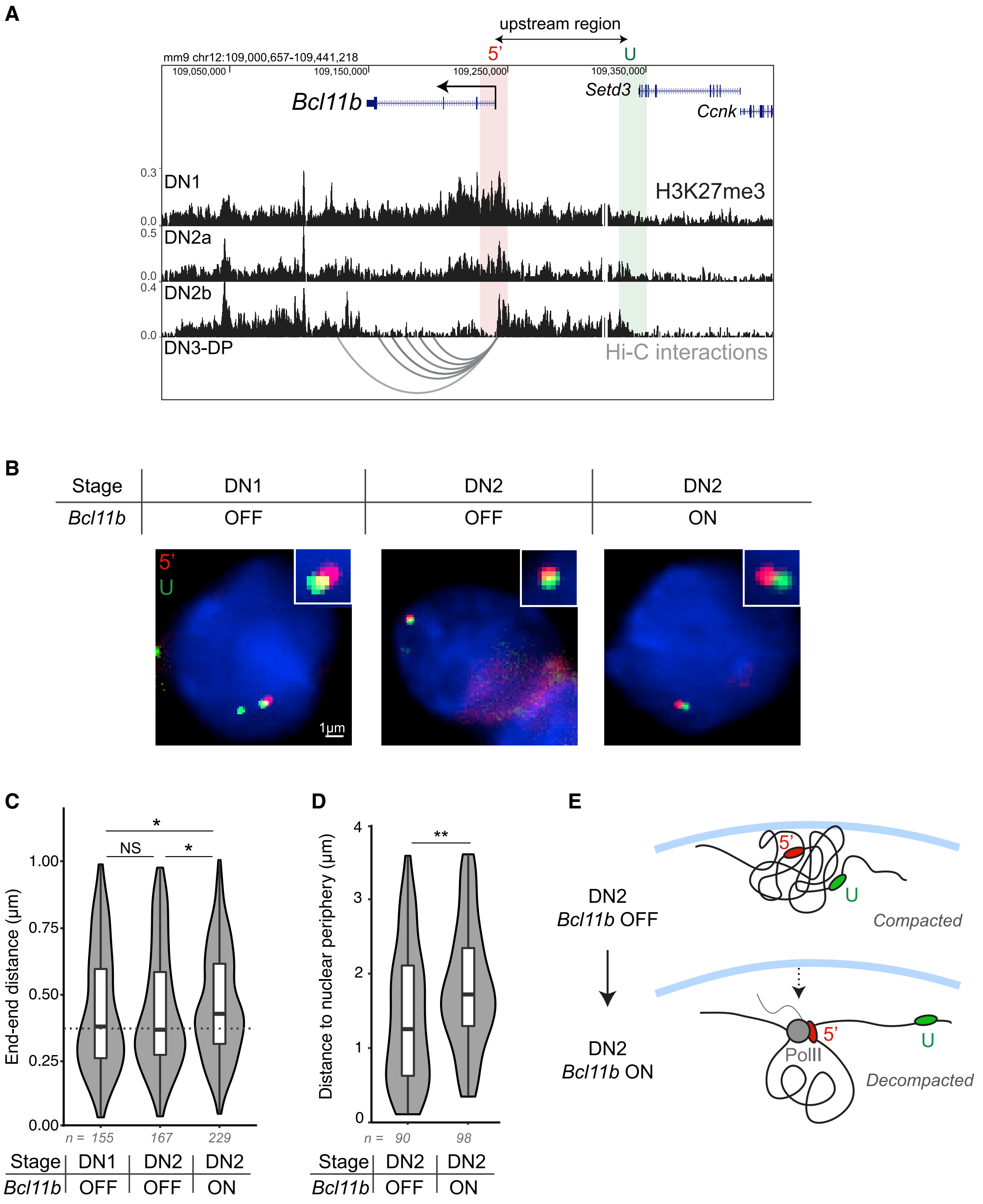

Figure 5. The *Bcl11b* locus switches to an extended conformation with activation

(A) UCSC Genome Browser view of H3K27me3 chromatin immunoprecipitation sequencing (ChIP-seq) results in T cell progenitors (Zhang et al., 2012) and Hi-C representation maps of interactions between the *Bcl11b* transcription start site and other DNaseI hypersensitivity sites in Bcl11b+ DN3-DP T cell progenitors (Hu et al., 2018).

(B) Representative images for each condition. Centroids for each foci pair shown exist in the same *z*-plane and thus provide visual representation of the Euclidean distance.

(C) Violin plots show the results from 3D Euclidean distance measurements between each probe pair (Mann Whitney U test significance: $^*p < 0.05$; n = number of foci pairs). T cell progenitors were sorted based on cell surface markers and *Bcl11b* reporter expression (either *Bcl11b* OFF [RFP−/YFP−] or *Bcl11b* ON [RFP+/YFP+]) before performing DNA-FISH with upstream end-end probes above.

(legend continued on next page)

cell cycle speeds (Figures 4G and S5A–S5E). We note that this cell division independence was lost when histone methylation and demethylation rates were reduced to be slower than the cell division rates (Figure S5F), implying a need for rapid histone methylation dynamics for upholding cell cycle independence. Indeed, histone methylation and nucleosome compaction dynamics occur with reported timescales of minutes and seconds (Kristensen et al., 2011; Larson et al., 2017; Sneeringer et al., 2010), respectively, which are far faster than the typical cell division rates which range from hours to days. This explains why the epigenetic state recovers rapidly after DNA replication as observed in our simulations (Figures 4A and 4B, right).

These modeling results suggest that timing control by H3K27me3 has the following characteristics: first, H3K27me3 loss does not directly result in gene activation but instead modulates a separate process that acts as the gatekeeper for gene transcription. Given recent insights into how nucleosomes can interact to form phase-separated structures (Gibson et al., 2019), and how these interactions can be modulated by histone tail modifications and/or binding proteins (Larson et al., 2017; Plys et al., 2019; Strom et al., 2017; Tatavosian et al., 2019), we propose that H3K27me3 loss weakens nucleosomal interactions at the *Bcl11b* locus, promoting gene locus decompaction and gene activation. Second, the compaction process itself must be partially independent of H3K27me3 such that the compacted nucleosome assembly can be maintained by other chromatin-associated proteins (Francis et al., 2004; Larson et al., 2017; Strome et al., 2014).

The *Bcl11b* locus switches to an extended conformation with activation

The methylation-compaction mechanism above assumes that the *Bcl11b* locus is compacted prior to activation but switches to an extended, decompacted state during activation. To validate this assumption, we measured the end-to-end distances between genomic regions at the *Bcl11b* locus using DNA fluorescence *in situ* hybridization (FISH), an established approach to estimate the degree of chromatin compaction at a gene locus (Eskeland et al., 2010; Giorgetti et al., 2015). To estimate the degree of compaction independently from RNA polymerase II (RNAPII)-mediated DNA looping at the *Bcl11b* locus (Hu et al., 2018; Zheng et al., 2019), we designed a pair of FISH probe sets that flank a 100-kb region upstream of the promoter. This upstream region begins at the *Bcl11b* promoter and resides at the edge of the putative heterochromatin compaction domain that encompasses *Bcl11b* (Figure 5A). We performed DNA-FISH in early T cell progenitors (DN1) and DN2 progenitors before or after *Bcl11b* activation (RFP−/YFP− versus RFP+/YFP+) and measured end-to-end distances using three-dimensional imaging.

These experiments showed that the *Bcl11b* locus showed similar end-to-end distances in RFP−/YFP− DN1 and DN2 progenitors; however, in RFP+/YFP+ DN2 progenitors, where *Bcl11b* first turns on, this end-to-end distance increased significantly, consistent with this locus maintaining a condensed state through early T cell development but switching abruptly to an extended state during *Bcl11b* activation (Figures 5B and 5C). Repressed heterochromatic regions of the genome frequently reside at the nuclear periphery, where interactions between nuclear lamina proteins and nucleosomes are thought to facilitate chromatin compaction (van Steensel and Belmont, 2017; Ulianov et al., 2019). Therefore, we examined whether *Bcl11b* moves from the nuclear periphery to the interior as it decompacts and turns on, as suggested from previous studies (Isoda et al., 2017). Indeed, the distance between the *Bcl11b* promoter and the nuclear periphery was higher in *Bc111b*-expressing DN2 progenitors compared to *Bcl11b*-non-expressing progenitors at the same DN2 stage, consistent with a transition from the nuclear periphery to the interior upon gene activation (Figure 5D). This finding, in conjunction with the observed increase in the end-end distance of the *Bcl11b* locus, suggests that *Bcl11b* transitions from a condensed heterochromatin-associated state to an accessible euchromatin-associated state during transcriptional activation (Figure 5E), consistent with the methylation-compaction model.

H3K27me3-independent regulation of *Bcl11b* epigenetic switch timing

The methylation-compaction model predicts that, in order for activation time delays to be tunable, nucleosomes must retain an ability to interact independently of H3K27me3. H3K9me2/3 is also associated with repressive heterochromatin domains and can serve as a binding site for HP1α, which can facilitate nucleosome adhesion in the absence of H3K27me3 (Poleshko et al., 2013; Sanulli et al., 2019; Wang et al., 2019). H3K9me3 is enriched at the *Bcl11b* locus in non-T-cell lineages (Figure S6A); therefore, we tested whether H3K9 methylation also regulates *Bcl11b* activation timing. To do so, we sorted DN2 progenitors with one active *Bcl11b* allele, re-cultured them with inhibitors targeting H3K9me3-modifying enzymes, and quantified the *Bcl11b* activation state after 3 days. We found that inhibition of Lsd1, an H3K9 demethylase, decreased the fraction of biallelic *Bcl11b*-expressing cells, whereas inhibition of G9a, an H3K9 methyltransferase, increased the fraction of biallelic *Bcl11b*-expressing cells (Figures S6B and S6C). The dose-dependent yet moderate degree to which H3K9 methylation perturbations altered *Bcl11b* activation rate was similar to that for H3K27 methylation perturbations, as seen in Figure 2D. Thus, consistent with the methylation-compaction model, multiple histone modifications work together to modulate *Bcl11b* activation timing, conceivably by working together to modulate nucleosomal interactions at the gene locus.

(D) Violin plots show the results from 2D measurements between the *Bcl11b* promoter and the nuclear periphery (Mann Whitney U test significance: **$p < 0.01$; n = number of foci).

(E) Schematic depicting decompaction model. In the OFF state, *Bcl11b* exists in a compacted conformation residing at the nuclear lamina. In the ON state, the locus moves away from the nuclear periphery and becomes decompacted, resulting in increased distance between the promoter, 5′, and the upstream, U, regions.

Transcription factors can tune gene activation time delays

Bcl11b activation timing is tunable not only by chromatin-modifying enzymes, as shown above (Figure 2), but also by two transcription factors, Gata3 and TCF-1, through a distal enhancer that physically interacts with the *Bcl11b* promoter before activation (Hu et al., 2018; Isoda et al., 2017; Kueh et al., 2016; Ng et al., 2018). These findings are consistent with broader literature showing that transcription factors and their binding sequences can modulate target gene activation probabilities (Dufourt et al., 2018; Walters et al., 1995; Weintraub, 1988), though it remains unknown how they achieve such tunable timing control. Here, we tested whether the methylation-compaction mechanism could integrate information about transcription factor levels to control activation timing. We first considered a scheme, where transcription factors bind to nucleosomes and block their association with other nucleosomes in the compacted assembly (Figure S7A). Indeed, both Gata3 and TCF-1 have been identified as pioneer factors, which possess affinity to nucleosomes in addition to specific DNA sequences (Fernandez Garcia et al., 2019; Johnson et al., 2018; Meers et al., 2019; Zaret and Carroll, 2011). Such disruption of compaction could also occur via the activation of gene or non-coding RNA transcription (Rinn et al., 2007; Tu et al., 2017) or by recruitment of factors that disrupt interactions between nucleosomes (Kraushaar et al., 2013; Talbert and Henikoff, 2017; Zhou et al., 2016).

Our simulations revealed that the rate constant for gene activation varied with both transcription factor concentration and the number of transcription factor binding sites (Figure S7B). Increasing the number of bound nucleosomes N_B increased the activation rate in a synergistic manner, with a progressive increase in the maximal activation rate with addition of each binding site (Figure S7B, top right). Interestingly, increasing the number of binding sites also increased the transcription factor concentration at which half-maximal activation occurred (Figure S7B, bottom right). These findings suggest that transcription factors can tunably control the timing of gene activation by blocking internucleosomal interactions and chromatin compaction.

Besides blocking nucleosomal interactions, transcription factors may also promote activation by inducing histone demethylation. Histone demethylation could occur by direct recruitment of Kdm6a/b demethylases (Estarás et al., 2013; Seenundun et al., 2010; Williams et al., 2014) or through PRC2 eviction by chromatin-remodeling enzymes (Kadoch et al., 2017). Therefore, we considered a second mechanism, where transcription factors induce the demethylation of N_R nucleosomes around their binding vicinities (Figure S7C). Our simulations revealed that transcription factors could modulate activation timing, as above, but only if they could induce demethylation of a large number of nucleosomes in their binding vicinity (Figure S7D). When a smaller number of nucleosomes were impacted, activation rates shifted only slightly when transcription factor levels increased (Figure S7D). Consistent with this idea, transcription factors bound to a single binding site can often induce chromatin changes over a significant area spanning many nucleosomes (Hass et al., 2015; Heinz et al., 2010).

DISCUSSION

During development, progenitor cells differentiate, with time delays spanning multiple days and cell generations. These delays are often independent of cell division and are tunably controlled by regulatory inputs. Here, we elucidated the mechanism of a time-delayed epigenetic switch controlling the activation of the T cell specifying gene *Bcl11b* and developed a mathematical model to explain its emergent properties. We show that H3K27me3 levels at the *Bcl11b* locus, set by opposing methyltransferase and demethylase activities, modulate the *Bcl11b* activation time delay by controlling its switch from a compacted, silent state to an extended, actively expressing state. Activation delays generated by this methylation-compaction mechanism robustly span multiple cell generations, can be tunably modulated by both histone-modifying enzymes and transcription factors, and are set independently from cell division.

In contrast to previous epigenetic switching models, which only consider the dynamics of histone modification (Dodd et al., 2007; Zhang et al., 2014), the methylation-compaction model we propose couples histone methylation to nucleosomal interactions. Consistent with this idea, both H3K27me3 and H3K9me2/3 can promote nucleosomal self-association though histone-tail interactions (Gibson et al., 2019) or by recruiting protein complexes that self-associate to form phase-separated condensates (Plys et al., 2018; Sanulli et al., 2019; Tatavosian et al., 2019; Wang et al., 2019). Importantly, in order for the activation times to be tunable, nucleosomes must retain some self-interaction affinity without H3K27me3, such that methylation promotes but is not strictly necessary for nucleosomal association. The concept that modification states of proteins modulate their interaction affinities is well established in the study of cytoskeletal polymers (Howard, 2001; Mitchison, 1992; Phillips et al., 2012) but could provide a fresh perspective on the relationship between chromatin modifications and chromatin structure. Further testing of the methylation-compaction model will require direct interrogation of chromatin states at individual gene loci in single cells, work that will be aided by new methods to simultaneously visualize histone modification states and chromatin folding at single gene loci in single cells (Kundu et al., 2017; Xu et al., 2018; Woodworth et al., 2020).

The methylation-compaction switching mechanism could underlie diverse cell-autonomous timers that have been observed to work independently of cell division (Burton et al., 1999; Gao et al., 1997; Heinzel et al., 2017; Li et al., 2019; Okamoto et al., 2016; Osmond, 1991; Otani et al., 2016). Measuring elapsed time independently of cell division could enable unique functions, including operation in non-dividing cells and constancy amid changes to cell proliferation, which could allow for tunable population size control, an idea we explore in a separate study (Nguyen et al., 2019). Our simulations revealed that such division-independent timing control requires active turnover of H3K27me3 and nucleosome compaction dynamics to be rapid compared to the cell cycle length (Methods S1; Figure S5F). We currently lack methods to measure H3K27me3 turnover kinetics at specific genomic loci *in vivo*; however, the active roles of PRC2 and Kdm6a/b demethylases in modulating H3K27me3 levels at the *Bcl11b* locus (Figure 2), their fast catalysis rates

(Kristensen et al., 2011; Sneeringer et al., 2010), along with the observation that nucleosomes within polycomb domains are replaced with kinetics much faster than that of cell division (1.5 h versus 20 h; Deal et al., 2010) suggest that H3K27me3 indeed turns over at a much faster timescale than that of cell division.

Cell type specification during multicellular development is controlled by gene-regulatory networks whose dynamics unfold over timescales spanning many cell generations. The division-independent timed epigenetic switch we describe here is uniquely tunable at multiple levels of gene regulation, including histone modifications, transcription factors, and non-coding *cis*-regulatory elements. Thus, it could serve as a modular building block for gene-regulatory networks that enables robust, adjustable control of developmental timing as well as organism size and form.

STAR★METHODS

Detailed methods are provided in the online version of this paper and include the following:

- KEY RESOURCES TABLE
- RESOURCE AVAILABILITY
  - Lead contact
  - Materials availability
  - Data and code availability
- EXPERIMENTAL MODEL AND SUBJECT DETAILS
  - Animal Models
  - Cell Line Culture
- METHOD DETAILS
  - Cell purification
  - *In vitro* differentiation of T cell progenitors
  - Flow cytometry and cell sorting
  - Retroviral construct and transduction
  - CUT&RUN H3K27me3 profiling
  - CUT&RUN library preparation and sequencing
  - CUT&RUN sequencing analysis
  - CUT&RUN qPCR
  - Cell preparation for time-lapse imaging
  - Oligopaint DNA-FISH
  - FISH imaging and analysis
- QUANTIFICATION AND STATISTICAL ANALYSIS
  - Modeling simulations
  - Image analysis of time-lapse movies
  - Population dynamics model and fitting

SUPPLEMENTAL INFORMATION

Supplemental information can be found online at https://doi.org/10.1016/j.celrep.2021.108888.

ACKNOWLEDGMENTS

We thank members of the Kueh lab for discussions and also thank Steve Henikoff, James Briscoe, Ellen Rothenberg, Michael Elowitz, and Joe Levine for discussions and feedback on this work. We also thank Steve Henikoff for the generous gift of pA-MNase for CUT&RUN experiments. This work was funded by a Pathway to Independence Award 5R00HL119638 (to H.Y.K.) from the National Institutes of Health from the United States; grant R01HL146478 (to H.Y.K.) from the National Heart, Lung, and Blood Institute of the United States; URoL EF-2021552 (to H.Y.K.) from the National Science Foundation of the United States; a Stem Cell Scientist Award (to H.Y.K.) from the Tietze Foundation of the United States; a fellowship (to N.A.P.) from the University of Washington Institute of Stem Cell and Regenerative Medicine of the United States; and F31 Fellowships F31 HL151090 (to N.A.P.) and F31 HL142132 (to M.A.W.) from the National Heart, Lung, and Blood Institute of the United States.

AUTHOR CONTRIBUTIONS

P.H.B.N., N.A.P., and H.Y.K. conceived the study and designed the experiments. P.H.B.N. and N.A.P. performed all the experiments and analyzed the data. P.H.B.N. and H.Y.K. conceived and analyzed the mathematical models. P.H.B.N. performed stochastic simulations of the mathematical models. B.I. performed analysis on imaging data. K.K.H.N. generated reporter mice for this study. M.A.W., N.A.P., and J.C.V. designed and executed the FISH experiments. P.H.B.N., N.A.P., and H.Y.K. wrote the manuscript.

DECLARATION OF INTERESTS

The authors declare no competing interests.

Received: August 7, 2019
Revised: December 17, 2020
Accepted: March 1, 2021
Published: March 23, 2021

SUPPORTING CITATIONS

The following references appear in the supplemental information: Agger et al. (2007); Alabert et al. (2015); Angel et al. (2011); Arbona et al. (2017); Erickson and Pantaloni (1981); Gray et al. (2016); Isono et al. (2013); Jackson and Berkowitz (1980); Kahn et al. (2016); Kramers (1940); Ladoux et al. (2000); MacPherson et al. (2018); Nuebler et al. (2018).

REFERENCES

Agger, K., Cloos, P.A.C., Christensen, J., Pasini, D., Rose, S., Rappsilber, J., Issaeva, I., Canaani, E., Salcini, A.E., and Helin, K. (2007). UTX and JMJD3 are histone H3K27 demethylases involved in HOX gene regulation and development. Nature *449*, 731–734.

Alabert, C., Barth, T.K., Reverón-Gómez, N., Sidoli, S., Schmidt, A., Jensen, O.N., Imhof, A., and Groth, A. (2015). Two distinct modes for propagation of histone PTMs across the cell cycle. Genes Dev. *29*, 585–590.

Amodeo, A.A., Jukam, D., Straight, A.F., and Skotheim, J.M. (2015). Histone titration against the genome sets the DNA-to-cytoplasm threshold for the *Xenopus* midblastula transition. Proc. Natl. Acad. Sci. USA *112*, E1086–E1095.

Angel, A., Song, J., Dean, C., and Howard, M. (2011). A polycomb-based switch underlying quantitative epigenetic memory. Nature *476*, 105–108.

Arbona, J.-M., Herbert, S., Fabre, E., and Zimmer, C. (2017). Inferring the physical properties of yeast chromatin through Bayesian analysis of whole nucleus simulations. Genome Biol. *18*, 81.

Arganda-Carreras, I., Sorzano, C.O.S., Marabini, R., Carazo, J.M., Ortiz-de-Solorzano, C., and Kybic, J. (2006). Consistent and elastic registration of histological sections using vector-spline regularization. In Computer Vision Approaches to Medical Image Analysis, R.R. Beichel and M. Sonka, eds. (Springer Berlin Heidelberg), pp. 85–95.

Beliveau, B.J., Boettiger, A.N., Nir, G., Bintu, B., Yin, P., Zhuang, X., and Wu, C.-T. (2017). In situ super-resolution imaging of genomic DNA with OligoSTORM and OligoDNA-PAINT. Methods Mol. Biol. *1663*, 231–252.

Beliveau, B.J., Kishi, J.Y., Nir, G., Sasaki, H.M., Saka, S.K., Nguyen, S.C., Wu, C.T., and Yin, P. (2018). OligoMiner provides a rapid, flexible environment for the design of genome-scale oligonucleotide in situ hybridization probes. Proc. Natl. Acad. Sci. USA *115*, E2183–E2192.

Berry, S., Dean, C., and Howard, M. (2017). Slow chromatin dynamics allow polycomb target genes to filter fluctuations in transcription factor activity. Cell Syst. *4*, 445–457.e8.

Bintu, L., Yong, J., Antebi, Y.E., McCue, K., Kazuki, Y., Uno, N., Oshimura, M., and Elowitz, M.B. (2016). Dynamics of epigenetic regulation at the single-cell level. Science *351*, 720–724.

Boyer, L.A., Plath, K., Zeitlinger, J., Brambrink, T., Medeiros, L.A., Lee, T.I., Levine, S.S., Wernig, M., Tajonar, A., Ray, M.K., et al. (2006). Polycomb complexes repress developmental regulators in murine embryonic stem cells. Nature *441*, 349–353.

Burton, P.B.J., Raff, M.C., Kerr, P., Yacoub, M.H., and Barton, P.J.R. (1999). An intrinsic timer that controls cell-cycle withdrawal in cultured cardiac myocytes. Dev. Biol. *216*, 659–670.

Choi, K., Medley, J.K., König, M., Stocking, K., Smith, L., Gu, S., and Sauro, H.M. (2018). Tellurium: an extensible python-based modeling environment for systems and synthetic biology. Biosystems *171*, 74–79.

Coleman, R.T., and Struhl, G. (2017). Causal role for inheritance of H3K27me3 in maintaining the OFF state of a *Drosophila* HOX gene. Science *356*, eaai8236.

Davis, C.A., Hitz, B.C., Sloan, C.A., Chan, E.T., Davidson, J.M., Gabdank, I., Hilton, J.A., Jain, K., Baymuradov, U.K., Narayanan, A.K., et al. (2018). The Encyclopedia of DNA elements (ENCODE): data portal update. Nucleic Acids Res. *46* (*D1*), D794–D801.

Deal, R.B., Henikoff, J.G., and Henikoff, S. (2010). Genome-wide kinetics of nucleosome turnover determined by metabolic labeling of histones. Science *328*, 1161–1164.

Dodd, I.B., Micheelsen, M.A., Sneppen, K., and Thon, G. (2007). Theoretical analysis of epigenetic cell memory by nucleosome modification. Cell *129*, 813–822.

Dufourt, J., Trullo, A., Hunter, J., Fernandez, C., Lazaro, J., Dejean, M., Morales, L., Nait-Amer, S., Schulz, K.N., Harrison, M.M., et al. (2018). Temporal control of gene expression by the pioneer factor Zelda through transient interactions in hubs. Nat. Commun. *9*, 5194.

Erickson, H.P., and Pantaloni, D. (1981). The role of subunit entropy in cooperative assembly. Nucleation of microtubules and other two-dimensional polymers. Biophys. J. *34*, 293–309.

Eskeland, R., Leeb, M., Grimes, G.R., Kress, C., Boyle, S., Sproul, D., Gilbert, N., Fan, Y., Skoultchi, A.I., Wutz, A., and Bickmore, W.A. (2010). Ring1B compacts chromatin structure and represses gene expression independent of histone ubiquitination. Mol. Cell *38*, 452–464.

Estarás, C., Fueyo, R., Akizu, N., Beltrán, S., and Martínez-Balbás, M.A. (2013). RNA polymerase II progression through H3K27me3-enriched gene bodies requires JMJD3 histone demethylase. Mol. Biol. Cell *24*, 351–360.

Fellmann, C., Hoffmann, T., Sridhar, V., Hopfgartner, B., Muhar, M., Roth, M., Lai, D.Y., Barbosa, I.A.M., Kwon, J.S., Guan, Y., et al. (2013). An optimized microRNA backbone for effective single-copy RNAi. Cell Rep. *5*, 1704–1713.

Fernandez Garcia, M., Moore, C.D., Schulz, K.N., Alberto, O., Donague, G., Harrison, M.M., Zhu, H., and Zaret, K.S. (2019). Structural features of transcription factors associating with nucleosome binding. Mol. Cell *75*, 921–932.e6.

Francis, N.J., Kingston, R.E., and Woodcock, C.L. (2004). Chromatin compaction by a polycomb group protein complex. Science *306*, 1574–1577.

Gao, F.-B., Durand, B., and Raff, M. (1997). Oligodendrocyte precursor cells count time but not cell divisions before differentiation. Curr. Biol. *7*, 152–155.

García-Ojeda, M.E., Klein Wolterink, R.G.J., Lemaître, F., Richard-Le Goff, O., Hasan, M., Hendriks, R.W., Cumano, A., and Di Santo, J.P. (2013). GATA-3 promotes T-cell specification by repressing B-cell potential in pro-T cells in mice. Blood *121*, 1749–1759.

Gaydos, L.J., Wang, W., and Strome, S. (2014). Gene repression. H3K27me and PRC2 transmit a memory of repression across generations and during development. Science *345*, 1515–1518.

Germar, K., Dose, M., Konstantinou, T., Zhang, J., Wang, H., Lobry, C., Arnett, K.L., Blacklow, S.C., Aifantis, I., Aster, J.C., and Gounari, F. (2011). T-cell factor 1 is a gatekeeper for T-cell specification in response to Notch signaling. Proc. Natl. Acad. Sci. USA *108*, 20060–20065.

Gibson, B.A., Doolittle, L.K., Schneider, M.W.G., Jensen, L.E., Gamarra, N., Henry, L., Gerlich, D.W., Redding, S., and Rosen, M.K. (2019). Organization of chromatin by intrinsic and regulated phase separation. Cell *179*, 470–484.e21.

Giorgetti, L., Piolot, T., and Heard, E. (2015). High-resolution 3D DNA FISH using plasmid probes and computational correction of optical aberrations to study chromatin structure at the sub-megabase scale. In Nuclear Bodies and Noncoding RNAs, S. Nakagawa and T. Hirose, eds. (Springer New York), pp. 37–53.

Gray, F., Cho, H.J., Shukla, S., He, S., Harris, A., Boytsov, B., Jaremko, Ł., Jaremko, M., Demeler, B., Lawlor, E.R., et al. (2016). BMI1 regulates PRC1 architecture and activity through homo- and hetero-oligomerization. Nat. Commun. *7*, 13343.

Hass, M.R., Liow, H.-H., Chen, X., Sharma, A., Inoue, Y.U., Inoue, T., Reeb, A., Martens, A., Fulbright, M., Raju, S., et al. (2015). SpDamID: marking DNA bound by protein complexes identifies Notch-dimer responsive enhancers. Mol. Cell *59*, 685–697.

Heinz, S., Benner, C., Spann, N., Bertolino, E., Lin, Y.C., Laslo, P., Cheng, J.X., Murre, C., Singh, H., and Glass, C.K. (2010). Simple combinations of lineage-determining transcription factors prime cis-regulatory elements required for macrophage and B cell identities. Mol. Cell *38*, 576–589.

Heinzel, S., Binh Giang, T., Kan, A., Marchingo, J.M., Lye, B.K., Corcoran, L.M., and Hodgkin, P.D. (2017). A Myc-dependent division timer complements a cell-death timer to regulate T cell and B cell responses. Nat. Immunol. *18*, 96–103.

Holmes, R., and Zuniga-Pflucker, J.C. (2009). The OP9-DL1 system: generation of T-lymphocytes from embryonic or hematopoietic stem cells in vitro. Cold Spring Harb. Protoc. *2009*, pdb.prot5156.

Hosogane, M., Funayama, R., Shirota, M., and Nakayama, K. (2016). Lack of transcription triggers H3K27me3 accumulation in the gene body. Cell Rep. *16*, 696–706.

Hosokawa, H., Romero-Wolf, M., Yui, M.A., Ungerbäck, J., Quiloan, M.L.G., Matsumoto, M., Nakayama, K.I., Tanaka, T., and Rothenberg, E.V. (2018). Bcl11b sets pro-T cell fate by site-specific cofactor recruitment and by repressing Id2 and Zbtb16. Nat. Immunol. *19*, 1427–1440.

Howard, J. (2001). Mechanics of Motor Proteins and the Cytoskeleton (Sinauer Associates).

Hu, G., Cui, K., Fang, D., Hirose, S., Wang, X., Wangsa, D., Jin, W., Ried, T., Liu, P., Zhu, J., et al. (2018). Transformation of accessible chromatin and 3D nucleome underlies lineage commitment of early T cells. Immunity *48*, 227–242.e8.

Ikawa, T., Hirose, S., Masuda, K., Kakugawa, K., Satoh, R., Shibano-Satoh, A., Kominami, R., Katsura, Y., and Kawamoto, H. (2010). An essential developmental checkpoint for production of the T cell lineage. Science *329*, 93–96.

Isoda, T., Moore, A.J., He, Z., Chandra, V., Aida, M., Denholtz, M., Piet van Hamburg, J., Fisch, K.M., Chang, A.N., Fahl, S.P., et al. (2017). Non-coding transcription instructs chromatin folding and compartmentalization to dictate enhancer-promoter communication and T cell fate. Cell *171*, 103–119.e18.

Isono, K., Endo, T.A., Ku, M., Yamada, D., Suzuki, R., Sharif, J., Ishikura, T., Toyoda, T., Bernstein, B.E., and Koseki, H. (2013). SAM domain polymerization links subnuclear clustering of PRC1 to gene silencing. Dev. Cell *26*, 565–577.

Jackson, M.B., and Berkowitz, S.A. (1980). Nucleation and the kinetics of microtubule assembly. Proc. Natl. Acad. Sci. USA *77*, 7302–7305.

Jadhav, U., Manieri, E., Nalapareddy, K., Madha, S., Chakrabarti, S., Wucherpfennig, K., Barefoot, M., and Shivdasani, R.A. (2020). Replicational dilution of H3K27me3 in mammalian cells and the role of poised promoters. Mol. Cell *78*, 141–151.e5.

Jiang, D., and Berger, F. (2017). DNA replication-coupled histone modification maintains polycomb gene silencing in plants. Science *357*, 1146–1149.

CellPress
OPEN ACCESS

Johnson, J.L., Georgakilas, G., Petrovic, J., Kurachi, M., Cai, S., Harly, C., Pear, W.S., Bhandoola, A., Wherry, E.J., and Vahedi, G. (2018). Lineage-determining transcription factor TCF-1 initiates the epigenetic identity of T cells. Immunity *48*, 243–257.e10.

Kadoch, C., Williams, R.T., Calarco, J.P., Miller, E.L., Weber, C.M., Braun, S.M.G., Pulice, J.L., Chory, E.J., and Crabtree, G.R. (2017). Dynamics of BAF-polycomb complex opposition on heterochromatin in normal and oncogenic states. Nat. Genet. *49*, 213–222.

Kahn, T.G., Dorafshan, E., Schultheis, D., Zare, A., Stenberg, P., Reim, I., Pirrotta, V., and Schwartz, Y.B. (2016). Interdependence of PRC1 and PRC2 for recruitment to polycomb response elements. Nucleic Acids Res. *44*, 10132–10149.

Kaikkonen, M.U., Spann, N.J., Heinz, S., Romanoski, C.E., Allison, K.A., Stender, J.D., Chun, H.B., Tough, D.F., Prinjha, R.K., Benner, C., and Glass, C.K. (2013). Remodeling of the enhancer landscape during macrophage activation is coupled to enhancer transcription. Mol. Cell *51*, 310–325.

Kent, W.J., Sugnet, C.W., Furey, T.S., Roskin, K.M., Pringle, T.H., Zahler, A.M., and Haussler, D. (2002). The human genome browser at UCSC. Genome Res. *12*, 996–1006.

Kramers, H.A. (1940). Brownian motion in a field of force and the diffusion model of chemical reactions. Physica *7*, 284–304.

Kraushaar, D.C., Jin, W., Maunakea, A., Abraham, B., Ha, M., and Zhao, K. (2013). Genome-wide incorporation dynamics reveal distinct categories of turnover for the histone variant H3.3. Genome Biol. *14*, R121.

Kristensen, J.B.L., Nielsen, A.L., Jørgensen, L., Kristensen, L.H., Helgstrand, C., Juknaite, L., Kristensen, J.L., Kastrup, J.S., Clausen, R.P., Olsen, L., and Gajhede, M. (2011). Enzyme kinetic studies of histone demethylases KDM4C and KDM6A: towards understanding selectivity of inhibitors targeting oncogenic histone demethylases. FEBS Lett. *585*, 1951–1956.

Kueh, H.Y., Yui, M.A., Ng, K.K.H., Pease, S.S., Zhang, J.A., Damle, S.S., Freedman, G., Siu, S., Bernstein, I.D., Elowitz, M.B., and Rothenberg, E.V. (2016). Asynchronous combinatorial action of four regulatory factors activates Bcl11b for T cell commitment. Nat. Immunol. *17*, 956–965.

Kundu, S., Ji, F., Sunwoo, H., Jain, G., Lee, J.T., Sadreyev, R.I., Dekker, J., and Kingston, R.E. (2017). Polycomb repressive complex 1 generates discrete compacted domains that change during differentiation. Mol. Cell *65*, 432–446.e5.

Ladoux, B., Quivy, J.-P., Doyle, P., du Roure, O., Almouzni, G., and Viovy, J.-L. (2000). Fast kinetics of chromatin assembly revealed by single-molecule videomicroscopy and scanning force microscopy. Proc. Natl. Acad. Sci. USA *97*, 14251–14256.

Langmead, B., and Salzberg, S.L. (2012). Fast gapped-read alignment with Bowtie 2. Nat. Methods *9*, 357–359.

Larson, A.G., Elnatan, D., Keenen, M.M., Trnka, M.J., Johnston, J.B., Burlingame, A.L., Agard, D.A., Redding, S., and Narlikar, G.J. (2017). Liquid droplet formation by HP1α suggests a role for phase separation in heterochromatin. Nature *547*, 236–240.

Lee, T.I., Jenner, R.G., Boyer, L.A., Guenther, M.G., Levine, S.S., Kumar, R.M., Chevalier, B., Johnstone, S.E., Cole, M.F., Isono, K., et al. (2006). Control of developmental regulators by polycomb in human embryonic stem cells. Cell *125*, 301–313.

Li, H., Handsaker, B., Wysoker, A., Fennell, T., Ruan, J., Homer, N., Marth, G., Abecasis, G., and Durbin, R.; 1000 Genome Project Data Processing Subgroup (2009). The Sequence Alignment/Map format and SAMtools. Bioinformatics *25*, 2078–2079.

Li, L., Leid, M., and Rothenberg, E.V. (2010). An early T cell lineage commitment checkpoint dependent on the transcription factor Bcl11b. Science *329*, 89–93.

Li, Q., Wang, H.Y., Chepelev, I., Zhu, Q., Wei, G., Zhao, K., and Wang, R.-F. (2014). Stage-dependent and locus-specific role of histone demethylase Jumonji D3 (JMJD3) in the embryonic stages of lung development. PLoS Genet. *10*, e1004524.

Li, H., Natarajan, A., Ezike, J., Barrasa, M.I., Le, Y., Feder, Z.A., Yang, H., Ma, C., Markoulaki, S., and Lodish, H.F. (2019). Rate of progression through a continuum of transit-amplifying progenitor cell states regulates blood cell production. Dev. Cell *49*, 118–129.e7.

MacPherson, Q., Beltran, B., and Spakowitz, A.J. (2018). Bottom-up modeling of chromatin segregation due to epigenetic modifications. Proc. Natl. Acad. Sci. USA *115*, 12739–12744.

Manesso, E., Chickarmane, V., Kueh, H.Y., Rothenberg, E.V., and Peterson, C. (2013). Computational modelling of T-cell formation kinetics: output regulated by initial proliferation-linked deferral of developmental competence. J. R. Soc. Interface *10*, 20120774.

Margueron, R., Justin, N., Ohno, K., Sharpe, M.L., Son, J., Drury, W.J., 3rd, Voigt, P., Martin, S.R., Taylor, W.R., De Marco, V., et al. (2009). Role of the polycomb protein EED in the propagation of repressive histone marks. Nature *461*, 762–767.

Mayran, A., Khetchoumian, K., Hariri, F., Pastinen, T., Gauthier, Y., Balsalobre, A., and Drouin, J. (2018). Pioneer factor Pax7 deploys a stable enhancer repertoire for specification of cell fate. Nat. Genet. *50*, 259–269.

Meers, M.P., Janssens, D.H., and Henikoff, S. (2019). Pioneer factor-nucleosome binding events during differentiation are motif encoded. Mol. Cell *75*, 562–575.e5.

Mitchison, T.J. (1992). Compare and contrast actin filaments and microtubules. Mol. Biol. Cell *3*, 1309–1315.

Newport, J., and Kirschner, M. (1982). A major developmental transition in early Xenopus embryos: I. characterization and timing of cellular changes at the midblastula stage. Cell *30*, 675–686.

Ng, K.K., Yui, M.A., Mehta, A., Siu, S., Irwin, B., Pease, S., Hirose, S., Elowitz, M.B., Rothenberg, E.V., and Kueh, H.Y. (2018). A stochastic epigenetic switch controls the dynamics of T-cell lineage commitment. eLife *7*, e37851.

Nguyen, P.H.B., Pease, N.A., Ng, K.K.H., Irwin, B., and Kueh, H.Y. (2019). Temporal scaling in developmental gene networks by epigenetic timing control (preprint). bioRxiv. https://doi.org/10.1101/752170.

Nuebler, J., Fudenberg, G., Imakaev, M., Abdennur, N., and Mirny, L.A. (2018). Chromatin organization by an interplay of loop extrusion and compartmental segregation. Proc. Natl. Acad. Sci. USA *115*, E6697–E6706.

Okamoto, M., Miyata, T., Konno, D., Ueda, H.R., Kasukawa, T., Hashimoto, M., Matsuzaki, F., and Kawaguchi, A. (2016). Cell-cycle-independent transitions in temporal identity of mammalian neural progenitor cells. Nat. Commun. *7*, 11349.

Osmond, D.G. (1991). Proliferation kinetics and the lifespan of B cells in central and peripheral lymphoid organs. Curr. Opin. Immunol. *3*, 179–185.

Otani, T., Marchetto, M.C., Gage, F.H., Simons, B.D., and Livesey, F.J. (2016). 2D and 3D stem cell models of primate cortical development identify species-specific differences in progenitor behavior contributing to brain size. Cell Stem Cell *18*, 467–480.

Park, D.H., Hong, S.J., Salinas, R.D., Liu, S.J., Sun, S.W., Sgualdino, J., Testa, G., Matzuk, M.M., Iwamori, N., and Lim, D.A. (2014). Activation of neuronal gene expression by the JMJD3 demethylase is required for postnatal and adult brain neurogenesis. Cell Rep. *8*, 1290–1299.

Phillips, R., Kondev, J., Theriot, J., and Garcia, H. (2012). Physical Biology of the Cell (Garland Science).

Plys, A.J., Davis, C.P., Kim, J., Rizki, G., Keenen, M.M., Marr, S.K., and Kingston, R.E. (2018). Phase separation and nucleosome compaction are governed by the same domain of polycomb repressive complex 1. BioRxiv, 467316.

Plys, A.J., Davis, C.P., Kim, J., Rizki, G., Keenen, M.M., Marr, S.K., and Kingston, R.E. (2019). Phase separation of polycomb-repressive complex 1 is governed by a charged disordered region of CBX2. Genes Dev. *33*, 799–813.

Poleshko, A., Mansfield, K.M., Burlingame, C.C., Andrake, M.D., Shah, N.R., and Katz, R.A. (2013). The human protein PRR14 tethers heterochromatin to the nuclear lamina during interphase and mitotic exit. Cell Rep. *5*, 292–301.

Porritt, H.E., Gordon, K., and Petrie, H.T. (2003). Kinetics of steady-state differentiation and mapping of intrathymic-signaling environments by stem cell transplantation in nonirradiated mice. J. Exp. Med. *198*, 957–962.

Quinlan, A.R., and Hall, I.M. (2010). BEDTools: a flexible suite of utilities for comparing genomic features. Bioinformatics *26*, 841–842.

Rinn, J.L., Kertesz, M., Wang, J.K., Squazzo, S.L., Xu, X., Brugmann, S.A., Goodnough, L.H., Helms, J.A., Farnham, P.J., Segal, E., and Chang, H.Y. (2007). Functional demarcation of active and silent chromatin domains in human HOX loci by noncoding RNAs. Cell *129*, 1311–1323.

Sanulli, S., Trnka, M.J., Dharmarajan, V., Tibble, R.W., Pascal, B.D., Burlingame, A.L., Griffin, P.R., Gross, J.D., and Narlikar, G.J. (2019). HP1 reshapes nucleosome core to promote phase separation of heterochromatin. Nature *575*, 390–394.

Schindelin, J., Arganda-Carreras, I., Frise, E., Kaynig, V., Longair, M., Pietzsch, T., Preibisch, S., Rueden, C., Saalfeld, S., Schmid, B., et al. (2012). Fiji: an open-source platform for biological-image analysis. Nat. Methods *9*, 676–682.

Seenundun, S., Rampalli, S., Liu, Q.-C., Aziz, A., Palii, C., Hong, S., Blais, A., Brand, M., Ge, K., and Dilworth, F.J. (2010). UTX mediates demethylation of H3K27me3 at muscle-specific genes during myogenesis. EMBO J. *29*, 1401–1411.

Skene, P.J., Henikoff, J.G., and Henikoff, S. (2018). Targeted in situ genome-wide profiling with high efficiency for low cell numbers. Nat. Protoc. *13*, 1006–1019.

Sneeringer, C.J., Scott, M.P., Kuntz, K.W., Knutson, S.K., Pollock, R.M., Richon, V.M., and Copeland, R.A. (2010). Coordinated activities of wild-type plus mutant EZH2 drive tumor-associated hypertrimethylation of lysine 27 on histone H3 (H3K27) in human B-cell lymphomas. Proc. Natl. Acad. Sci. USA *107*, 20980–20985.

Strom, A.R., Emelyanov, A.V., Mir, M., Fyodorov, D.V., Darzacq, X., and Karpen, G.H. (2017). Phase separation drives heterochromatin domain formation. Nature *547*, 241–245.

Strome, S., Kelly, W.G., Ercan, S., and Lieb, J.D. (2014). Regulation of the X chromosomes in Caenorhabditis elegans. Cold Spring Harb. Perspect. Biol. *6*, a018366.

Sun, B., Looi, L.-S., Guo, S., He, Z., Gan, E.-S., Huang, J., Xu, Y., Wee, W.-Y., and Ito, T. (2014). Timing mechanism dependent on cell division is invoked by Polycomb eviction in plant stem cells. Science *343*, 1248559.

Talbert, P.B., and Henikoff, S. (2017). Histone variants on the move: substrates for chromatin dynamics. Nat. Rev. Mol. Cell Biol. *18*, 115–126.

Tatavosian, R., Kent, S., Brown, K., Yao, T., Duc, H.N., Huynh, T.N., Zhen, C.Y., Ma, B., Wang, H., and Ren, X. (2019). Nuclear condensates of the polycomb protein chromobox 2 (CBX2) assemble through phase separation. J. Biol. Chem. *294*, 1451–1463.

Temple, S., and Raff, M.C. (1986). Clonal analysis of oligodendrocyte development in culture: evidence for a developmental clock that counts cell divisions. Cell *44*, 773–779.

Tu, S., Yuan, G.-C., and Shao, Z. (2017). The PRC2-binding long non-coding RNAs in human and mouse genomes are associated with predictive sequence features. Sci. Rep. *7*, 41669.

Ulianov, S.V., Doronin, S.A., Khrameeva, E.E., Kos, P.I., Luzhin, A.V., Starikov, S.S., Galitsyna, A.A., Nenasheva, V.V., Ilyin, A.A., Flyamer, I.M., et al. (2019). Nuclear lamina integrity is required for proper spatial organization of chromatin in Drosophila. Nat. Commun. *10*, 1176.

van Steensel, B., and Belmont, A.S. (2017). Lamina-associated domains: links with chromosome architecture, heterochromatin, and gene repression. Cell *169*, 780–791.

Varnum-Finney, B., Wu, L., Yu, M., Brashem-Stein, C., Staats, S., Flowers, D., Griffin, J.D., and Bernstein, I.D. (2000). Immobilization of Notch ligand, Delta-1, is required for induction of notch signaling. J. Cell Sci. *113*, 4313–4318.

Walters, M.C., Fiering, S., Eidemiller, J., Magis, W., Groudine, M., and Martin, D.I. (1995). Enhancers increase the probability but not the level of gene expression. Proc. Natl. Acad. Sci. USA *92*, 7125–7129.

Wang, L., Gao, Y., Zheng, X., Liu, C., Dong, S., Li, R., Zhang, G., Wei, Y., Qu, H., Li, Y., et al. (2019). Histone modifications regulate chromatin compartmentalization by contributing to a phase separation mechanism. Mol. Cell *76*, 646–659.e6.

Weintraub, H. (1988). Formation of stable transcription complexes as assayed by analysis of individual templates. Proc. Natl. Acad. Sci. USA *85*, 5819–5823.

Williams, K., Christensen, J., Rappsilber, J., Nielsen, A.L., Johansen, J.V., and Helin, K. (2014). The histone lysine demethylase JMJD3/KDM6B is recruited to p53 bound promoters and enhancer elements in a p53 dependent manner. PLoS ONE *9*, e96545.

Woodworth, M.A., Ng, K.K.H., Halpern, A.R., Pease, N.A., Nguyen, P.H.B., Kueh, H.Y., and Vaughan, J.C. (2020). Multiplexed single-cell profiling of chromatin states at genomic loci by expansion microscopy (preprint). bioRxiv. https://doi.org/10.1101/2020.11.17.385476.

Xu, J., Ma, H., Jin, J., Uttam, S., Fu, R., Huang, Y., and Liu, Y. (2018). Super-resolution imaging of higher-order chromatin structures at different epigenomic states in single mammalian cells. Cell Rep. *24*, 873–882.

Zaret, K.S., and Carroll, J.S. (2011). Pioneer transcription factors: establishing competence for gene expression. Genes Dev. *25*, 2227–2241.

Zhang, J.A., Mortazavi, A., Williams, B.A., Wold, B.J., and Rothenberg, E.V. (2012). Dynamic transformations of genome-wide epigenetic marking and transcriptional control establish T cell identity. Cell *149*, 467–482.

Zhang, H., Tian, X.-J., Mukhopadhyay, A., Kim, K.S., and Xing, J. (2014). Statistical mechanics model for the dynamics of collective epigenetic histone modification. Phys. Rev. Lett. *112*, 068101.

Zheng, M., Tian, S.Z., Capurso, D., Kim, M., Maurya, R., Lee, B., Piecuch, E., Gong, L., Zhu, J.J., Li, Z., et al. (2019). Multiplex chromatin interactions with single-molecule precision. Nature *566*, 558–562.

Zhou, C.Y., Johnson, S.L., Gamarra, N.I., and Narlikar, G.J. (2016). Mechanisms of ATP-dependent chromatin remodeling motors. Annu. Rev. Biophys. *45*, 153–181.

Zhou, W., Yui, M.A., Williams, B.A., Yun, J., Wold, B.J., Cai, L., and Rothenberg, E.V. (2019). Single-cell analysis reveals regulatory gene expression dynamics leading to lineage commitment in early T cell development. Cell Syst. *9*, 321–337.e9.

STAR★METHODS

KEY RESOURCES TABLE

REAGENT or RESOURCE	SOURCE	IDENTIFIER
Antibodies		
Anti-mouse Ter119 Biotin (clone TER-119)	eBioscience	Cat#13-5921-85; RRID:AB_466798
Anti-mouse NK1.1 Biotin (clone PK136)	eBioscience	Cat#13-5941-85; RRID:AB_466805
Anti-mouse Gr-1 Biotin (clone RB6-8C5)	eBioscience	Cat#13-5931-86; RRID:AB_466802
Anti-mouse CD11c Biotin (clone N418)	eBioscience	Cat#13-0114-85; RRID:AB_466364
Anti-mouse CD11b Biotin (clone M1/70)	eBioscience	Cat#13-0112-86; RRID:AB_466361
Anti-mouse CD19 Biotin (clone 1D3/6D5)	eBioscience	Cat#13-0193-85; RRID:AB_657658
Anti-mouse CD3e Biotin (clone 145–2 C11)	eBioscience	Cat#13-0031-85; RRID:AB_466320
Anti-human/mouse B220 Biotin (clone RA3-6B2)	eBioscience	Cat#13-0452-85; RRID:AB_466450
Anti-human/mouse CD44 eFluor 450 (clone IM7)	eBioscience	Cat#48-0441-82; RRID:AB_1272246
Anti-mouse CD25 APC-eFluor 780 (clone PC61.5)	eBioscience	Cat#47-0251-82; RRID:AB_1272179
Streptavidin PerCP-Cyanine5.5	Biolegend	Cat#405214; RRID:AB_2716577
APC Annexin V	Biolegend	Cat#640919
Bacterial and virus strains		
Stable Competent *E.coli*	NEB	Cat#C3040
Chemicals, peptides, and recombinant proteins		
Recombinant Human Flt3-Ligand	PreproTech	Cat#300–19
Recombinant Human IL-7	PreproTech	Cat#200–07
Recombinant Human Stem Cell Factor (SCF)	PreproTech	Cat#300–07
Anti-H3K27me3 antibody	Active Motif	Cat#39156
Retronectin	Takara	Cat#T100B
DL1-ext IgG Protein	Gift from Irwin Bernstein (Varnum-Finney et al., 2000)	N/A
FuGENE 6 Transfection Reagent	Promega	Cat#E2691
UNC1999	Caymen Chemical	Cat#14621
GSKJ4	Caymen Chemical	Cat#12073
IOX1	Caymen Chemical	Cat#11572
GSK126	Caymen Chemical	Cat#15415
GSK343	Caymen Chemical	Cat#14094
Concavalin A	Bangs Laboraatories	Cat#BP531
protein A-MNase	gift from Steven Henikoff (Skene et al., 2018)	N/A
Deposited data		
T cell progenitor H3K27me3 CUT&RUN	This paper	Gene Expression Omnibus GSE134749
Thymocyte H3K27ac ChIP-sequencing	Davis et al., 2018	ENCODE accession ENCSR000CCH
Erythroblast H3K9me3 ChIP-sequencing	Davis et al., 2018	ENCODE accession ENCSR000DHN
T cell progenitor H3K27me3 ChIP-sequencing	Zhang et al., 2012	Gene Expression Omnibus GSE31235
DN3-DP thymocyte Hi-C	Hu et al., 2018	Gene Expression Omnibus GSE79875
Experimental models: cell lines		
OP9-DL1-GFP	Holmes and Zúñiga-Pflücker, 2009	N/A
Kasumi-1	ATCC	Cat#CRL-2724; RRID:CVCL_0589
Pheonix-Eco	ATCC	Cat#CRL-3214, RRID:CVCL_H717

(*Continued on next page*)

Continued

REAGENT or RESOURCE	SOURCE	IDENTIFIER
Experimental models: organisms/strains		
Mouse: Bcl11b$^{RFP/YFP}$	Ng et al., 2018	N/A
Oligonucleotides		
Bcl11b forward primer: TCCACCTACCAGACCCCGAA	IDT	N/A
Bcl11b reverse primer: CTTCTTCAAAGTGCTTGGCCTC	IDT	N/A
PAX5 forward primer: CCAGGATGTGCTGCTGTCCCAG	IDT	N/A
PAX5 reverse primer: CTCCCTGGTGCTGTGCACTGA	IDT	N/A
mir-30-shEed template: TGCTGTTGACAGTGAGCGAAGGCATTATAA GAATAATTAATAGTGAAGC CACAGATGTAT TAATTATTCTTATAATGCCTCTGCCTACTGC CTCGGA	IDT	N/A
Recombinant DNA		
pBAD-mTagBFP2	Addgene	Cat #34632
pMSCV-miR-30	Gift from Johannes Zuber (Fellmann et al., 2013)	N/A
pMSCV-c-Myc-H2B-mCerulean	Kueh et al., 2016	N/A
pBanshee-CFP	Kueh et al., 2016	N/A
pCL-Eco	Imgenex	Cat#NBP2-29540
Software and algorithms		
Software: FlowJo (v10.0.8)	Tree Star	https://www.flowjo.com/
Software: MATLAB (R2018b)	MathWorks	https://www.mathworks.com/products/matlab.html
Software: R (v3.9)	N/A	https://www.r-project.org/
Rstudio (v1.2.5042)	N/A	https://rstudio.com/
Ggplot2 (v.3.3)	N/A	https://ggplot2.tidyverse.org/
Bedtools (v2.17.0)	Quinlan and Hall, 2010	https://bedtools.readthedocs.io/en/latest/
Samtools (v0.1.19-96b5f2294a)	Li et al., 2009	http://samtools.sourceforge.net/
Fiji (v2.0.0)	Schindelin et al., 2012	https://imagej.net/Downloads
BUnwarpJ	Arganda-Carreras et al., 2006	https://imagej.net/BUnwarpJ
Python(v3.6)	N/A	https://www.python.org
Other		
BD FACS Aria III Cell Sorter	BD Biosciences	N/A
NucleoSpin PCR Clean-up	Macherey-Nagel	Cat#740609.50
Illumina MiSeq	Illumina	N/A
Attune NxT Acoustic Focusing Cytometer	ThermoFisher Scientific	N/A
KAPA HyperPrep Kit	Roche	Cat#07962312001
PowerUp SYBR Green Master Mix	ThermoFisher Scientific	Cat#A25741
Ampure XP magnetic beads	Beckman Coulter	Cat#A63880
CFX96 Real-Time PCR Detection System	Bio-Rad	Cat#1855196
CD117 Microbeads	Miltenyi Biotec	Cat#130-091-224
LS Columns	Miltenyi Biotec	Cat#130-042-401
250mm-diameter PDMS circular micromesh arrays	Microsurfaces Pty Ltd	Cat#MMA-0250-100-08-01
Custom scripts for imaging analysis and simulations	This paper	https://github.com/KuehLabUW/Pease_et_al.2021

RESOURCE AVAILABILITY

Lead contact

Further information and requests for resources and reagents should be directed to and will be fulfilled by the Lead Contact, Hao Yuan Kueh (kueh@uw.edu).

Materials availability

The pMSCV-mTagBFP2-shEed plasmid is available from the Lead Contact upon request.

Data and code availability

The accession number for the H3K27me3 CUT&RUN data reported in this paper is NCBI Gene Expression Omnibus: GSE134749. Scripts used for imaging analysis and simulations of mathematical models have been deposited to GitHub: https://github.com/KuehLabUW/Pease_et_al.2021. All other data supporting the findings of this paper will be available from the Lead Contact.

EXPERIMENTAL MODEL AND SUBJECT DETAILS

Animal Models

C57BL/6 *Bcl11b*$^{RFP/YFP}$ mice were generated as described before (Ng et al., 2018). Briefly, *Bcl11b*$^{YFP/YFP}$ mice were generated by inserting an IRES-H2B-mCitrine-neo cassette into the 3′ UTR of *Bcl11b* and *Bcl11b*$^{RFP/RFP}$ mice were generated by inserting an IRES-H2B-mCherrry-neo cassette into the same location. Dual allelic *Bcl11b*$^{RFP/YFP}$ mice with identical *Bcl11b* alleles except for fluorescent protein reporters were generated by breeding *Bcl11b*$^{RFP/RFP}$ mice to *Bcl11b*$^{YFP/YFP}$ mice. Bone marrow derived from F1 *Bcl11b*$^{RFP/YFP}$ mice at 2-4 months of age were used for all *in vitro* T cell development assays. Sex was determined not to be influential for these studies, thus male and female bone marrow were combined and analyzed together. All animals were bred and maintained at the University of Washington. All animal protocols were reviewed and approved by the Institute Animal Care and Use Committee at the University of Washington (Protocol No: 4397-01).

Cell Line Culture

Primary cells isolated from bone marrow were cultured on a OP9-DL1 monolayer stromal cells (Holmes and Zuniga-Pflucker, 2009) at 37°C in 5% CO_2 conditions with standard culture medium [80% aMEM (GIBCO), 20% Fetal Bovine Serum (Corning), Pen-Strep-Glutamine (GIBCO)] supplemented with appropriate cytokines (described in Method Details). Phoenix-Eco cells were cultured at 37°C in 5% CO_2 with standard culture medium [90% DMEM (GIBCO), 10% Fetal Bovine Serum (Corning), Pen-Strep-Glutamine (GIBCO)] All cell lines were tested and found to be negative for mycoplasma contamination.

METHOD DETAILS

Cell purification

To isolate hematopoietic stem and progenitor cells (HSPCs) for *in vitro* differentiation or CUT&RUN experiments, bone marrow cells were harvested from femurs and tibias of 2 to 4 month-old *Bcl11b*$^{RFP/YFP}$ mice. CD117 MicroBeads (Miltenyi Biotec) were used to enrich HPSCs which were frozen in 90% FBS and 10% DMSO at 10^6 cells/mL. For CUT&RUN experiments, HPSCs were further purified by staining with anti-CD117 APC-eFluor780 (ThermoFisher Scientific) and with biotinylated antibodies against a panel of bone marrow lineage markers (CD19, CD11b, CD11c, NK.1.1, Ter119, CD3ε, Gr-1 and B220 (BioLegend)). Cells were then washed with HBH (Hank Balanced Salt Solution (HBSS) with 0.1% bovine serum albumin and 10mM HEPES) and stained with streptavidin-PerCP/Cy5.5 (BioLegend).

In vitro differentiation of T cell progenitors

To generate double-negative (DN) T cells *in vitro*, thawed CD117-enriched bone marrow progenitors were cultured on OP9-DL1 stromal cell monolayers as described before using standard culture medium [80% αMEM (GIBCO), 20% Fetal Bovine Serum (Corning), Pen-Strep-Glutamine (GIBCO)], grown at 37°C in 5% CO2 conditions]. All *in vitro* T cell generation cultures were supplemented with 5ng/mL Flt3-L and 5 ng/mL IL-7 (Peprotech), and were sorted after 6 to 8 days of culture before transducing with retroviral vectors or treating with small molecule inhibitors. DN2 cells were re-cultured in the same conditions following all cell sorting experiments.

Flow cytometry and cell sorting

Fluorescence activated cell sorting (FACS) was used to isolate DN2 cells of interest with the following protocol. Bone marrow derived cell cultures were scraped and incubated in 2.4G2 Fc blocking solution and stained with anti-CD25 APC-eFluor 780 (Clone PC61.5, eBioscience) and with biotinylated antibodies against a panel of lineage markers (CD19, CD11b, CD11c, NK.1.1, Ter119, CD3ε, Gr-1 and B220 (BioLegend)). Stained cells were washed with HBH (Hank Balanced Salt Solution (HBSS) with 0.1% bovine serum albumin (BSA) and 10mM HEPES and stained with streptavidin-PerCP/Cy5.5 (BioLegend). Stained cells were washed, resuspended in HBH, and filtered through a 40-um nylon mesh for sorting with a BD FACS Aria III (BD Biosciences) with assistance from the University of

Washington Pathology Flow Cytometry Core Facility. A benchtop MacsQuant VYB flow cytometer (Miltenyi Biotec) and a benchtop Attune NxT Flow Cytometer (ThermoFisher Scientific) were used to analyze time course and perturbation experiments and acquired data were analyzed with FlowJo software (Tree Star).

Retroviral construct and transduction

Overexpression of c-Myc was achieved using cMyc H2B-mCerulean MSCV retroviral vector which was described previously (Kueh et al., 2016). Retroviral mir30-based constructs (a gift from J. Zuber) were used as a backbone for delivering short hairpin RNA (Fellmann et al., 2013). pBAD-mTagBFP2 (a gift from V. Verkhusha, Addgene plasmid #34632) was used to substitute mTagBFP2 for the existing GFP using PCR cloning with the restriction enzymes NcoI and SalI. The pMSCV-mTagBFP2-shEed retroviral construct was generated by PCR cloning as previously described (Fellmann et al., 2013) using the mir-30-shEed PCR template sequence and pMSCV-mir-30 backbone described in the Key resources table.

Retroviral particles were generated using the Phoenix-Eco packaging cell line. Viral supernatants were collected at 2 and 3 days after transfection and immediately frozen at -80°C. To infect bone marrow derived T cell progenitors, 33 μg/mL retronectin (Clontech) and 2.67 μg/mL of DL1-extracellular domain fused to human IgG1 Fc protein (a gift from I. Bernstein) were added in a volume of 250 μL per well in 24-well tissue culture plates (Costar, Corning) and incubated overnight. Viral supernatants were added the next day into coated wells and centrifuged at 2000 *rcf.* for 2 hours at 32°C. Bone marrow derived derived T cell progenitors used for viral transduction were cultured for 6-7 days according to conditions described above, disaggregated, filtered through a 40-μm nylon mesh, and 10^6 cells were transferred onto each retronectin/DL1-coated virus-bound well supplemented with 5 ng/mL SCF (Peprotech), 5 ng/mL Flt3-L, and 5 ng/mL IL-7.

CUT&RUN H3K27me3 profiling

CUT&RUN experiments were carried out as previously described (Skene et al., 2018) with the following modifications: 1-2.5x10^5 cells were isolated by FACS as described in sections above, bound to Concanavalin A coated magnetic beads (Bangs Laboratories), and permeabilized with 0.025% (wt/vol) digitonin. Permeabilized cells were incubated overnight at 4°C with 5ug of anti-H3K27me3 (Active Motif) and then washed before incubating with protein A-MNase fusion protein (a gift from S. Henikoff) for 15 minutes at room temperature. After washing, cells were incubated in $CaCl_2$ to induce MNase cleavage activity for 30 minutes at 0°C. The reaction was stopped with 2xSTOP buffer (200 mM NaCl, 20 mM EDTA, 4 mM EGTA, 50 μg/mL RNase A, 50 μg/mL glycogen, and 2pg/mL of yeast spike-in DNA). Histone-DNA complexes were isolated from insoluble nuclear chromatin by centrifugation and DNA was extracted with a NucleoSpin PCR Clean-up kit (Macherey-Nagel). For CUT&RUN quantitative PCR, human Kasumi-1 cell line (ATCC CRL-2724) were added before binding the cells to Concanavalin A beads for internal standard instead of yeast spike-in DNA.

CUT&RUN library preparation and sequencing

Library preparation from CUT&RUN products was completed with KAPA Hyper Prep Kit (KAPA Biosystems) following standard protocol with PCR amplification settings adjusted so that annealing and extension steps are combined into one step at 60°C for 10 s. Library products were size selected to be within 200 - 300 bp range using AMPure beads (Agencourt). Libraries were sequenced using an Illumina MiSeq system with paired-end 25 bp sequencing read length and TruSeq primer standard for approximately 5 millions reads per sample.

CUT&RUN sequencing analysis

Paired-end sequencing reads were aligned separately to mouse (NCBI37/mm9) and yeast (SacCer_Apr2011/sacCer3) genomes using Bowtie2 (Langmead and Salzberg, 2012) with the following setting:–local–very-sensitive-local–no-unal–no-mixed–no-discordant -I 10 -X 700 as suggested for mapping CUT&RUN sequencing data (Skene et al., 2018). The alignment setting was designed to specifically search with high stringency for only appropriately paired reads with the proper orientation. The resulting alignments were converted to BAM files with SAMtools (Li et al., 2009) and then converted to BED files with BEDTools (Quinlan and Hall, 2010). Reads were sorted and filtered to remove random chromosomes. BEDTools genomecov was used to generate histograms for the mapped reads using a scaling factor that is the product of the number of spiked-in yeast reads and the number of input cells. The resulting bedGraph files were visualized using the UCSC Genome Browser (Davis et al., 2018; Kent et al., 2002).

CUT&RUN qPCR

Extracted DNA from CUT&RUN samples was size selected with Ampure XP magnetic beads (Beckman Coulter) to remove fragments > 800bp. Primers were designed to detect the mouse *Bcl11b* promoter (see Key resources table for sequences). PowerUp SYBR Green Master Mix (ThermoFisher Scientific) and CFX96 Real-Time PCR Detection System (Bio-Rad) were used for quantitative PCR. Since Kasumi-1 cells were used as internal standard, relative enrichment of H3K27me3 at *Bcl11b* was quantified by the ΔΔCq method using the human *PAX5* promoter for normalization to account for differences in efficiency and sample loss during processing.

Cell preparation for time-lapse imaging

T cell progenitors derived from the *in vitro* differentiation protocol above were harvested and infected with either a MSCV empty vector or c-Myc overexpression vector harboring an IRES-H2B-mCerulean reporter cassette. 16-24 hours later CFP-positive cells were

purified by FACS and seeded onto PDMS micromesh (250 μm hole diameter, Microsurfaces) mounted on top of a 24-well glass bottom plate (Mattek). To prepare the stromal-free differentiation system, which facilitates cell identification during imaging, the top face of PDMS micromesh was first blocked by incubating in solution of 130 μg/ml BSA while mounted on top of a 24-well plate overnight at 4°C. This step prevents subsequent binding of retronectin to the side of the micromesh walls. Blocked micromeshes was then transferred to a clean 24-well glass bottom plate. The well and mesh constructs were incubated in a solution of 10 μg/ml retronection and 3 μg/ml DL-1 overnight at 4°C. The well was then washed with PBS, and culture media [80% αMEM (GIBCO), 20% Fetal Bovine Serum (Corning), Pen-Strep-Glutamine (GIBCO), 5 ng/ml IL-7 (Clontech), 5 ng/ml Flt-3 (Clontech), 50 ng/ml mSCF (Clontech), 50 μM beta-mercaptoethanol (Sigma) grown at 37°C in 5% CO2 conditions] was added, and sorted cells were introduced at a concentration of 5-10 cells per microwell. This stromal-free system enables a greater fold enhancement of cell division rate by cMyc transduction and better resolution for imaging as well as recapitulating Bcl11b activation and T cell lineage commitment, but supports a lower baseline rate of proliferation in unmodified cells compared to the OP9-DL1 system.

Oligopaint DNA-FISH

The OligoMiner pipeline was used to design Oligopaint libraries (Beliveau et al., 2018). 35-52bp probes were designed to target 20kb regions at a density of approximately 12-14 probes per kilobase. Bone marrow progenitors were grown on OP9-DL1 stromal cells for 8 days under normal growth conditions. Cells were filtered through a 70uM filter and incubated with 2.4G2 blocking buffer before staining with anti-CD25 APC-eFluor 780 (Clone PC61.5, eBioscience), anti-CD44 APC (Clone IM7, eBioscience), and biotinylated antibodies against a panel of lineage markers (CD19, CD11b, CD11c, NK.1.1, Ter119, CD3ε, Gr-1 and B220 (BioLegend)). DN1 progenitors ($CD25^-/CD44^+/Bcl11b^{RFP-/YFP-}$), DN2a progenitors ($CD25^+/CD44^+/Bcl11b^{RFP-/YFP-}$) and DN2b progenitors ($CD25^+/CD44^+/Bcl11b^{RFP+/YFP+}$) were purified by FACS and centrifuged on top of poly-L-lysine coated 18-well chambered glass coverslips (Ibidi). Cells were then fixed with 4% paraformaldehyde for 10 minutes and permeabilized for 10 minutes in 0.1% Triton X-100 before performing the Oligopaint DNA-FISH protocol (Beliveau et al., 2017). Permeabilized cells were incubated in 0.1N HCl for 5 minutes, followed by RNaseA (100ug/ml) for 1 hour at 37°C. Cells were then washed with 2x SSCT (2x saline sodium citrate + 0.1% Tween-20) and incubated with 2x SSCT + 50% formamide for 20 minutes at 60°C. A hybridization mixture was prepared containing 50% formamide, 2x SSCT, 3mM sodium azide, 10% dextran sulfate, 100nM of adaptor oligos, 100nM of fluorescently labeled reporter oligos, and 500nM of probes. The hybridization mixture was added and incubated for 3 minutes at 78°C before incubating overnight in a humidifier chamber at 37°C. Approximately 18 hours later, cells were washed with pre-heated 2x SSCT for 5 minutes at 60°C. This step was repeated four times before performing the final wash at room temperature. Cells were then stained with 10ug/mL Hoechst 33342 (ThermoFisher) for 15 minutes before washing with PBS and imaging in with a photoprotective buffer (10% glucose, 200mM Tris, glucose oxidase (GLOX), catalase, 1mM methyl viologen hydrate, 1mM ascorbic acid).

FISH imaging and analysis

Cells were imaged with an inverted widefield fluorescence microscope (Leica DMi8) using a 100X oil objective, using an sCMOS camera (Photometrics Prime 95B) and a motorized stage (ASI MS-2000). Z sections were collected at a step size of 100nm. Chromatic aberrations were corrected for using Fiji (Schindelin et al., 2012) and BUnwarpJ (Arganda-Carreras et al., 2006) as described previously (Giorgetti et al., 2015). After nuclei segmentation, the *z* slice with the maximum intensity for each foci was chosen as the *z* coordinate. Each selected *z* slice was then fit to a two-dimensional Gaussian to determine the *xy* coordinates for the centroid of the foci. Euclidean distances between each pair of foci and between each labeled promoter focus and the nearest nuclear edge were calculated.

QUANTIFICATION AND STATISTICAL ANALYSIS

The following statistical tests were used in this study: two-sample, one-tailed t test (Figures 2E, 2F, 3B, 3C, and S1D); and the Mann Whitney U-test (Figures 5C and 5D). Details for statistical tests performed are described in the indicated figure legends. All statistical tests were performed using MATLAB or R.

Modeling simulations

All models were simulated using the Gillespie algorithm provided in the Tellurium package in Python 2.7 (Choi et al., 2018). Plotting of simulation results was done in MATLAB. A detailed description of the models can be found in the mathematical appendix (see Methods S1: Mathematical Appendix, related to STAR Methods).

Image analysis of time-lapse movies

Image segmentation

Cell segmentation was performed in MATLAB (Mathworks, Natick, MA) using custom scripts described previously (Kueh et al., 2016; Ng et al., 2018). The segmentation algorithm was performed on CFP fluorescent signals as all transduced cells carried an H2B-mCerulean CFP reporter cassette. Briefly, images underwent (1) correction by subtraction of uneven background signal stemming from the bottom of the glass plate or the side of the PDMS microwells (2) Gaussian blur followed by pixel value saturation to fix uneven signal intensity within the nucleus of the cell and (3) Laplacian edge detection algorithm to identify the nucleus boundary. Non-cell

objects were excluded via size and shape limit exclusions, and segmentation parameters were chosen such that the number of non-cell objects are < 1% of the total segmented cells.

Identification of live and dead cell population

In movies of cMyc or empty vector (EV) transduced cells, live and dead cells possessed distinct morphological features as observed in the CFP fluorescence channel. Live cell nuclei had a round, smooth oval shape while dead cell nuclei tended to be more granular, with small but very bright puncta. To provide unbiased, automated recognition of live and dead cells based on these features, we applied a Laplacian mask filter to each segmented cell to delineate the 'smoothness' of its signal, then applied a threshold-cutoff to identify regions with high CFP signal. The resultant list of object features were recorded for each cell object: 1) nuclei area, 2) perimeter, 3) fluorescent intensity, 4) puncta number, 5) mean puncta area, 6) mean puncta perimeter, and 7) area for puncta above the cut-off threshold. Approximately one hundred individual cell images (10% of each dataset) were then manually annotated as 'live' or 'dead'. Annotations were then linked to the above feature matrix, and a decision tree supervised machine learning algorithm was then used to generate a model based on the annotated live/dead classification and matrix features of the training images (Figures S2A–S2C). Finally, built-in MATLAB model evaluation functions resubLoss and crossval were used to validate that mis-assignment error is below 15% for all datasets. This approach was utilized to provide an objective, automated method to distinguish between live and dead populations.

Bcl11b activation rate fitting

The following procedure was used to quantify *Bcl11b* activation rate from timelapse movies: first, the YFP and RFP signal intensity of segmented cells were calculated. Next, each cell object was classified as 'live' or 'dead', using classification prediction by trained model described in the previous section. Cells classified as 'live' were selected, and their YFP RFP fluorescence 2D histograms were then fitted to a two-component mixed 2D Gaussian model to obtain the fraction of YFP-OFF and YFP-ON cells in the population at a given time. To calculate background-corrected fluorescent values of the Bcl11b YFP and Bcl11b RFP signals, we calculated the pixel intensity of an annulus surrounding the segmented cell and subtracted this value from the raw signal intensity in the cell interior. This approach eliminates autofluorescence from the bottom of the glass plate as well as at the edge of the PDMS microwell.

To obtain the time evolution of Bcl11b biallelic population fractions from initial Bcl11b YFP-RFP+ population, cells were first filtered based on their 'live/dead' category, and only 'live' cells were included in subsequent calculations. We used a modified version of least-squares fit of a two-component mixed 2D Gaussian function described by Ng et al. (2018) to fit the 2D histogram of Bcl11b YFP and Bcl11b RFP fluorescence levels. Specifically, let y and r be the intensity of Bcl11b YFP and Bcl11b RFP fluorescence, respectively, the overall fit, $F(r,y)$, is given by:

$$F(r,y) = \sum_{i=1}^{2} f_i(r,y) \qquad \text{(Equation 1)}$$

Each 2D Gaussian f is given by:

$$f_i(r,y) = \frac{N_i}{2\pi\sigma_{r,i}\sigma_{y,i}\sqrt{1-\rho_i^2}} \cdot exp\left(-\frac{1}{2(1-\rho_i^2 r^2)}\left[\frac{(r-\mu_{r,i})^2}{\sigma_{r,i}^2} + \frac{(y-\mu_{y,i})^2}{\sigma_{r,i}^2} + \frac{2\rho(r-\mu_{r,i})(y-\mu_{r,i})}{\sigma_{r,i}\sigma_{y,i}}\right]\right) \qquad \text{(Equation 2)}$$

Here, i = 1,2 correspond to the red mono-allelic and biallelic populations, since all starting cells are red mono-allelic, we excluded the other two populations (non-expressing and yellow mono-allelic). N_i is the volume under the Gaussian curve when integrated over r and y and is the approximation for the number of cells in each population in Bcl11b RFP mono-allelic and biallelic states.

To fit our data to $F(r,y)$, we followed a two-step process described previously (Ng et al., 2018): (1) We fitted Bcl11b YFP/RFP 2D histogram at an early time point (0 < t < 20) to $f_1(r,y)$to obtain the means, standard deviations, and correlation coefficients $(u_{r,1}, \sigma_{r,1}, u_{y,1}, \sigma_{y,1}, p_1)$) of the Bcl11b RFP mono-allelic population. At this early time point, cells remained inactive for the Bcl11b YFP allele. (2) Next, we fitted the 2D histograms of Bcl11b YFP/RFP levels at successive time bins of 20 hours, fixing the parameter of the first Gaussian $f_1(r,y)$, and enabling the parameters for the second Gaussian $f_2(r,y)$, to vary within bounds observed in the fluorescent distributions of Bcl11b biallelic populations. After fitting, the fraction of biallelic cell at a given time window centered on time t is given by:

$$f_2^{obs}(t) = \frac{N_2(t)}{N_1(t)+N_2(t)} \qquad \text{(Equation 3)}$$

The confident bounds for $f_i^{obs}(t)$is given by:

$$\delta f_i^{obs}(t) = f_i^{obs}\sqrt{\left(\frac{\delta N_i}{N_i}\right)^2 + \frac{\sum_{i=1}^{2}\delta N_i^2}{\left(\sum_{j=1}^{2}N_j\right)^2}} \qquad \text{(Equation 4)}$$

Afterward, the resulting fraction of biallelic cells as a function of time window centered at time t from the mixed Gaussian fit was then fitted to the probability density function of a first order process:

$$F_{bi}(t) = 1 - e^{-\lambda t} \qquad \text{(Equation 5)}$$

Where λ is the rate for activation of the initially silent Bcl11b-YFP allele. We chose this function for activation rate fitting since our histone dynamics simulations suggested that Bcl11b activation can be estimated as a first order stochastic process (see Figure 4D). For this function, fitting was done using the MATLAB fit function and a 95% confidence interval for the fit was recorded.

Population dynamics model and fitting

We built a mathematical model to describe the population dynamics of progenitor cells transfected with an empty vector (EV) and c-Myc. From initial inspection of time-lapse movies (Figure 3), progenitors transduced with c-Myc appear to expand more quickly than control progenitors, as expected. Faster expansion of c-Myc-transduced cells could be due to faster cell cycling or slower cell death. To disentangle these two effects, we quantified numbers of both live and dead cells over time (Figure 3B) and fit these data to population dynamics models to obtain division and death rates:

The model includes a population of live cells (X) with a division rate k_b and a death rate k_d to generate the observable dead cell population (Y). This population in turn has a clearance rate δ representing the process by which CFP level degrades and dead cells become undetectable.

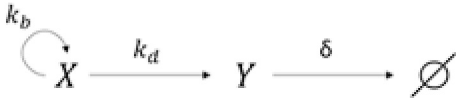

We then obtained the division rate k_b and the death rate k_d through a two-step curve fitting process. First the difference between these two rates$K = k_b - k_d$was obtained by fitting the number of live cells over time::

$$X(T) = X_o e^{(k_b - k_d)T} = X_o e^{KT} \quad \text{(Equation 6)}$$

where X_o is the initial number of live cells at the start of imaging. The death rate was then obtained by fitting the number of observable dead cells over time, which, given the above transition scheme, is given by the following: :

$$Y(T) = Y_o P(T) + \sum_{t=1}^{T} X(t) \cdot k_d \cdot P(T-t) \quad \text{(Equation 7)}$$

Here $Y_o$is initial observed number of dead cells at the start of imaging and $P(\tau)$gives the probability of the dead cell remaining observable in the CFP fluorescent channel a period of time τ after its first appearance. In this model, whenever a cell starts to die, its probability of being detected decreases as per function $P(\tau)$, and the number of dead cells at a given time T is the sum of all the still-detectable dead cells generated since the start of imaging up until T. This decrease in detection probability arises because progressive dimming of CFP fluorescence, together with morphological changes following death results in the failure of the cells to be segmented.

We determined $P(\tau)$ empirically for EV and cMyc population separately by manually following 30 different dead cells and recording the time period in which it was detected and undetected until complete disappearance. We then calculated the fraction of dead cells that remained detectable after a given time had elapsed. An exponential function decay function was used to fit this 'fraction detected' curve and to estimate value for clearance rate (Figures S2B and S2C):

$$P(\tau) = e^{-\delta\tau} \quad \text{(Equation 8)}$$

Here, $P(\tau)$ is the probability of a given dead cell to be detected under the CFP fluorescent channel after a period of time since its initial death. δ is the clearance rate of this process.

To fit imaging data to Equation 6, we classified segmented cell objects as live or dead using a trained machine learning model as described in 'Image analysis of time-lapse movies' section. Number of live cells as a function of time was fitted to Equation 6 using a MATLAB fit function and 95% confidence interval for the fit was recorded.

To fit imaging data to Equation 7, we tested a series of candidate $k_{d,i}$values; for each $k_{d,i}$, a predicted $Y_{p,i}(t)$curve was generated based on Equation 7 where $T = t_1, t_2, t_3, \ldots$with t_i being the time point at which experimental measurement took place. $Y_{p,i}(T)$is then compared to the experimentally observed dead cell number $Y_{exp}(T)$using sum square error method:

$$sse_i = \sum_{t = t_1, t_2, t_3, \ldots} \left[Y_{exp}(t) - Y_{p,i}(t)\right]^2 \quad \text{(Equation 9)}$$

The best fit $k_d$value is chosen to be the value $k_{d,i}$, whose $sse_i$is the smallest.

In order to calculate the confidence bound of the fit, we first performed a nonlinear regression by calculating the residuals of the model's predicted values $Y_p(t_i)$:

$$dY_{t_i} = Y_{exp}(t_i) - Y_p(t_i) \quad \text{(Equation 10)}$$

We then calculated to the Jacobian of the model function to estimate the covariance at each time point, given by:

$$J_{t_i} = \frac{\partial Y_p(t_i)}{\partial k_d} \quad \text{(Equation 11)}$$

These inputs were used to estimate 95% confidence interval using MATLAB ‘Nonlinear regression parameter confidence intervals’ function nlparci.

A summary of results from data fitting are tabulated in Table S1.

Cell Reports, Volume *34*

Supplemental information

Tunable, division-independent control

# of gene activation timing by a polycomb switch

Nicholas A. Pease, Phuc H.B. Nguyen, Marcus A. Woodworth, Kenneth K.H. Ng, Blythe Irwin, Joshua C. Vaughan, and Hao Yuan Kueh

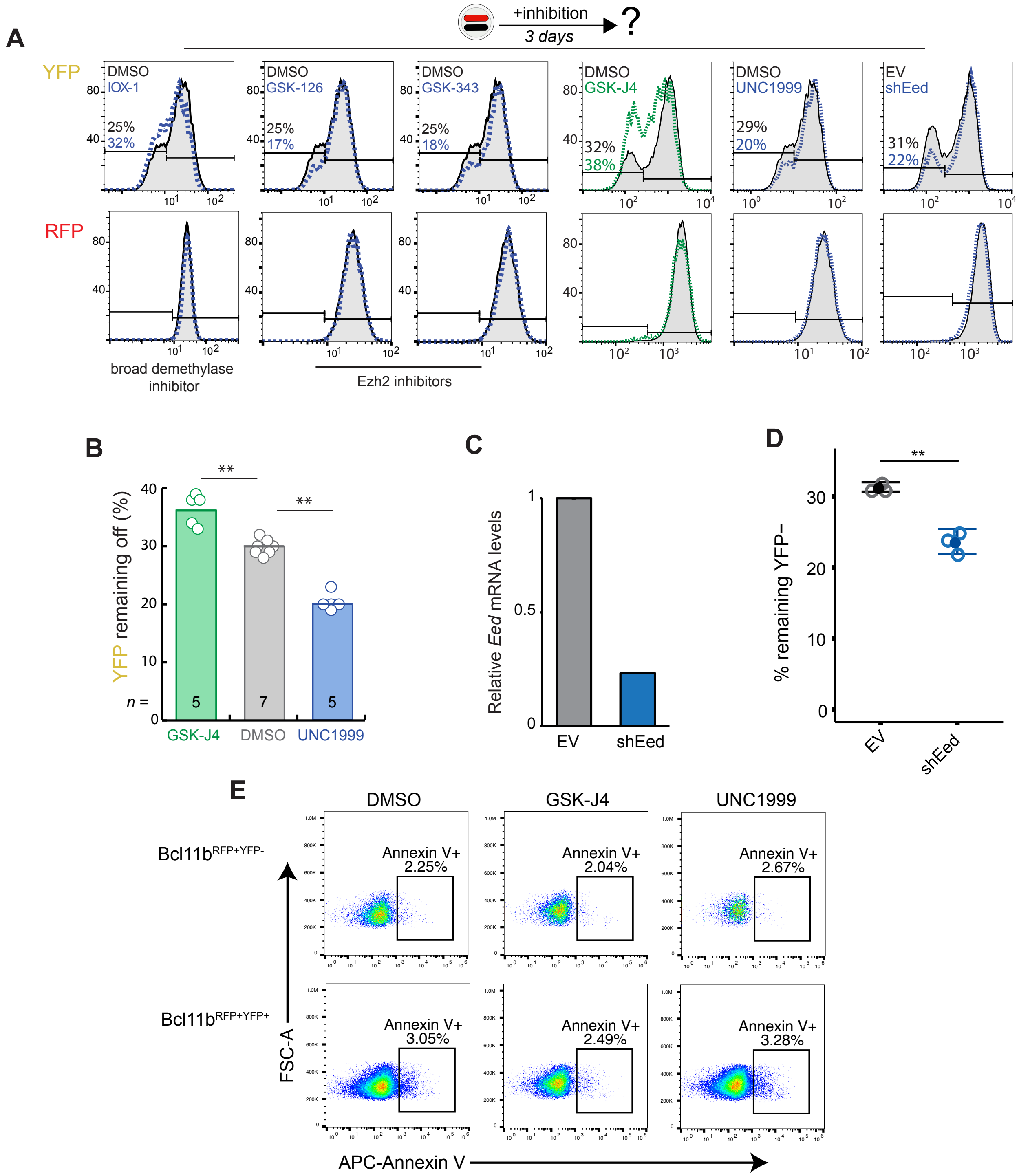

Supplementary Figure S1. Unrelated H3K27me3 perturbations modulate *Bcl11b* activation timing, related to Figure 2. (A) DN2 Bcl11b$^{RFP+YFP-}$ monoallelic expressing cells were sorted, re-cultured on OP9-DL1 cells in the presence of different small molecule inhibitors and analyzed by flow cytometry 3 days later. Structurally unrelated inhibitors show similar effects of decreasing or increasing *Bcl11b* activation probabilities as observed in Figure 2D (left). All histograms show that while the perturbations affect the all-or-none activation probability for the initially inactive alleles (top), the pertubations have no effect on the expression maintenance nor magnitude of the initially active alleles (bottom). (B) Mean percentage of cells remaining Bcl11b YFP-negative (n = independent experiments, *p < 0.05, **p < 0.01, two-sample t-test, two-tailed). (C) Relative mRNA levels of Eed were measured by qPCR. (D) Mean percentage of cells remaining YFP-negative after DN2 Bcl11b$^{RFP+/YFP-}$ monoallelic cells were transduced with retroviral constructs and recultured for 3 days (two-sample t-test, one-tailed, **p < 0.01, n = 3 independent experiments, error bars = 95% confidence interval). (E) Progenitors from Figure 2D (day 3) were stained with Annexin V to detect apoptotic cells.

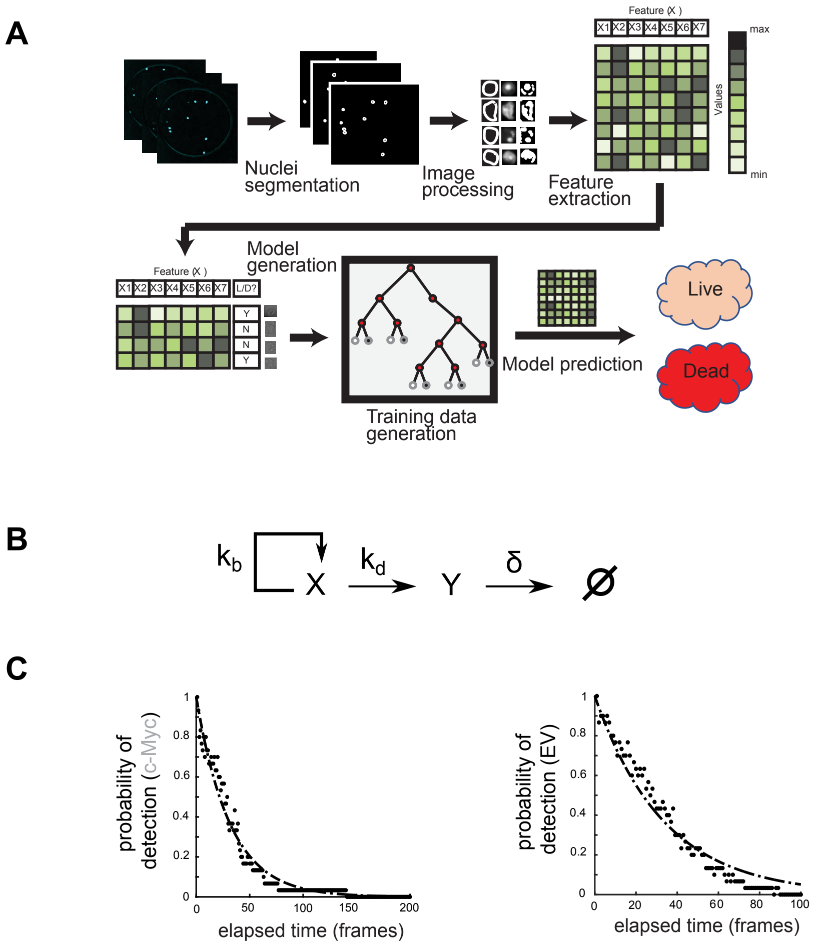

Supplementary Figure S2. Image processing pipeline for identification of live and dead cells in imaging experiments, related to Figure 3. (A) Cyan fluorescent protein (CFP) signals from transduced cells are used for segmentation of individual cells. Segmented populations were analyzed using image processing tools in MATLAB, and specific features such as object area, perimeter, and CFP intensity were collected. Approximately 10% of the total individual cells were then manually labeled as live or dead and fed into classification tree machine learning algorithm to generate a classification model. The rest of segmented cells are classified subsequently via the trained model. (B) Mathematical model describing the population dynamics of $Bcl11b^{RFP+/YFP-}$ cells transduced with c-Myc and empty vector (EV). The model includes a population of live cells (X) with a birth rate k_b and a death rate k_d that generates the dead cell population (Y). This population then has a permanent clearance rate of δ, indicating the process in which CFP degrades in these cells and the dead cells become undetected. (C) Experimentally determined decay of dead cells' detectability in time-lapsed movies. Individual dead cells were followed until their CFP level completely diminished and was undetectable by the segmentation algorithm, and the elapsed time was recorded. Thirty different individuals cells were recorded for each c-Myc and EV populations. Data was fit to an exponential decay function $P(\tau) = e^{-\delta\tau}$ with $\delta = 0.032$ per frame for c-Myc and $\delta = 0.030$ per frame for EV population.

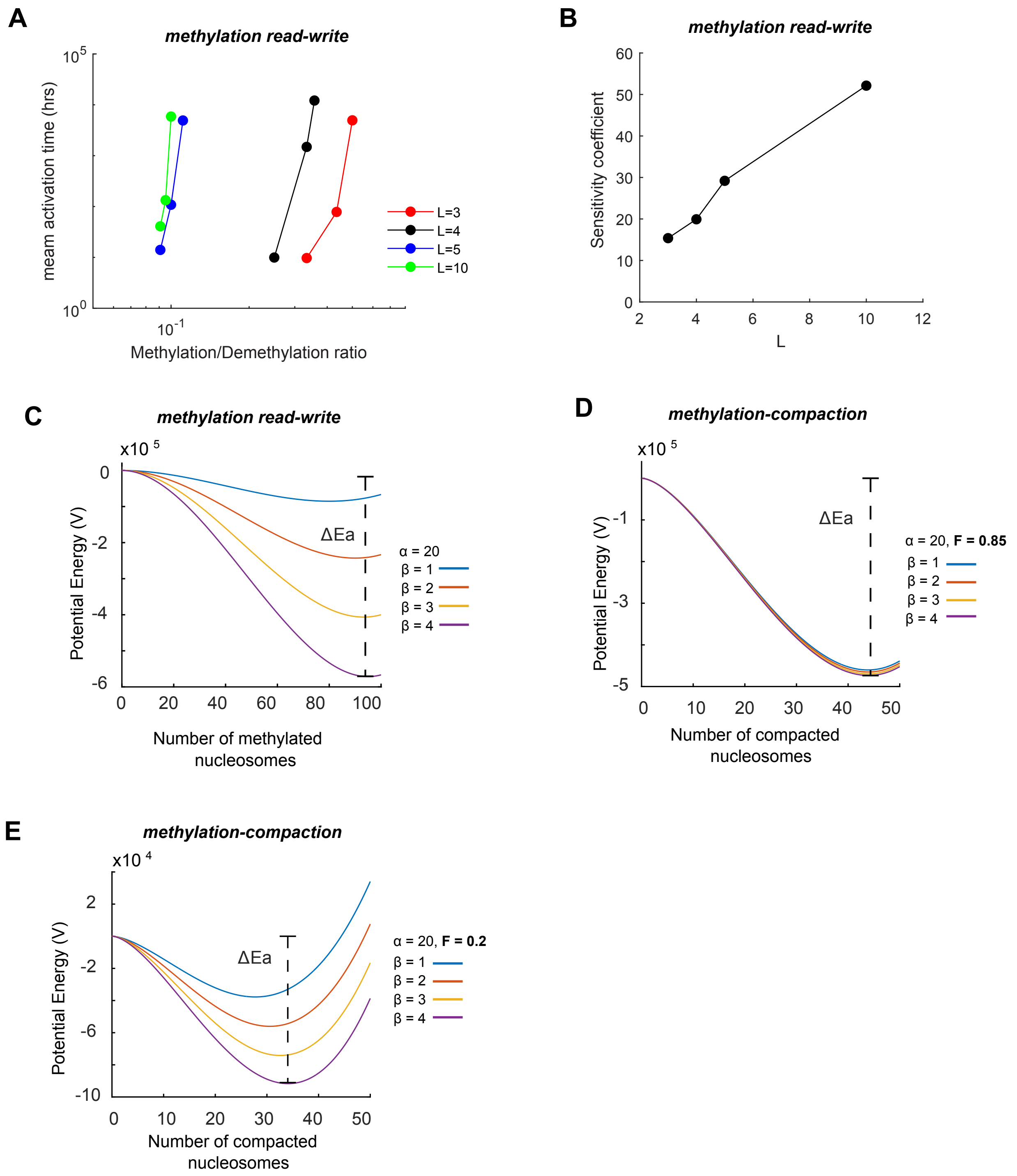

Supplementary Figure S3. Activation energy is robust to changes in methylation rate when interaction affinities between methylated and demethylated nucleosomes are similar, related to Figure 4. (A) Mean activation time as a function of the methylation/demethylation ratio derived from methylation read-write model simulations while decreasing the number of nulceosomes within 'reach' of a the PRC2 complex, L. (B) Sensitivity coefficient (ΔlogY/ΔlogX) as a function of L. (C) Potential energy landscapes of methylation read-write model. (D-E) Potential energy landscapes of the methylation-compaction model. Parameter F dictates how sensitve nucleosome compaction affinity is to demethylation (i.e. when F is high, the compaction affinity is only moderately affected by demethylation; see Mathematical Appendix for more details). The activation energy barrier (Ea) is defined as the potential energy (V) height between the local maximum and local minimum of the potential energy landscape. Each potential curve was plotted with demethylation parameter set to 20 hrs^{-1} and methylation rate parameter as indicated by the curve's color see Mathematical Appendix).

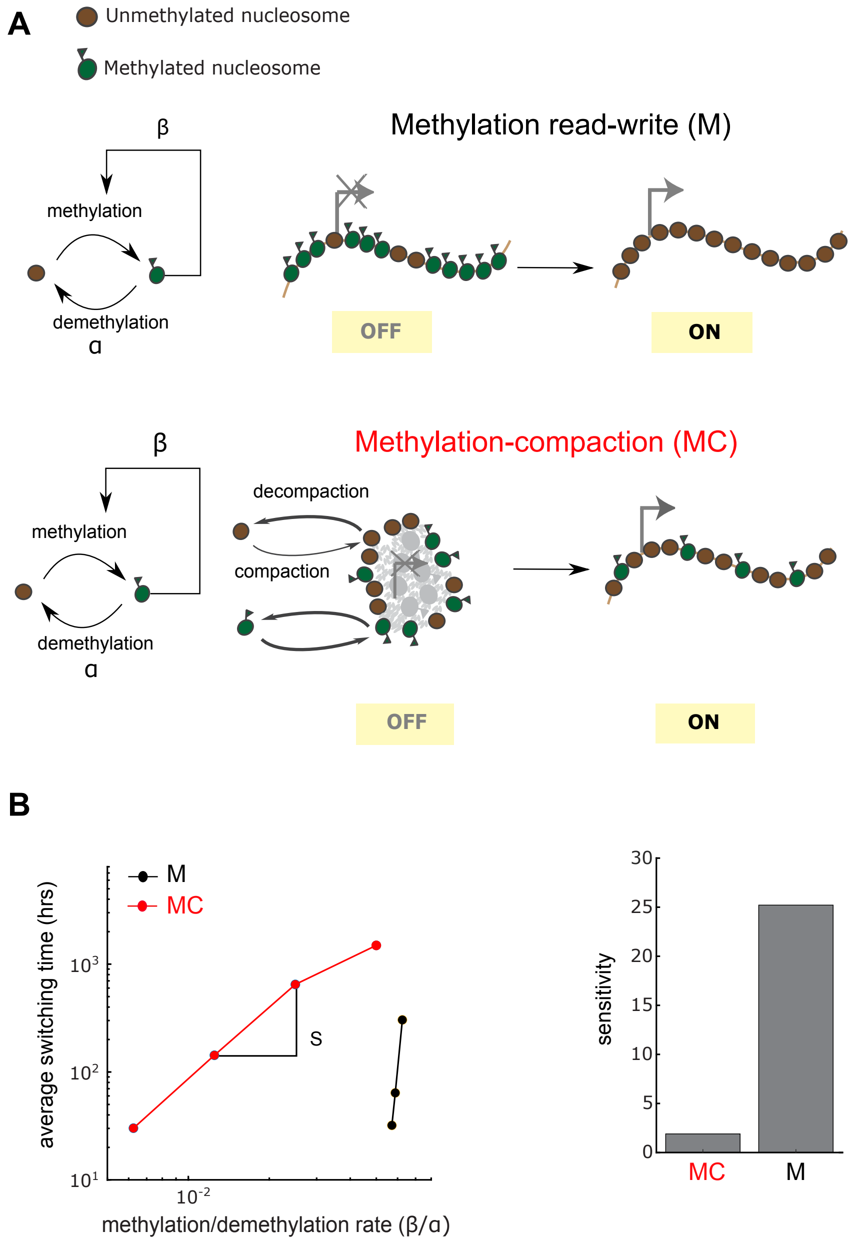

Supplemental Figure S4. Cooperativity in methylation-compaction model increases switching time tunability compared to methylation read-write model, related to Figure 4. (A, top) Methylation read-write model enables gene activation via complete eviction of methylation marks. (A, bottom) Methylation compaction model with cooperative methylation rate. A nucleosome's methylation rate increases with the number of methylated nucleosomes in the system. (B) Average switching times as a function of methylation (β) and demethylation rate constant (α) ratio for the methylation read-write (black) and methylation-compaction (red) models. Sensitivity coefficient (ΔlogY/ΔlogX) for each plot was calculated by taking the slope of the linear fit y = ax+b for the methylation model data set and the last 5 data points for the compaction model.

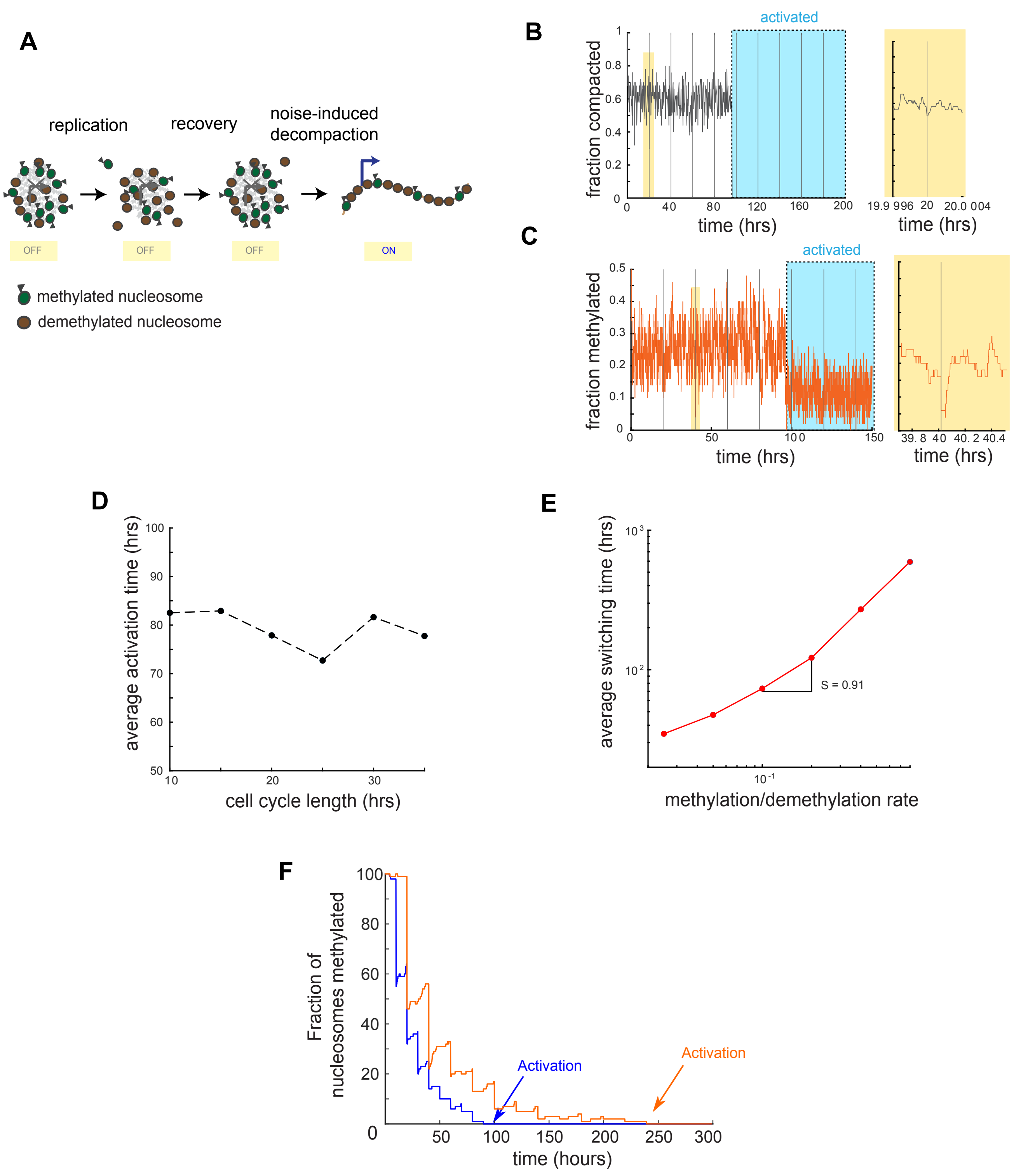

Supplementary Figure S5. Perturbations to the compacted state by DNA replication does not affect tunability or division-independence in the methylation compaction model, related to Figure 4. (A) Modified methylation compaction model where every cell division leads to 50% reduction in methylation state and 10% reduction in compaction state. (B-C) Compaction and methylation state as a function of time. Zoomed in first replication event. (D) Average switching time of the system as a function of cell cycle length. (E) Average switching times as a function of methylation and demethylation rate ratio. Tunability coefficient S ($\Delta \log Y/\Delta \log X$) for each plot was calculated by taking the slope of the linear fit $y = ax+b$ for the methylation model data set. (F) Fractions of methylated histones are shown for the methylation read-write model with cell division lengths set to be 10 hrs (blue) and 20 hrs (orange). Methylation and demethylation rates were set to 0.001 per hour (see Mathematical Analysis for more details).

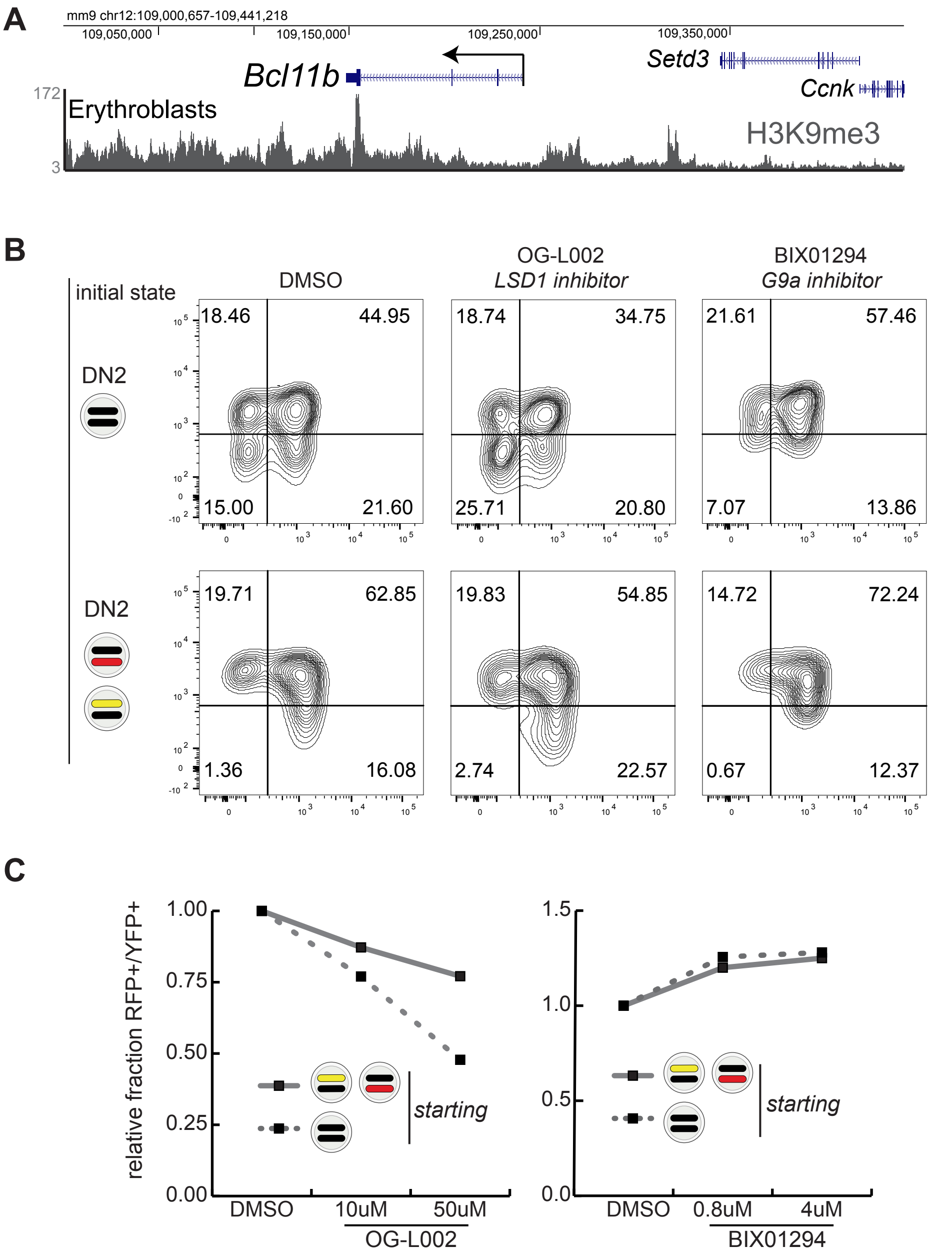

Supplemental Figure S6. H3K9-modifying enzymes regulate *Bcl11b* activation probability, related to Figure 4. (A) H3K9me3 ChIP-sequencing data from mouse erythroblasts (Davis et al., 2018; ENCODE accession ENCSR000DHN) visualized in the UCSC Genome Browser. (B) DN2 progenitors, either Bcl11b$^{RFP-/YFP-}$ (top) or Bcl11b$^{RFP-/YFP+}$ and Bcl11b$^{RFP+/YFP-}$ (bottom), were purified and re-cultured on OP9-DL1 cells for 3 days in the presence of OG-L002 (LSD1 inhibitor) or BIX01294 (G9a inhibitor) before analyzing reporter expression. (C) Relative fraction of progenitors RFP+/YFP+ after 3 days normalized to the respective DMSO controls.

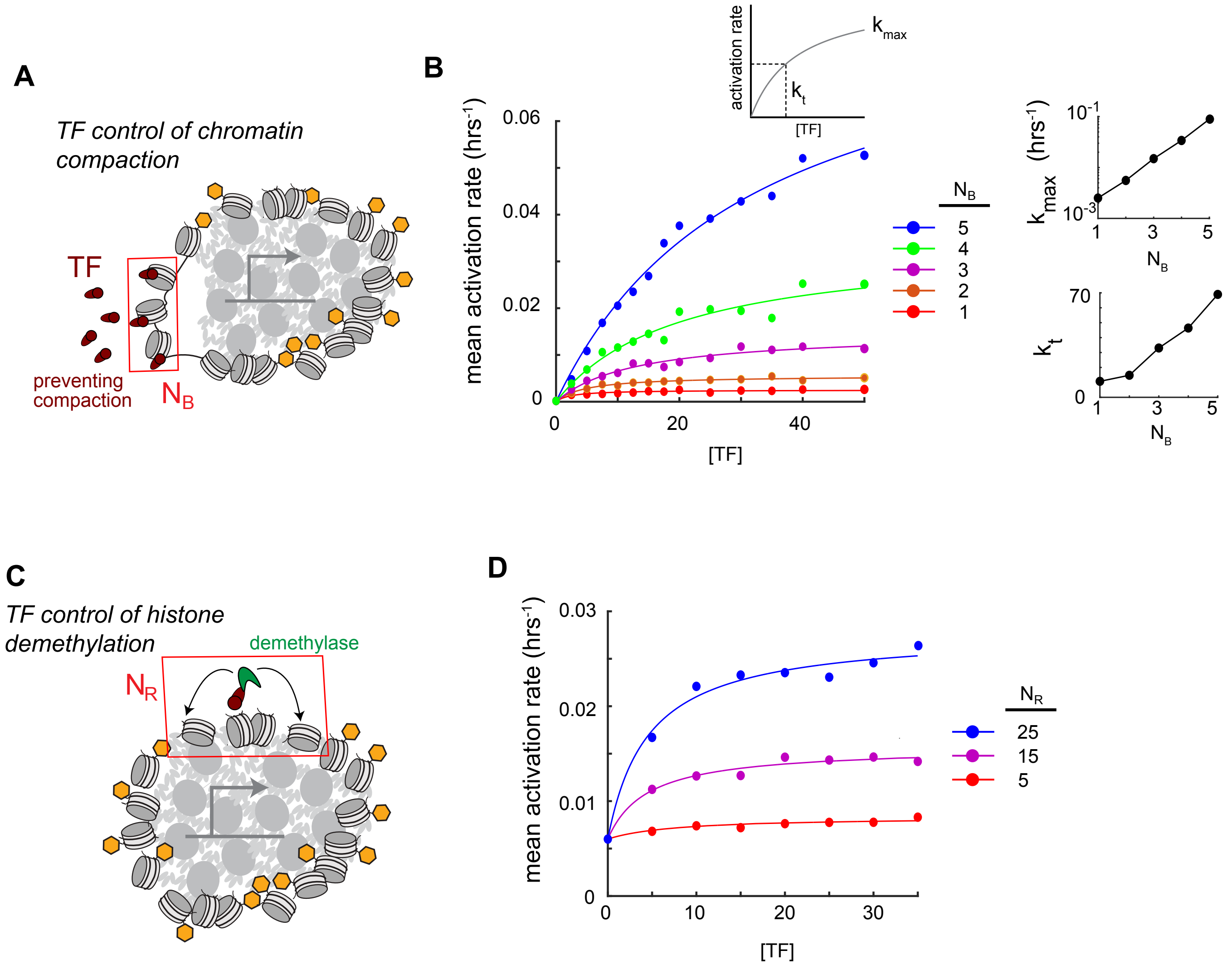

Supplementary Figure S7. Transcription factors can control gene activation timing by modulating nucleosome methylation or compaction, related to Figure 4. (A) In the MC model, pioneer TFs can bind to nucleosomes, preventing them from entering the compacted assembly. N_B indicates the number of TF binding sites, assumed to occur on different nucleosomes. (B) Mean simulated gene activation rate against TF concentration for different values of N_B (left). Maximal activation rate (k_{max}) and half-maximal concentration (k_t) as a function of the number of nucleosomes bound (N_B) (right). (C) TFs can also induce demethylation of a number of nucleosomes, N_R, within its vicinity of binding. (D) Mean gene activation rate as a function of TF concentration for different values of N_R.

	cMyc					
	Live Rate (*K*)	95% CI	Death Rate (k_d)	95% CI	Division Rate ($k_b = K + K_d$)	95% CI
Trial 1	0.031 hrs^{-1}	± 0.001	0.034 hrs^{-1}	± 0.001	0.065 hrs^{-1}	± 0.001
Trial 2	0.043 hrs^{-1}	± 0.001	0.013 hrs^{-1}	± 0.000	0.057 hrs^{-1}	± 0.001
Trial 3	0.035 hrs^{-1}	± 0.001	0.027 hrs^{-1}	± 0.001	0.069 hrs^{-1}	± 0.001

	EV					
	Live Rate (*K*)	95% CI	Death Rate (k_d)	95% CI	Division Rate ($k_b = K + K_d$)	95% CI
Trial 1	0.017 hrs^{-1}	± 0.001	0.028 hrs^{-1}	± 0.001	0.045 hrs^{-1}	± 0.001
Trial 2	0.014 hrs^{-1}	± 0.001	0.007 hrs^{-1}	± 0.001	0.027 hrs^{-1}	± 0.001
Trial 3	0.024 hrs^{-1}	± 0.001	0.019 hrs^{-1}	± 0.001	0.043 hrs^{-1}	± 0.001

Supplemental Table S1, related to Figures 3 and S2. Tabulated doubling (K) and death (k_d) rates calculated from data fitting of live and dead populations from three independent imaging experiments. Data was fit to population dynamics model described in Statistical and Quantitative Analysis section.

Methods S1: Mathematical Appendix, related to STAR Methods

Introduction

To understand the timed epigenetic switch controlling the *Bcl11b* activation, we used mathematical modeling to analyze a series of candidate biophysical mechanisms. This mathematical modeling analysis seeks to uncover the essential emergent properties of the switch, namely (1) its irreversible, all-or-none nature; (2) its long, stochastic time delay; (3) the heritability of its inactive and active states over DNA replication; and (4) its tunability with respect to changes in H3K27me3 levels and modifying-enzyme activity.

We consider two main candidate models. In the methylation read-write model (M), individual nucleosomes within in a one-dimensional lattice can be methylated or unmethylated. Gene expression is assumed to occur when the total fraction of methylated nucleosomes in this lattice falls below a threshold value. In the methylation compaction (MC) model, individual nucleosomes are also methylated and demethylated; in addition, these nucleosomes also interact to form a compacted assembly with rates dependent on their H3K27me3 state. Unlike the methylation read-write model, gene expression does not depend directly on H3K27me3 levels, but on the compaction state of the nucleosome assembly, which in turn depends on methylation states of individual nucleosomes. Both models explicitly model DNA replication as a process involving random segregation of modified nucleosomes into daughter strands. From our analysis, we find that the methylation-compaction mechanism, but not the methylation read-write mechanism, explains the emergent behaviors of the timed epigenetic switch controlling *Bcl11b* activation, and thus represents our favored model.

Model I: The Methylation Read-Write Mechanism (M)

Here, we adopt a standard framework for histone modification dynamics previously shown to generate multi-stability (Angel et al., 2011; Dodd et al., 2007). In this model, individual nucleosomes reside in a one-dimensional lattice, and exist in two states, a methylated state, corresponding to an H3K27 tri-methylated state, and demethylated state. We do not describe multiple demethylated states in our model (i.e. mono-methylation, di-methylation, and an un-methylated state), though our analysis, together with previous work (Dodd et al., 2007), indicates that our main conclusions should also hold in more complex models with additional states. As with previous models, the methylation rate of a given nucleosome depends on the number and distance of methylated nucleosomes in its vicinity, reflecting observations that PRC2 can bind and be activated by H3K27me3-marked nucleosomes to write H3K27me3 on neighboring nucleosomes. The positive feedback generated by this methylation read-write mechanism provides a basis for bi-stabilty in this model. Here, demethylation is taken to occur at a first order rate. We assume there is no spontaneous methylation in the absence of existing methylated nucleosomes; thus, once all nucleosomes are demethylated, the system irreversibly enters an activated state.

Methylation. We explicitly model mark binding and methyltransferase activities of the PRC2 complex, as well as the methylation state of each individual nucleosome. Take the gene locus to a linear array of N nucleosomes. Let $i = 1..N$ denote the index for the ith nucleosome, and let p_i be its H3K27 methylation state. $p_i = 0$ denotes the de-methylated state while $p_i = 1$ denotes the methylated state.

Let u' and u denote the transitions between the methylation state and demethylation state, respectively. The model is set up as follows:

For $i \in \{1, .. , N\}$:

$$u': (p_i = 0) \rightarrow (p_i = 1)$$

$$u: (p_i = 1) \rightarrow (p_i = 0)$$

With:

$$Pr(u') = \beta \cdot (1 - p_i) \cdot \sum_{j \neq i} \quad p_j \cdot e^{-\left(\frac{j-i}{L}\right)^2} \qquad (1)$$

$$Pr(u) = \alpha \cdot p_i \qquad (2)$$

The parameter L can be interpreted as the 'reach' of the PRC2 complex to neighboring nucleosomes. A large value of L indicates a long length scale for nucleosome interactions. This effect is set to have a gaussian shape so that nucleosome closest to the anchored PRC2 complex has the highest methylation rate. Similar distributions of activity have been reported for artificially tethered enzymes (Hass et al., 2015), as well as for histone modifications around transcription factor binding sites (Heinz et al., 2010). Moreover, we assume periodic boundary conditions for the one-dimensional lattice, though similar results were observed with other non-repeating boundary conditions (not shown).

Cell division. To model the transmission of histone marks across cell divisions, we assume that methylated nucleosomes segregate randomly to the two daughter DNA strands upon replication; thus, each nucleosome position has a one-half probability of inheriting a nucleosome that is methylated. Experimental evidence suggests that approximately half of total global H3K27me3 partitioning of parental marks to the subsequent generations (Alabert et al., 2015).

From stochastic simulations of this model, we find that this methylation read-write mechanism can generate a time-delayed, stochastic switches from an inactive H3K27me3-high state to an active state without H3K27me3 (Fig. 4A); however, switching times are hypersensitive to mild changes to methylation and de-methylation rates (Fig. 4B), and therefore inconsistent with the graded changes in switching times observed upon inhibition of PRC2 methyltransferase or Kdm6a/b demethylases (Fig. 2). To understand the origins of this hypersensitivity, we re-formulate this model using a chemical kinetics framework amenable to analysis using transition state theory. To do so, we first consider the limit where $L \rightarrow \infty$, such that each H3K27me3-bound PRC2 methylates all other un-methylated nucleosomes with the same reaction rate. In this limit, we can completely describe the state of the system by a single variable, the number of methylated nucleosomes *N'*. As the rates of adding or subtracting one methylated nucleosome from the system would reduce to become a function of *N'*, independent of spatial arrangement. Consequently:

$$N \underset{k_2}{\overset{k_1}{\rightleftarrows}} N'$$

where

$$k_1 = \beta N'(N_T - N') \qquad (3)$$

$$k_2 = \alpha N' \qquad (4)$$

and N_T is the total number of nucleosomes. The master equation describing the time evolution of this system is given by:

$$\frac{\partial p_n}{\partial t} = -[k_1(n) + k_2(n)] \cdot p_n + k_1(n-1)p_{n-1} + k_2(n+1)p_{n+1} \qquad (5)$$

where p_n is the probability of having N' methylated nucleosomes. When the total number of nucleosomes is large, we can approximate the number of methylated nucleosomes to be a continuous variable x. In this limit, we can rewrite the master equation as Fokker-Planck equation:

$$\frac{\partial p(x,t)}{\partial t} = \frac{\partial}{\partial x}[v(x)p(x)] + \frac{1}{2} \cdot \frac{\partial^2}{\partial x^2}[D(x)p(x)] \qquad (6)$$

where, we have ignored third and higher order terms, and where:

$$v(x) = k_1(x) - k_2(x) \qquad (7)$$

$$D(x) = k_1(x) + k_2(x) \qquad (8)$$

Given the velocity and diffusion constants for this system as a function of methylated nucleosome number, the switching of the system is essentially given by the first-passage time of the system to reach the absorbing state $x = 0$. A closed-form solution of this first-passage time distribution for the given rate functions is hard to obtain; Nevertheless, we note that our system operates in the regime where the timescales of individual methylation and demethylation reactions are much shorter than switching times for this system. In this regime, switching times are well described by the Kramer's theory for escape of a Brownian particle over a potential well (Kramers, 1940), and would thus approximately scale exponentially with the height of a potential energy barrier. We can obtain the functional form of this potential barrier by relating it to the velocity function:

$$-\frac{dV}{dx} = v(x) \qquad (9)$$

From equations (3), (4), and (7), we can then integrate the system to explicitly derive the potential function:

$$V(x) = \frac{\beta}{3}x^3 + \frac{\alpha - \beta N_T}{2}x^2 + W \qquad (10)$$

Where W is an arbitrary number. A plot of $V(x)$ is demonstrated in Fig. S3C. The energy landscape possesses a local minimum at a nonzero value of x, indicating the metastable state. The landscape has a local maximum near $x = 0$. State switching occurs when the system reaches the absorbing state $x = 0$. Thus, we define E_a as the height in $V(x)$ between the local maxima and the metastable state minima. When we plotted this potential energy for different values of β (Fig. S3C), we found that moderate changes in β led to significant changes in potential well height. As switching rates scale roughly exponentially with well height, we would expect this system would show extreme sensitivity in switching times with respect to changes in methylation rate changes.

Model II: The Methylation Compaction Mechanism (MC)

Because the methylation read-write mechanism above does not account for the tunable characteristics of the *Bcl11b* activation timing switch, we considered a second model, where histone methylation facilitates interactions between nucleosomes to enable the stable maintenance of a repressed, compacted chromatin state at the *Bcl11b* locus. There are multiple mechanisms by which H3K27me3 could facilitate interactions between nucleosomes: H3K27me3 could recruit polycomb repressive complex 1 (PRC1), which could oligomerize through contacts on its Bmi1 or Phc subunits (Eskeland et al., 2010; Gray et al., 2016; Isono et al., 2013; Kahn et al., 2016), or undergo weak, multivalent interactions on its Cbx2 subunit that result in liquid-liquid phase separation (Howard, 2001; Larson et al., 2017). Alternatively, H3K27me3 could modulate affinities of weak multivalent interactions between nucleosomes (Gibson et al., 2019), and thereby modulate their ability to phase separate.

The methylation compaction model consists of two main modules: (1) a H3K27 methylation and demethylation mechanism, and (2) a dynamic chromatin decompaction mechanism linked to H3K27me3 modification state that ultimately underlies gene switching. In our description of compaction dynamics, we do not explicitly model the spatial extent of the compacted nucleosomal assembly; instead, we adopt a mean-field approach that is established in models of cytoskeletal polymer dynamics (Erickson and Pantaloni, 1981; Jackson and Berkowitz, 1980). With this approach, the numbers of un-methylated and methylated nucleosomes within a compacted assembly are given by C and C' respectively, along with those outside the assembly are given by D and D' respectively. As a result, the dynamical system is described by four states: 1) Compacted-Methylated 2) Compacted-Demethylated 3) Decompacted-Methylated and 4) Compacted-Demethylated:

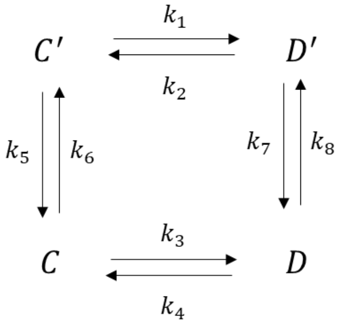

Here, C', C, D', and D denote the number of nucleosomes in these states, respectively. k_1 to k_8 denote the transition rates between them, which will be defined below. The gene is taken to be activated when all nucleosomes exist in a de-compacted state. The two mechanisms are intertwined so that methylation states affect compaction rates and vice versa. Detailed descriptions of the rates are given below:

Methylation. In this model, un-methylated nucleosomes convert into a methylated state with a first-order rate constant β. We assume this rate constant is the same regardless of whether nucleosomes are inside or outside the compacted assembly. Methylated nucleosomes convert into a de-methylated state with a rate constant of α if the nucleosome is outside the assembly (D'), or a lower rate constant of $f\alpha$, $(f < 1)$ if the nucleosome is inside the assembly. This lower rate constant

assumes that the demethylation reaction is less efficient on compacted nucleosomes, possibly due to competition for demethylase binding by compaction proteins, or due to the exclusion of demethylases through steric occlusion or phase separation. The rates describing these reactions on the four nucleosomal species are given by:

$$C' \underset{k_6}{\overset{k_5}{\rightleftarrows}} C \qquad D' \underset{k_8}{\overset{k_7}{\rightleftarrows}} D$$

Where:

$$k_5 = f\alpha\, C' \qquad (11)$$
$$k_6 = \beta\, C \qquad (12)$$

$$k_7 = \alpha\, D' \qquad (13)$$
$$k_8 = \beta\, D \qquad (14)$$

H3K27 methylation and demethylation rates are based on the catalytic activity of the Ezh2 subunit of the PRC2 complex and Kdm6a/b demethylases, respectively. Specifically, these rate constants were chosen to represent the conversion between H3K27me2 and H3K27me3. For simplicity, we do not model the H3K27me-binding dependent H3K27 methylation activity previously described (Margueron et al., 2009), though we show below that explicit modeling of this read-write effect would not significantly alter the conclusion of the model. Kdm6a/b demethylate H3K27me3 (Agger et al., 2007), and to our knowledge no cooperative activity of these complexes have not been reported.

Compaction. We adopt a mean-field description of the compacted nucleosomal assembly, following kinetic models of multi-stranded cytoskeletal polymer assembly (Howard, 2001). This description assumes that the nucleosome assembly is a roughly spherical structure held together by weak, multivalent interactions between individual nucleosomes, and can add or lose individual nucleosomes at its surface. Both methylated and demethylated nucleosomes can incorporate into the assembly; thus the assembly has a total size of:

$$C_T = C + C' \qquad (15)$$

where C and C' represent the number of methylated and demethylated nucleosomes in the assembly, respectively. Unlike other polymer models (MacPherson et al., 2018; Nuebler et al., 2018), we do not explicitly model physical connections between nucleosomes due to DNA; such connections would be expected to result in a spatial dependence of reaction rates within this chromatin domain; however, as the entire domain (100 nucleosomes) has a length scale greater than the persistence length of chromatin (~15-20 nucleosomes, from (Arbona et al., 2017)), and would thus enable free interactions between non-neighboring nucleosomes, we would expect the essential properties of our minimal model in a more realistic physical model that incorporates nucleosome connectedness.

The addition and removal of methylated and demethylated nucleosomes from the assembly is described by the following rate equations:

$$C' \underset{k_2}{\overset{k_1}{\rightleftarrows}} D' \qquad C \underset{k_4}{\overset{k_3}{\rightleftarrows}} D$$

Where:

$$k_1 = \frac{\delta}{C_T^{\frac{1}{3}}} C' \quad (16)$$

$$k_2 = \lambda\, C_T^{\frac{2}{3}} D' \quad (17)$$

$$k_3 = \frac{\delta}{C_T^{\frac{1}{3}}} C \quad (18)$$

$$k_4 = F\,\lambda\, C_T^{\frac{2}{3}} D \quad (19)$$

if $C_T > C_N$

$$k_1 = \frac{\delta}{C_T^{\frac{1}{3}}} C' \quad (20)$$

$$k_2 = 0 \quad (21)$$

$$k_3 = \frac{\delta}{C_T^{\frac{1}{3}}} C \quad (22)$$

$$k_4 = 0 \quad (23)$$

if $C_T < C_N$

Here, methylated nucleosomes incorporate into the compacted assembly with a rate constant λ; however, importantly, demethylated nucleosomes can also incorporate into the assembly with a reduced rate constant $F\lambda$ (where $F < 1$). The effect of methylation state on compaction rate is experimentally observed in instances such as recruitment of PRC1 complex by H3K27me3 marks (Kahn et al., 2016). The complex's subunits such as Ring1B and Phc-1 have been shown to be important in chromatin compaction and gene silencing (Eskeland et al., 2010; Francis et al., 2004; Isono et al., 2013). However, as PRC1 recruitment is not the only compaction mechanism *in vivo*, and because PRC1 can bind to nucleosomes independently of H3K27me3 (Francis et al., 2004), this model treats methylation as only a part, but not solely responsible for chromatin condensation. In choosing rate constants; we assume that compaction and decompaction is faster than histone methylation and demethylation rates, though timescales for both processes are assumed to be much faster than that for cell division. Fast compaction kinetics relative of modification is supported by *in vitro* studies of H3K27me3 methylation and demethylation kinetics, as well as *in vitro* DNA compaction by $HP1_{\alpha}$ and chromatin condensation experiments (Kristensen et al., 2011; Ladoux et al., 2000; Larson et al., 2017; Sneeringer et al., 2010).

The reaction rates for nucleosome incorporation (loss) scales with assembly size as $\sim C_T^{\frac{2}{3}}$ ($\sim C_T^{-\frac{1}{3}}$), as these reactions only take place on the surface of the assembly. Assuming a compacted nucleosome complex is spherical, the compaction rate would thus be proportional to the surface area. Likewise, the decompaction rate is also proportional to the surface area but reversely proportional to the total number compacted nucleosome in the complex.

In this description, there is a critical threshold number of compacted nucleosomes, C_N, below which the complex is thermodynamically unstable. The existence of a minimal nucleus size is a fundamental property of phase-separated assemblies held together by weak-multivalent, whereby addition of a new subunit to an already formed complex is thermodynamically more favorable than formation of the initial nucleus itself (Erickson and Pantaloni, 1981; Jackson and Berkowitz, 1980). Below this critical threshold C_N, the compacted assembly disintegrates, and gene turns on.

Cell division. Heritability of histone marks and chromatin states are crucial in maintaining gene expression states across cellular generations. As with the methylation read-write model above, we assume that methylated nucleosomes partition randomly between two daughter strands upon replication; the total number of nucleated nucleosomes is then obtained by sampling from binomial distribution with one half probability and N equal to the total number of nucleosomes at the point of DNA replication. Furthermore, we assume that compacted nucleosomes persist within a compacted assembly reside upon passage of DNA polymerase. This model feature assumes that new nucleosomes rapidly incorporate into a compacted assembly after passage of DNA polymerase; however, in the subsequent version of this model below, we will relax this assumption to allow for disruption of compaction state by DNA polymerase passage (see below).

From Monte-Carlo simulations, we found that this dynamic methylation compaction model can recapitulate all the essential emergent properties of the *Bcl11b* activation switch. Specifically, this model shows the following dynamic properties:

1) *Irreversible all-or-none switching to an H3K27me3-low, de-compacted state.* From simulations, we found that the system adopts a stable compacted assembly of nucleosomes with higher H3K27me3 marking density, but switches abruptly to a de-compacted state with lower H3K72me3 levels. As there is no re-nucleation of the compacted assembly after its elimination, this de-compacted state represents an absorbing, permanently active expressing state. The abrupt decrease in the H3K27me3 levels arises because compacted nucleosomes demethylate at a lower rate; thus, upon total decompaction, the percent of methylated nucleosomes lowers to a new steady state level.

2) *Noise induced gene activation.* Transition to the completely decompacted state, or gene activated state, occurs via stochastic deviation of the system from its compaction meta-stable state. Activation is triggered when the system reaches below the threshold number of compacted nucleosomes.

3) *Tunable activation rates*. The model is able to generate a gene switch with slow, tunable activation rate. Delay in activation is in order of days and can be finely adjusted by modifying methylation and demethylation rates, and/or changing H3K27me3 levels at the gene locus (Fig. 4E), as experimentally observed (Fig. 2). This ability to tune activation rates by changing H3K27me3 densities distinguishes this methylation compaction model from the methylation read-write model above, and thus represents a more plausible model for describing the activation mechanism of this switch. Why is this model uniquely tunable? In this model, locus de-compaction and gene activation are determined by a dynamic balance between rates of nucleosome entry or exit from a compacted assembly. The system still be sensitive to changes in these rates; however, as demethylated nucleosomes can still enter and exit a compacted assembly

at a reduced rate, changes in the fraction of demethylated nucleosomes would cause a fine change in these entry or exit rates, and thus give rise to a plausible tuning parameter for controlling activation timing.

4) *Division-independent timing control*. When the cell cycle length is changed in this model, activation kinetics remain largely unaffected, implying that the methylation-compaction mechanism functions as a cell division-independent delay timer. These conclusions hold, as long as the dynamic methylation and compaction mechanisms operate on timescales much faster than the cell cycle length.

To gain insights into the origins of tunability for the methylation compaction model, we adopt an approach, where we reduce this problem to using the Fokker-Planck approach, as utilized to analyze the methylation read-write mechanism (Fig. S3D,E). The full system with both methylation and compaction reactions would correspond to diffusive motion of a particle in a three-dimensional state space describing both chemical and physical states of nucleosomes. However, to simplify this problem to gain intuition, we will first take the methylation and demethylation reactions to be fast compared to the compaction and de-compaction reactions, such that the system can be described a single parameter C_T, corresponding to the total number of compacted nucleosomes. At any given time, the number of methylated and demethylated nucleosomes in the compacted state is at quasi-steady state, with values:

$$C' = \frac{\beta}{\beta + f\alpha} \cdot C_T \qquad (24)$$

and

$$C = \frac{f\alpha}{\beta + f\alpha} \cdot C_T \qquad (25)$$

Similarly, assuming that the system is at quasi steady state, the number of methylated and demethylated nucleosomes in the uncompacted state is given by:

$$D' = \frac{\beta}{\beta + \alpha} \cdot D_T \qquad (26)$$

and

$$D = \frac{\alpha}{\beta + \alpha} \cdot D_T \qquad (27)$$

Let $N_T = C_T + D_T$. With this approximation, the averaged rate of adding or removing a nucleosome from the compacted assembly is then given by:

$$k_{add} = k_2 + k_4 = \lambda C_T^{\frac{2}{3}} \frac{\beta}{\beta + \alpha} \cdot D_T + F \cdot \lambda C_T^{\frac{2}{3}} \frac{\alpha}{\beta + \alpha} \cdot D_T = \left(\frac{\beta}{\alpha} + F\right) \lambda C_T^{\frac{2}{3}} \cdot \frac{N_T - C_T}{1 + \frac{\beta}{\alpha}} \qquad (28)$$

$$k_{remov} = k_1 + k_3 = \frac{\delta}{C_T^{\frac{1}{3}}} C' + \frac{\delta}{C_T^{\frac{1}{3}}} C = \delta C_T^{\frac{2}{3}} \qquad (29)$$

Let the total number of compacted nucleosomes C_T be x. By writing down the master equation for this system, and by further applying the Fokker-Planck approximation, as performed in (5) and (6) we then have:

$$\frac{\partial p(x,t)}{\partial t} = \frac{\partial}{\partial x}[v(x)p(x)] + \frac{1}{2} \cdot \frac{\partial^2}{\partial x^2}[D(x)p(x)] \qquad (30)$$

where:

$$v(x) = \delta x^{\frac{2}{3}} - \left(\frac{\beta}{\alpha} + F\right) \lambda x^{\frac{2}{3}} \cdot \frac{N_T - x}{1 + \frac{\beta}{\alpha}} \qquad (31)$$

$$D(x) = \delta x^{\frac{2}{3}} + \left(\frac{\beta}{\alpha} + F\right) \lambda x^{\frac{2}{3}} \cdot \frac{N_T - x}{1 + \frac{\beta}{\alpha}} \qquad (33)$$

As before, we define a potential energy for this system:

$$-\frac{dV}{dx} = v(x) \qquad (34)$$

The analytical solution for the potential energy $V(x)$ for the methylation compaction model is:

$$V(x) = \frac{3}{5}\left[\delta - \frac{N}{1 + \frac{\beta}{\alpha}}\left(\frac{\beta}{\alpha} + F\right)\lambda\right] x^{\frac{5}{3}} + \frac{3}{8} \cdot \frac{1}{1 + \frac{\beta}{\alpha}}\left(\frac{\beta}{\alpha} + F\right) \lambda x^{\frac{8}{3}} + W \qquad (35)$$

A plot of $V(x)$ is demonstrated in Fig. S3D-E. We found that increasing methylation rate results in a much more attenuated increase in activation energy E_a with the methylation compaction model. This confirms that the improved switching rate tunability in the MC model stems from the decreased sensitivity to changes in activation barrier height by methylation rate. This result intuitive explains why this system shows significantly more graded changes in switching times when methylation rates are changed.

This tunability of switching times with respect to histone methylation depends on the relative association strengths of demethylated and methylated nucleosomes for each other in forming a compacted assembly. In our initial simulations, demethylated nucleosomes show only a moderate decrease in affinity for other compacted nucleosomes relative to methylated nucleosome (*F = 0.85*). However, when the binding strength of a demethylated nucleosome is much weaker than that of a methylated nucleosome (*F = 0.2*), we find changes in potential well heights become more significant, indicating that the system loses its tunability with respect to methylation changes (see Fig. S6E-D). This prediction, that methylated and demethylated nucleosomes have comparable strengths of association for a compacted assembly agrees well with evidence that unmethylated nucleosomes can nonetheless aggregate through a variety of H3K27me-independent mechanisms (Larson et al., 2017; Strom et al., 2017).

Model II.1: The Methylation Compaction Mechanism, with Compaction Disrupted by Division (Fig. S5)

This version of the model includes modified cellular division process in which upon replication, 50% of methylated nucleosomes become demethylated and 10% of compacted nucleosomes become uncompacted. This exit of nucleosomes from a compacted assembly due to DNA replication reflects the possibility that as the DNA replication machinery enters the compacted nucleosomal structure, it creates decompaction 'defects' in the condensed locus because nucleosomes near the replication forks are replaced. However, we reason that such defect would have a small effect to the overall stability of the structure because, at any given time, the site of replication would only take up a small region of the entire compacted domain.

In order to simulate both changes in methylation and compaction at the point of DNA replication, we must describe probabilistically how each of the four nucleosomal species are affected: 1) The Compacted-Methylated species (C'); 2) the Compacted-Demethylated species (C); 3) the Decompacted-Methylated species (D'); and 4) the Compacted-Demethylated species (D). Since methylation state is reduced by 50%, approximately half of Decompacted-Methylated species is transferred to Decompacted-Demethylated pool. Similarly, on average, 10% of the Compacted-Demethylated species are transferred to Decompacted-Demethylated pool due to DNA replication. Compacted-Methylated species have 50% chance to demethylate and 10% chance to decompact. Assuming these are two independent processes, this species has 5% chance to convert into Decompacted-Demethylated or Decompacted-Methylated and 45% chance to become Compacted-Demethylated. These observations are implemented as follows:

Let vector $S = [S_1, S_2, S_3, \dots, S_n]$ be the result from sampling a multinomial distribution with probabilities $\pi_1, \pi_2, \pi_3, \dots, \pi_n$, where $\pi_1 + \pi_2 + \pi_3 + \cdots + \pi_n = 1$. Let $S_i(\pi_1, \pi_2, \pi_3, \dots, \pi_n)$ be the ith element of S and N be the sample size. Let c', c, d', d be the number of compacted-methylated, compacted-unmethylated, decompacted-methylated, and decompacted-unmethylated nucleosomes, respectively immediately preceding the cellular division event. Partitioning of each species occurs as follows:

$$C' = S_4(0.45,0.05,0.05,0.45) \qquad (36)$$

$$D' = S_1(0.5,0.5) + S_2(0.45,0.05,0.05,0.45) \qquad (37)$$

$$C = S_2(0.1,0.9) + S_1(0.45,0.05,0.05,0.45) \qquad (38)$$

$$D = d + S_2(0.5,0.5) + S_1(0.1,0.9) + S_3(0.45,0.05,0.05,0.45) \qquad (39)$$

From stochastic simulations (Fig. S5A-E), we find that this modified methylation compaction model shows similar dynamic characteristics compared to the original methylation compaction model (Model II): it shows stochastic, all-or-none switching between inactive and active states; has an activation delay that can be tuned by changing H3K27me levels and enzyme activity; and shows division-independence in its activation time delay. Thus, we conclude that the essential features of

this model hold, even upon mild disruption of the inactive, compacted assembly by passage of DNA polymerase.

Model II.2: The Methylation Compaction Mechanism with Cooperative Methylation (Fig. S4)

PRC2 is known to be allosterically activated by H3K27me3 binding via its EED subunit (Margueron et al., 2009). Here, we consider this cooperative property of PRC2 by specifying that methylation rate increases with the total number of methylated nucleosomes in the model system. This assumption is likely valid when the number of nucleosomes in the condensed structure is small, and all the nucleosomes are more or less in close proximity with each other. To simulate this, we modified the methylation rates K_6 and K_8 so that their magnitude has a spontaneous term μ and the cooperative term β that is proportional to the total number of methylated species in the simulation:

$$C' \underset{k_6^*}{\overset{k_5}{\rightleftarrows}} C \qquad D' \underset{k_8^*}{\overset{k_7}{\rightleftarrows}} D$$

$$k_6{}^* = [\mu + \beta(C' + D')]C \qquad (40)$$

$$k_8{}^* = [\mu + \beta(C' + D')]D \qquad (41)$$

From stochastic simulations (Fig. S4), we find that this system is also capable of generating long, stochastic delays in all-or-none switching in locus compaction state, and that switching times can be finely tuned by changing H3K27me3 levels, as with our simpler methylation compaction model (Model II). We conclude that incorporation of a cooperative H3K27me3 methylation rate in our methylation compaction model does not alter its main conclusions.

Model II.3: Transcription factor tuning in the methylation-compaction model (Fig. 5)

Model II.3.1. Transcription factors prevent chromatin compaction

Here, we first consider a scenario where the transcription factors prevent a small number of nucleosomes from compaction. This can be accomplished by designating a small portion of nucleosomes to carry binding sites for the transcription factors. Upon binding to these factors, these nucleosomes can no longer associate with other nucleosomes in the compacted assembly:

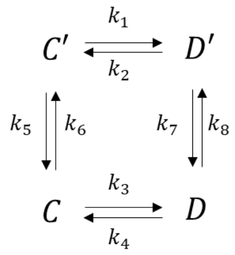

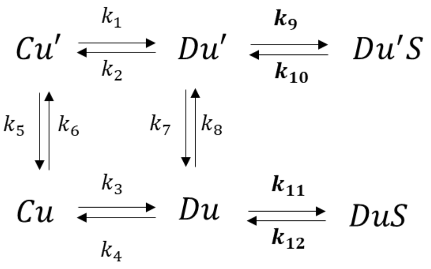

The nucleosomes carrying transcription factor binding sites are labeled *Cu, Cu', Du,* and *Du'*. Reaction rates $k_{1\text{-}8}$ are the same as the standard model. When these nucleosomes are decompacted, they can bind to the transcription factors with the following rates:

$$k_9 = K_{ON} \cdot TF \cdot Du' \qquad (42)$$

$$k_{10} = K_{OFF} \cdot Du'S \qquad (43)$$

$$k_{11} = K_{ON} \cdot TF \cdot Du \qquad (44)$$

$$k_{12} = K_{OFF} \cdot DuS \qquad (45)$$

Here $[TF]$ is the transcription factor concentration. Let C_T be the total number of compacted nucleosomes and let N_B be number of nucleosomes that carry transcription factor binding sites. We run our simulation with the assumption that the total number of nucleosomes is constant, and N_B can be varied.

From simulations (Fig. 5A), we find that activation timing can be tuned with the transcription factor copy number. Additionally, activation rate increases synergistically with number of binding sites. Therefore, timing modulation can be achieved via transcription factor acting as nucleosome sequester.

Model II.3.2. Transcription factor induce histone demethylation

We next consider an alternative mechanism where a transcription factor is recruited to the nucleosome assembly at a single site. Once bound, the transcription factor acts as a demethylase to removes methyl marks on the nucleosomes in its vicinity:

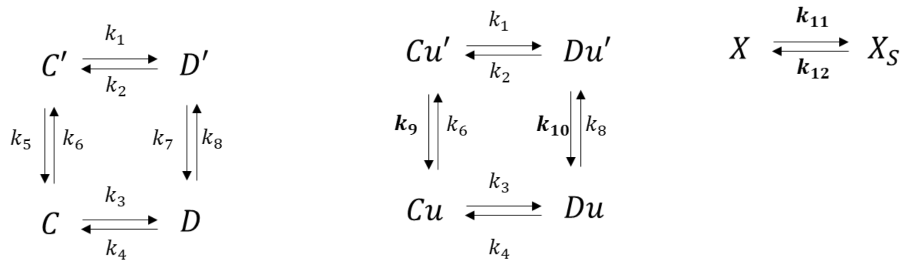

Here *Cu, Cu', Du,* and *Du'* are nucleosomes that are in the recruited demethylase's reach. The unrecruited demethylase is denoted as X, and X_S is the recruited transcription factor–bound demethylase. Since the simulation involves one nucleosomal array, the number of demethylases is limited to 1, such that $X + X_S = 1$. Conversion rate probability between X and X_S and the demethylation rate of the nucleosomes inside the reach of the methylase are described below:

$$k_9 = \alpha_{TF} \cdot X \cdot f \cdot Cu' \qquad (46)$$

$$k_{10} = \alpha_{TF} \cdot X_S \cdot Du' \qquad (47)$$

$$k_{11} = K_{ON} \cdot TF \cdot X \qquad (48)$$

$$k_{12} = K_{OFF} \cdot X_S \qquad (49)$$

Here, $\alpha_{TF}$is the transcription factor recruited demethylation rate constant, and f is the fraction reduction of the rate when the nucleosome is decompacted. In our simulations, C_T is the total number of compacted nucleosomes, as before, and N_R is the maximum number of nucleosomes that can be affected by the recruited demethylase. We run our simulation varying N_R, while keeping the total number of nucleosomes is constant. From simulations, we find that, unlike transcription factors that impact chromatin compaction, transcription factors that recruit demethylase according to this mechanism requires a large effective range to appreciably tune switching rate (Figure 5D).

Parameter List

a. Methylation Read-Write Model (Figure 4 A-B)

Pure Methylation Model			
Parameters	Description	Value	Comments/references
β_{ON}	cooperative methylation rate constant	1 hrs^{-1}	Sneeringer et al., 2010
α_{OFF}	demethylation rate constant	16.5 hrs^{-1}	[16 , 17 , 17.5] for parameter scan
			Kristensen et al., 2011
L	reach of anchored methylating enzyme to neighboring nucleosome	15	Hass et al., 2015
N	number of simulated nucleosomes	100	
cell division length	cell division length	20 hrs	

b. Compaction Methylation Model (Figure 4 C-D)

Compaction Methylation Model			
Parameters	Description	Value	Comments/references
β	methylation rate constant	1 hrs^{-1}	[0.2 , 0.4 , 0.8 , 1.6 , 3.2 , 6.4] for parameter scan
			Sneeringer et al., 2010
α	demethylation rate constant	8 hrs^{-1}	Kristensen et al., 2011
f	fraction of de-methylation rate when nucleosome is in compacted state	0	
λ	compaction rate constant	310 hrs^{-1}	Larson et al., 2017; Ladoux et al., 2000
δ	decompaction rate constant	5300 hrs^{-1}	Larson et al., 2017; Ladoux et al., 2000
F	fraction of compaction rate when nucleosome is in demethylated stated	0.85	
N	Number of simulated nucleosomes	50	
C_N	nucleation threshold for compacted nucleosomal complex	5	
cell division length	cell division length	20 hrs	

c. Cell division dependence of Pure Dilution vs Compaction Methylation Models (Figure 4 F-G)

Pure Dilution Model			
Parameters	Description	Value	Comments/references
β_{ON}	cooperative methylation rate constant	0	
α_{OFF}	demethylation rate constant	0	
L	reach of anchored methylating enzyme to neighboring nucleosome	0	
N	number of simulated nucleosomes	100	
cell division length	cell division length	20 hrs	[10 , 15 , 20 , 25 , 30 , 35] for cycle scan
**Compaction Methylation Model**			
Parameters	Description	Value	Comments/references
β	methylation rate constant	1 hrs^{-1}	Sneeringer et al., 2010
α	demethylation rate constant	8 hrs^{-1}	Kristensen et al., 2011
f	fraction of de-methylation rate when nucleosome is in compacted state	0	
λ	compaction rate constant	310 hrs^{-1}	
δ	decompaction rate constant	5300 hrs^{-1}	
F	fraction of compaction rate when nucleosome is in demethylated stated	0.85	
N	Number of simulated nucleosomes	50	
C_N	nucleation threshold for compacted nucleosomal complex	5	
cell division length	cell division length	20 hrs	[10 , 15 , 20 , 25 , 30 , 35] for cycle scan

d. Effects of Transcription Factors on Methylation Compaction Model's Activation Timing (Figure 5)

Transcription Factor Affecting Compaction Model			
Parameters	Description	Value	Comments/references
β	methylation rate constant	6.4 hrs^{-1}	Sneeringer et al., 2010
α	demethylation rate constant	8 hrs^{-1}	Kristensen et al., 2011
f	fraction of de-methylation rate when nucleosome is in compacted state	0	
λ	compaction rate constant	315 hrs^{-1}	Larson et al., 2017; Ladoux et al., 2000
δ	decompaction rate constant	5300 hrs^{-1}	Larson et al., 2017; Ladoux et al., 2000
F	fraction of compaction rate when nucleosome is in demethylated stated	0.85	
N	number of simulated nucleosomes	50	
C_N	nucleation threshold for compacted nucleosomal complex	5	
K_{ON}	transcription factor association constant	1 hrs-1	
K_{OFF}	transcription factor dissociation constant	5 hrs-1	
TF	transcription factor concentration	0-100	
N_B	total number of nucleosomes carrying transcription factor binding sites	1-5	
cell division length	cell division length	20 hrs	
**Compaction Methylation Model With Permanently demethylated Nucleosomes**			
Parameters	Description	Value	Comments/references
β	methylation rate constant	6.4 hrs^{-1}	Sneeringer et al., 2010
α	demethylation rate constant	8 hrs^{-1}	Kristensen et al., 2011
f	fraction of de-methylation rate when nucleosome is in compacted state	0	
λ	compaction rate constant	295 hrs^{-1}	Larson et al., 2017; Ladoux et al., 2000
δ	decompaction rate constant	5300 hrs^{-1}	Larson et al., 2017; Ladoux et al., 2000
F	fraction of compaction rate when nucleosome is in demethylated stated	0.85	
N	Total number of simulated nucleosomes	50	
C_N	nucleation threshold for compacted nucleosomal complex	5	
K_{ON}	transcription factor association constant	1 hrs-1	
K_{OFF}	transcription factor dissociation constant	5 hrs-1	
TF	transcription factor concentration	0-35	
N_B	total number of nucleosomes carrying transcription factor binding sites	5-25	
α_{TF}	recruited demethylase rate constant	1600 hrs-1	
cell division length	cell division length	20 hrs	

e. Methylation model with different cooperative reach parameter L (Supplementary Figure 3A-B)

Pure Methylation Model			
Parameters	Description	units	values
β_{ON}	cooperative methylation rate constant	hrs^{-1}	1
α_{OFF}	demethylation rate constant	hrs^{-1}	[2, 2.3, 3] for L = 3
			[2.8, 3, 4] for L = 4
			[9, 10, 11] for L = 5
			[10, 10.5, 11] for L = 10
L	reach of anchored methylating enzyme to neighboring nucleosome	nucleosomes	[3, 4, 5 ,10]
N	number of simulated nucleosomes	nucleosomes	100
cell division length	cell division length	hrs	20

f. Potential energy landscapes analysis for pure methylation model and methylation compaction model (Supplementary Figure 3C - E)

Pure Dilution Model		
**Parameters**	**Description**	**Value**
β	cooperative methylation rate constant	1-4 hrs^{-1}
α	demethylation rate constant	20 hrs^{-1}
N	number of simulated nucleosomes	100
W	arbitrary constant	100
**Compaction Methylation Model**		
**Parameters**	**Description**	**Value**
β	methylation rate constant	1-4 hrs^{-1}
α	demethylation rate constant	20 hrs^{-1}
λ	compaction rate constant	31 hrs^{-1}
δ	decompaction rate constant	530 hrs^{-1}
F	fraction of compaction rate when nucleosome is in demethylated stated	0.85
N	Number of simulated nucleosomes	50

g. Compaction with Cooperative Methylation Model (Supplementary Figure 4)

Compaction Cooperative Methylation Model			
**Parameters**	**Description**	**Value**	**Comments/references**
β	cooperative methylation rate constant	0.02 hrs^{-1}	
μ	spontaneous rate constant	[0.05 , 0.1 , 0.2 , 0.4] hrs^{-1}	
α	demethylation rate constant	8 hrs^{-1}	Kristensen et al., 2011
f	fraction of de-methylation rate when nucleosome is in compacted state	0	
λ	compaction rate constant	310 hrs^{-1}	Larson et al., 2017; Ladoux et al., 2000
δ	decompaction rate constant	5300 hrs^{-1}	Larson et al., 2017; Ladoux et al., 2000
F	fraction of compaction rate when nucleosome is in demethylated stated	0.85	
N	number of simulated nucleosomes	50	
C_N	nucleation threshold for compacted nucleosomal complex	5	
cell division length	cell division length	20 hrs	
**Pure Methylation Model**			
**Parameters**	**Description**	**Value**	**Comments**
$β_{ON}$	cooperative methylation rate constant	1 hrs^{-1}	Sneeringer et al., 2010
$α_{OFF}$	demethylation rate constant	[16 , 17 , 17.5] hrs^{-1}	for parameter scan, Kristensen et al., 2011
L	reach of anchored methylating enzyme to neighboring nucleosome	15	Hass et al., 2015
N	number of simulated nucleosomes	100	
cell division length	cell division length	20 hrs	

h. Compaction Methylation Model with Compaction State Disruption by Cell Division (Supplementary Figure 5A-E)

Compaction Methylation Model with Compaction State Affected by Cell Division			
**Parameters**	**Description**	**Value**	**Comments/references**
β	methylation rate constant	1 hrs^{-1}	[0.2 , 0.4 , 0.8 , 1.6 , 3.2 , 6.4] for parameter scan in Supplemental Figure 2E<br>Sneeringer et al., 2010
α	demethylation rate constant	8 hrs^{-1}	Kristensen et al., 2011
f	fraction of de-methylation rate when nucleosome is in compacted state	0	
λ	compaction rate constant	310 hrs^{-1}	Larson et al., 2017; Ladoux et al., 2000
δ	decompaction rate constant	5300 hrs^{-1}	Larson et al., 2017; Ladoux et al., 2000
F	fraction of compaction rate when nucleosome is in demethylated stated	0.85	
N	Number of simulated nucleosomes	50	
C_N	nucleation threshold for compacted nucleosomal complex	5	
cell division length	cell division length	20 hrs	[10 , 20 , 25 , 30 , 35] for parameter scan in Supplemental Figure 2D

i. Pure Dilution Model with Minimal Methylation and Demethylation Rates (Supplementary Figure 5F)

Dilution Model With Minimal Enzymatic Activities			
**Parameters**	**Description**	**Value**	**Comments/references**
$β_{ON}$	cooperative methylation rate constant	0.001 hrs-1	
$α_{OFF}$	demethylation rate constant	0.001 hrs-1	
L	reach of anchored methylating enzyme to neighboring nucleosome	15	Hass et al., 2015
N	number of simulated nucleosomes	100	
cell division length	cell division length	10 and 20 hrs	
